# iPSC‐based modeling of THD recapitulates disease phenotypes and reveals neuronal malformation

**DOI:** 10.15252/emmm.202215847

**Published:** 2023-02-06

**Authors:** Alba Tristán‐Noguero, Irene Fernández‐Carasa, Carles Calatayud, Cristina Bermejo‐Casadesús, Meritxell Pons‐Espinal, Arianna Colini Baldeschi, Leticia Campa, Francesc Artigas, Analia Bortolozzi, Rosario Domingo‐Jiménez, Salvador Ibáñez, Mercè Pineda, Rafael Artuch, Ángel Raya, Àngels García‐Cazorla, Antonella Consiglio

**Affiliations:** ^1^ Neurometabolic Unit and Synaptic Metabolism Lab, Neurology Department Institut Pediàtric de Recerca, Hospital Sant Joan de Déu Barcelona Spain; ^2^ Department of Pathology and Experimental Therapeutics Bellvitge University Hospital‐IDIBELL, Hospitalet de Llobregat Barcelona Spain; ^3^ Institute of Biomedicine of the University of Barcelona (IBUB) Barcelona Spain; ^4^ Regenerative Medicine Program Bellvitge Biomedical Research Institute (IDIBELL) Barcelona Spain; ^5^ Program for Translation of Regenerative Medicine in Catalonia (P‐[CMRC]) Hospital Duran i Reynals, Hospitalet de Llobregat Barcelona Spain; ^6^ Institut d'Investigacions Biomèdiques de Barcelona (IIBB), Spanish National Research Council (CSIC) Barcelona Spain; ^7^ Institut d'Investigacions August Pi i Sunyer (IDIBAPS) Barcelona Spain; ^8^ Centro de Investigación Biomédica en Red de Salud Mental (CIBERSAM), ISCIII Madrid Spain; ^9^ Department of Pediatric Neurology Hospital Virgen de la Arrixaca Murcia Spain; ^10^ Instituto Murciano de Investigación Biosanitaria Virgen de la Arrixaca (IMIB) Murcia Spain; ^11^ Centro de Investigación Biomédica En Red Enfermedades Raras (CIBERER) Madrid Spain; ^12^ Fundació Sant Joan de Déu (FSJD), Hospital Sant Joan de Déu (HSJD) Barcelona Spain; ^13^ Metabolic Unit, Departments of Neurology, Nutrition Biochemistry and Genetics Institut Pediàtric de Recerca, Hospital San Joan de Déu Barcelona Spain; ^14^ Centre for Networked Biomedical Research on Bioengineering, Biomaterials and Nanomedicine (CIBER‐BBN) Madrid Spain; ^15^ Institució Catalana de Recerca i Estudis Avançats (ICREA) Barcelona Spain; ^16^ Department of Molecular and Translational Medicine University of Brescia Brescia Italy

**Keywords:** dopamine, iPSC, L‐Dopa, Parkinsonism, tyrosine hydroxylase deficiency, Genetics, Gene Therapy & Genetic Disease, Neuroscience

## Abstract

Tyrosine hydroxylase deficiency (THD) is a rare genetic disorder leading to dopaminergic depletion and early‐onset Parkinsonism. Affected children present with either a severe form that does not respond to L‐Dopa treatment (THD‐B) or a milder L‐Dopa responsive form (THD‐A). We generated induced pluripotent stem cells (iPSCs) from THD patients that were differentiated into dopaminergic neurons (DAn) and compared with control‐DAn from healthy individuals and gene‐corrected isogenic controls. Consistent with patients, THD iPSC‐DAn displayed lower levels of DA metabolites and reduced TH expression, when compared to controls. Moreover, THD iPSC‐DAn showed abnormal morphology, including reduced total neurite length and neurite arborization defects, which were not evident in DAn differentiated from control‐iPSC. Treatment of THD‐iPSC‐DAn with L‐Dopa rescued the neuronal defects and disease phenotype only in THDA‐DAn. Interestingly, L‐Dopa treatment at the stage of neuronal precursors could prevent the alterations in THDB‐iPSC‐DAn, thus suggesting the existence of a critical developmental window in THD. Our iPSC‐based model recapitulates THD disease phenotypes and response to treatment, representing a promising tool for investigating pathogenic mechanisms, drug screening, and personalized management.

## Graphical abstract

This is the first proof of principle that iPSC‐derived dopaminergic (DA) neurons from mild (THDA) and severe (THDB) patients reproduce distinct disease‐related features and reveal abnormal morphologies. The study highlights the importance of prenatal treatment with L‐Dopa in cases of THDB.

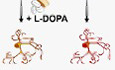

**The paper explained**

**Problem**

Tyrosine Hydroxylase deficiency (THD) is a neuropediatric disorder characterized by the lack of the enzyme tyrosine hydroxylase (TH) that is an important source of dopamine in the brain. Symptoms can resemble those of a movement disorder (phenotype called type A) but may also include those of a severe and widespread brain disorder (phenotype called type B). Mild and moderate forms of THD show dramatic improvement when treated with L‐Dopa, while patients with severe form of THD do not respond to treatment and present with cognitive impairment. Here, we generated a new iPSC‐based model for a better understanding of THD disease mechanisms and treatment response.

**Results**

We generated induced pluripotent stem cells (iPSCs) from two THD patients: one with a mild phenotype (THDA) and another with the severe phenotype (THDB). All of these iPSC lines were differentiated into dopaminergic neurons (DAn), the cell type of interest in this disease. Similar to what was observed in patients, the iPSC‐based THD model showed lower dopamine levels and reduced TH expression when compared to controls. Moreover, DAn differentiated from patient‐specific iPSC showed abnormal neuronal morphology. Most importantly, THD iPSC‐derived DAn also recapitulated the patient's response to treatment as we were able to rescue only the defects in THDA, but not in THDB. Moreover, if treatment was administered early, we could also prevent defects in THDB, suggesting a precise time in brain development when treatment should be applied. Thus, our findings represent a first direct indication that dysfunctional neurons play a crucial role during THD pathogenesis and may have broad implications for future intervention in early stages of THD disease.

**Impact**

This is the first iPSC‐based model of THD that brings together THDA and THDB patients and recapitulates pathological features of both disease types and response to treatment. Using this new model, we detected neuronal abnormalities that had never been revealed before. Thus, we believe that our iPSC‐based THD model provides a valuable tool to investigate the unknown pathogenic mechanisms of THD and to screen for drugs as well as for the development of novel therapies that may help for the management of all THD patients.

## Introduction

Tyrosine hydroxylase deficiency (THD) is an ultra‐rare autosomal recessive neurometabolic disorder resulting from cerebral enzyme tyrosine hydroxylase deficiency (TH; OMIM 191290). TH is required to catalyze the conversion of tyrosine to L‐Dopa, which is the enzymatic rate‐limiting step in catecholamine biosynthesis. THD is characterized by brain dopamine (DA) depletion, leading people affected by the disease to a progressive encephalopathy and often poor prognosis. Approximately 90 cases of THD have been described worldwide (Willemsen *et al*, 2010; Dong *et al*, 2020), but misdiagnosis rates are believed to be non‐negligible.

More than 40 disease‐causing mutations in the human *TH* gene have been identified (Willemsen *et al*, 2010; Furukawa & Kish, 2017), of which the most frequent are p.Arg233His and p.Leu236Pro (Willemsen *et al*, 2010). Clinical features include dystonia, intellectual disability, tremor, chorea, oculogyric crisis, eyelid ptosis, diurnal fluctuation of signs, and autonomic dysfunction.

The broad phenotypic spectrum observed in patients may be associated with a lack of a clear correlation between the genotype and phenotype since the same mutation, which occurs in homozygosity or compound heterozygosity, can be associated with mild or severe phenotype (Willemsen *et al*, 2010; Fossbakk *et al*, 2014). While children affected by the mild form of this disorder (type A) typically develop progressive rigid hypokinetic syndrome and dystonia with onset in infancy or childhood, severe TH deficiency (type B) causes a complex early‐onset encephalopathy with developmental delay and severe Parkinsonism (Willemsen *et al*, 2010; Furukawa & Kish, 2017).

Treatment with L‐Dopa and Carbidopa represents the first‐choice strategy for THD patients, although treatment responses vary between THD forms. Usually, patients with type A THD respond to this treatment by achieving normal cognitive and motor milestones, although cognitive development may be moderately abnormal in some patients, despite appropriate treatment (Willemsen *et al*, 2010). On the contrary, most of the type B THD patients show no improvement in encephalopathy or motor skills when treated with levodopa (Willemsen *et al*, 2010; Furukawa & Kish, 2017). Furthermore, supplementing with L‐Dopa in those patients is often associated with side effects, such as L‐Dopa‐induced dyskinesias (Pons *et al*, 2013).

Therefore, the early diagnosis and identification of reliable THD biomarkers, as well as the establishment of new therapies, are an unmet medical need. However, poor overall understanding of disease mechanisms together with the heterogeneity within and between different forms and the low prevalence of the disease (Dong *et al*, 2020) has hampered the development of powerful appropriate and timely treatment.

Several mouse models of THD have been developed and used to evaluate treatment for motor correction; however, they fail to fully recapitulate the spectrum of pathological manifestations observed in patients (Kobayashi *et al*, 1995; Zhou *et al*, 1995; Althini *et al*, 2003; Tokuoka *et al*, 2011; Korner *et al*, 2015; Rose *et al*, 2015). A mouse model of THD (Korner *et al*, 2015) carrying Th p.Arg203His (equivalent to p. Arg233His in humans, the most recurrent mutation producing THD), shows decreased brain DA and low brain TH. Yet, these mice do not reflect patient heterogeneity in motor dysfunction, biochemical phenotype, and variability in response to L‐Dopa treatment (Willemsen *et al*, 2010; Fossbakk *et al*, 2014), which would imply that THD is an autosomal recessive heterogeneous disease, in which different stages of the catecholaminergic systems can modulate the human phenotype.

The currently used THD cellular model which includes rat PC12 cells expressing several human TH mutations does not resemble the cell type of the relevant disease nor the metabolic and functional characteristics of the patient (Hole *et al*, 2015). In a recent study, using a type B patient's post‐mortem brain tissue (TH p.Arg328Trp and p.Thr399Met), low levels of dopaminergic protein expression including TH, AADC, VMAT1, VMAT2, D1DR, and D2DR and an altered expression of GABAergic and glutamatergic proteins have been described (Tristán‐Noguero *et al*, 2016). However, the effect of TH mutations in dopaminergic neurons (DAn) or the main pathogenic mechanism underlying the different phenotypes between THD type A and type B remain unclear. Therefore, there is a strong need for human‐derived experimental models to explore the pathogenesis of THD and test new therapeutic strategies.

Here, we reprogrammed fibroblasts from THD patients with mild (type A; TH p.Arg233His) and severe (type B; TH p.Arg328Trp and p.Thr399Met) forms of the disease (hereinafter referred to as THDA and THDB, respectively). Induced pluripotent stem cells (iPSCs) were differentiated into patient‐specific DAn, which were compared in terms of morphology, DA metabolism, and treatment responsiveness, with DAn from healthy individuals and gene‐corrected isogenic controls. THD iPSC DAn displayed specific THD pathological features that matched patients' phenotypes, such as lower levels of DA metabolites and reduced TH expression. We also observed an altered expression level of the DA‐related genes compared to controls. Furthermore, THD iPSC DAn showed abnormal morphology, including reduced neurite length and neurite arborization defects. Finally, treatment of THD iPSC DAn with L‐Dopa rescued neuronal defects and disease phenotype in THD type A neurons, but not in those of type B patients. Interestingly, treatment with L‐Dopa at the stage of neuronal precursors could prevent the appearance of alterations in the DAn derived from THDB‐iPSC.

## Results

### Generation of THD‐specific iPSC lines

**Figure 1:**
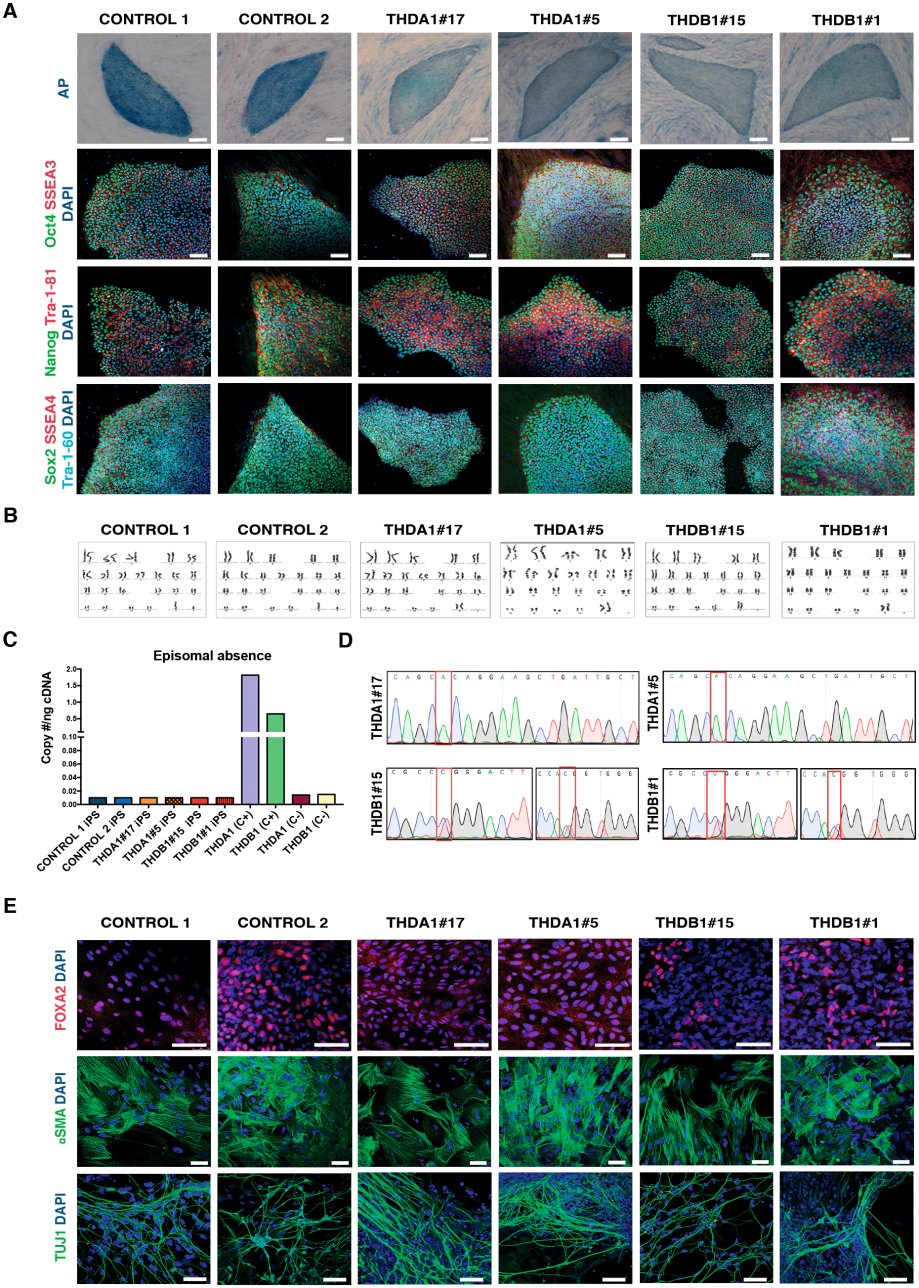
THD patient and healthy control iPSC lines show *bona fide* pluripotent stem cell features CONTROL 1, CONTROL 2, THDA1#17, THDA1#5, THDB1#15 and THDB1#1 iPSC stained for alkaline phosphatase (AP) activity and for the pluripotency‐associated markers OCT4, NANOG, SOX2 (green), SSEA3, TRA‐1‐81, SSEA4 (red), and TRA‐1‐60 (cyan).No karyotypic abnormalities were observed in all iPSCs at passage 15.Copy of gDNA (#/ng) of the episomal vector. Positive control (C+): THD iPSC line nucleofected with GFP, negative control (C−): fibroblasts of each THD patient.Direct sequencing of genomic DNA from THDA and THDB iPSCs identifying the c.698G>A and c.982C>T and c.1196C>T mutations, respectively.Immunofluorescence analyses of controls (CONTROL 1 and 2), THDA‐iPSC (THDA1#17 and THDA1#5), and THDB‐iPSC (THDB1#15 and THDB1#1) differentiated *in vitro* show the ability to generate cell derivatives of all three primary germ cell layers, including endoderm (stained for FOXA2, red), mesoderm (stained for α SMA, green), and ectoderm (stained for TUJ1, green). Data information: In (A) and (E) nuclei are counterstained with DAPI, shown in blue. Scale bars, 100 μm.

A total of seven iPSC lines from THD patients and healthy age‐matched controls, along with a gene‐edited counterpart, were used for the current study (see Table 1). Two clones of each cell line were thoroughly characterized and show a full reprogramming to pluripotency, as judged by colony morphology, alkaline phosphatase (AP) staining, expression of pluripotency markers (OCT4, NANOG, SOX2, SSEA3, SSEA4, TRA1‐81, and TRA1‐60), karyotype stability, and silencing of episomal vectors (Fig 1A–C). The presence of TH mutations was confirmed by sequencing (Fig 1D). Finally, all iPSC lines differentiated into cell types of the three embryonic germ layers as indicated by expression of specific markers, namely, FOXA2 (endoderm marker), αSMA (mesoderm marker), and TUJ1 (ectoderm marker) (Fig 1E). Therefore, iPSC generated from THD patients or from healthy individuals display *bona fide* pluripotent stem cell features and were indistinguishable in all tests performed, with the exception that THD‐iPSC lines carried the TH mutations.

**Table 1:** iPSC line information of the clones used in the study and demographics of unaffected individuals and THD patients.

	Clinical information												
	Patient ID	Cell line code	Cell ID used in this study	Status	Sex	Age at biopsy punch (years)	Onset age/Age at D(x) & treatment	Symptoms	Phenotype	Response to L‐Dopa	CSF markers (nmol/l)	Mutations	Previous related publications
Controls	CONTROL 1	FiPS Ctrl1‐ Ep6F‐5	CONTROL 1	Control	M	9	‐	‐	‐	‐	‐	‐	
	CONTROL 2	FiPS Ctrl2‐Ep6F‐8	CONTROL 2	Control	M	3	‐	‐	‐	‐	‐	‐	
Patients	THDA	THD FiPS A1 Ep6F‐5	THDA1#5	THDA	F	8	4 m/11 m	Tremor (++), hypokinesia (++), rigidity (+). Normal IQ (tested at 4.5 years)	A	Good	HVA: 158 (range: 344–906) HVA/HIAA: 0.64 (range: 1.5–3.5)	p.Arg233His/p. Arg233His	Ortez *et al* (2015), Kuseyri Hübschmann *et al* (2021) and Tristán‐Noguero *et al* (2021)
		THD FiPS A1 Ep6F‐17	THDA1#17	THDA									
		THD FiPS A1 Ep6F‐17 repaired	isoTHDA1#17	Isogenic control								p. Arg233His/p. Arg233His repaired	
	THDB	THD FiPS B1 Ep6F‐1	THDB1#1	THDB	F	15	5 m/3 y	Oculogyric crisis (++), tremor (+), hypokinesia (++), rigidity (++), autonomic dysfunction (+). Intellectual disability, No use of language	B	Slow improvement. Autonomous gait. Initially with dyskinesias after slow L‐Dopa increases	HVA: 15 (range: 304–658) HVA/HIAA: 0.05 (range: 1.5–3.5)	p. Arg328Trp /p.Thr399Met	Møller *et al* (2005), Ortez *et al* (2015) and Tristán‐Noguero *et al* (2016, 2021)
		THD FiPS B1 Ep6F‐15	THDB1#15	THDB									

D(x), diagnosis; F, feminine; HIAA, 5‐hydroxyindoleacetic acid; HVA, homovanillic acid; ID, identification; IQ, intelligence quotient; m, month; M, masculine; y, year.

**Figure EV1:**
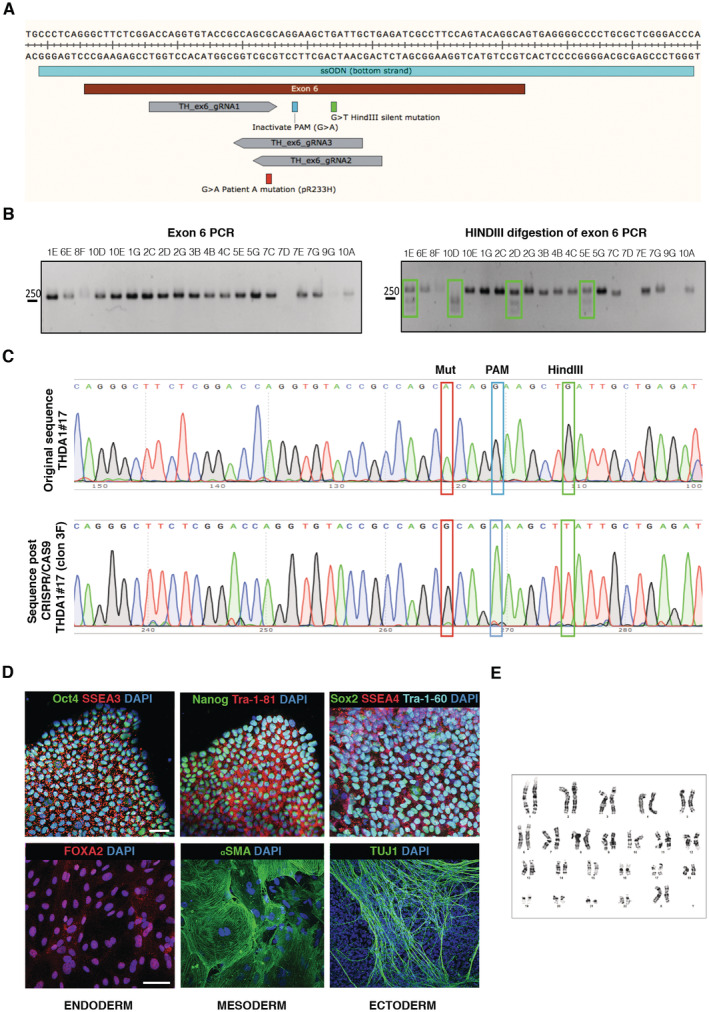
Isogenic control from p.Arg233His mutation carrier was generated and shows *bona fide* pluripotent stem cell features A region of the exon 6 with the ssODN, the gRNA, the PAM sequence, the mutation repaired, and the new restriction site for HindIII.PCR of exon 6 (left) and digestion of this PCR product with HindIII (right) (green boxes show digested PCR products).Sequence of a positive clone (3F) where the mutation was repaired, the sequence PAM is inactivated, and a new restriction site is introduced; all in homozygosis.Representative images of isoTHDA1#17 iPSC stained positive for the pluripotency‐associated markers OCT4, NANOG, Sox2, SSEA3, TRA‐1‐81, SSEA4, and Tra1‐60 and for the three primary germ cell layers, including endoderm (stained for FOXA2, red), mesoderm (stained for αSMA, green), and ectoderm (stained for TUJ1, green). Nuclei are counterstained with DAPI, shown in blue. Scale bars, 100 μm.Normal karyotype of isoTHDA1#17 iPSC at passage 25.

Additionally, we generated a gene‐corrected isogenic control iPSC line from the THDA patient carrying the Arg233His mutation (hereinafter referred to as isoTHDA1#17) using CRISPR/Cas9 (Fig EV1A–C). We focused on the mutant line THDA1#17 because it is homozygous for the most frequent mutation (Arg233His) found in THD patients that can cause a type A or type B phenotype depending on factors not yet fully understood (Willemsen *et al*, 2010; Fossbakk *et al*, 2014). Full characterization of the isoTHDA1#17 iPSC line revealed that CRISPR/Cas9‐editing did not affect its pluripotency, differentiation potential, or karyotype stability (Fig EV1D and E).

**Figure 2:**
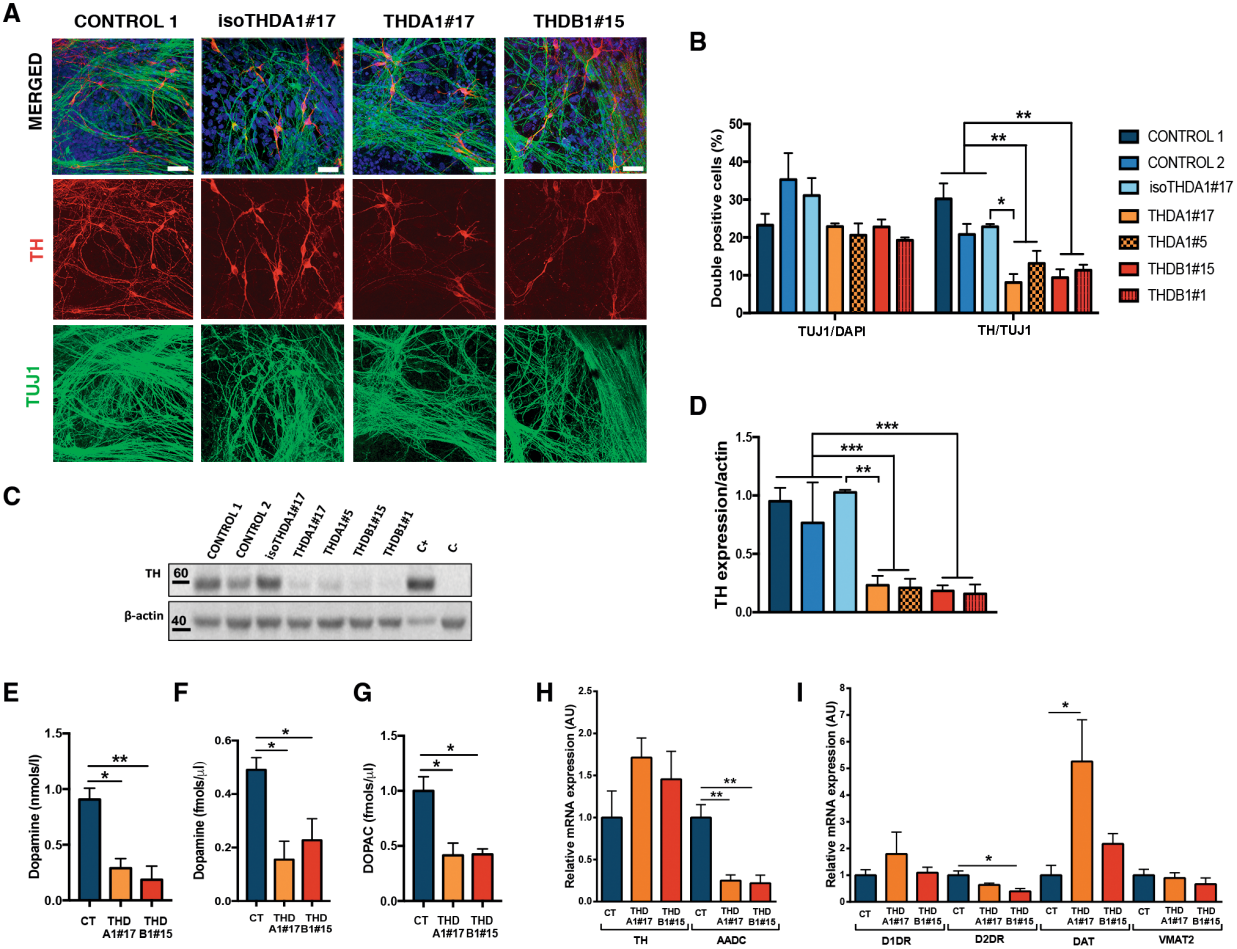
Dopaminergic neurons derived from THD‐iPSC recapitulate the disease phenotype Representative immunofluorescence (IF) images of CONTROL 1, isoTHDA1#17, THDA1#17, and THDB1#15 neuronal cultures (TH in red, TUJ1 in green, and DAPI in blue) at day 30 of differentiation.Quantification of TUJ1/DAPI and TH/TUJ1 ratios in all cell lines (CONTROL 1, CONTROL2, isoTHDA1#17, THDA1#17, THDA1#5, THDB1#15, and THDB1#1) (*n* = 3, experiments per iPSC line except for THDB1#1 clone that includes *n* = 2).Western blot analysis for determining TH expression in each cell line; positive control (C+): mesencephalon, negative control (C−): fibroblasts,Quantification of Western blot results (normalized by beta‐actin expression).ELISA quantification of intracellular dopamine levels (nmols/l) in cultures of control (CT), including CONTROL 1, CONTROL 2, isoTHDA1#17 lines, THDA1#17, and THDB1#15.HPLC quantification of dopamine levels (fmols/μl) in cultures of control (CT), including CONTROL 1, CONTROL 2, isoTHDA1#17 lines, THDA1#17, and THDB1#15.HPLC quantification of DOPAC levels (fmols/μl) in cultures of control (CT), including CONTROL 1, CONTROL 2, isoTHDA1#17 lines, THDA1#17, and THDB1#15. Data from isoTHDA1#17 are included as a pool with the Control.Relative mRNA expression of dopaminergic enzymes (*TH* and *AADC*) in CONTROL 1, CONTROL 2, THDA1#17 and THDB1#15.Relative mRNA expression of other DA genes expression (dopamine receptors, *DAT* and *VMAT2*). Data information: Scale bars, 20 μm (D–I, *n* = 3 experiments). Data are expressed as mean ± SEM. ANOVA or Kruskal–Wallis tests were performed for multiple comparisons. Unpaired two‐tailed Student's *t*‐test or Mann–Whitney U‐test was used for pairwise comparisons. ***P < 0.001; **P < 0.01; *P < 0.05. Source data are available online for this figure.

### Generation of THD‐specific DA neurons

**Figure EV2:**
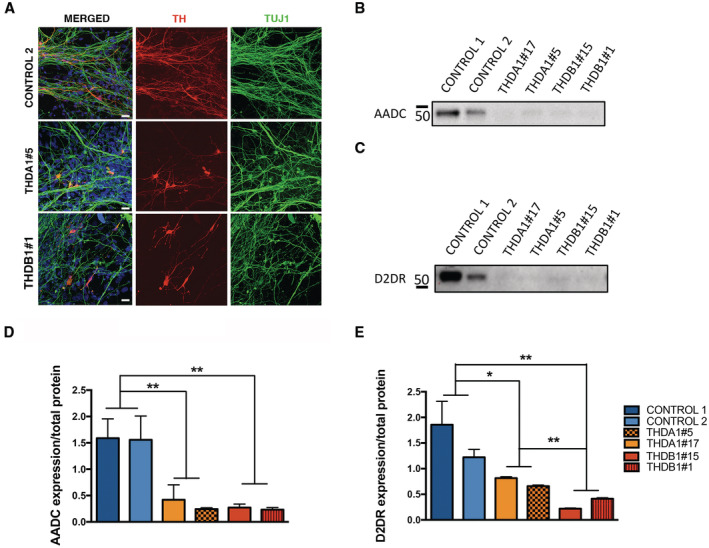
iPSC‐derived neurons from THDA1#5 and THDB1#1 clones recapitulate the reduced number of TH+ neurons ARepresentative immunofluorescence (IF) images of CONTROL 2, THDA1#5, and THDB1#1 iPSC neuronal cultures (TH in red, TUJ1 in green, and DAPI in blue) at day 30 of differentiation. Scale bars, 20 μm.B, CWestern blot of AADC (B) and D2DR (C) expression in CONTROL 1, CONTROL 2, THDA1#5, THDA1#17, THDB1#15, and THDB1#1 iPSCs neuronal cultures.D, EQuantification of Western blot results for AADC (D) and D2DR (E) normalized for total protein (D, *n* = 3 experiments; E, *n* = 2 experiments). Data are expressed as mean ± SEM. Unpaired two‐tailed Student's *t*‐test or Mann–Whitney U‐test was used for pairwise comparisons. **P < 0.01; *P < 0.05. Source data are available online for this figure.

For direct differentiation of iPSC toward DAn, we next subjected all iPSC lines to a 30‐day differentiation protocol using DA patterning factors and mouse PA6 stromal cell line co‐culture without using LMX1A induction (Sánchez‐Danés *et al*, 2012) (Appendix Fig S1A). As a technical control, we differentiated iPSCs from the same subjects into neural cultures not‐enriched‐in‐DAn using a different protocol that allows to generate mainly glutamatergic and GABAergic neurons through neuroepithelial progenitor cells (NPCs) (Chambers *et al*, 2009) (Appendix Fig S1B). We examined the expression of dopaminergic‐associated genes, such as *TH*, *AADC* (aromatic L‐amino acid decarboxylase), *D1DR and D2DR* (dopamine receptors 1 and 2), *DAT* (DA transporter), and *VMAT2* (vesicular monoamine transporter type 2) (Appendix Fig S1C) in iPSC‐derived neural cultures, and we found an overall enrichment of these genes only when using DA‐enriched protocol, indicating the successful generation of DAn with *bona fide* dopaminergic molecular characteristics (Hwang *et al*, 2010). Moreover, we found that DAn generated from control and THD iPSCs were mature as judged by immunofluorescence analysis against neuron‐specific class III‐b‐tubulin (TUJ1) and tyrosine hydroxylase (TH) (Figs 2A and EV2A).

In addition, we found significant increased expression of other DA‐related markers such as *GIRK*, *PITX3*, *LMX1A*, *FOXA2*, *NURR1*, and *EN1* in our cell cultures as compared to iPSC cultures, further confirming the dopaminergic identity of the neurons generated (Appendix Fig S2).

We then investigated whether the presence of THD mutations affects TH expression levels by counting the number of TH‐expressing cells in cultures enriched for DAn. After detailed analysis, we found less TH‐immunoreactive cells (~5 to 10% of all TUJ1+ cells *P* < 0.01) in THD neuronal cultures compared with control and isogenic iPSC‐derived cells (~25 to 30% of all TUJ1+ cells). However, no differences were observed in the number of TUJ1+ neurons generated by all iPSC lines (Figs 2A and B, and EV2A), suggesting that the TH mutations may specifically affect DAn production. To further confirm these results, we tested the TH protein expression levels and found significantly lower TH expression levels in both THDA and THDB neuronal cultures as compared to control ones (*P* < 0.001; Fig 2C and D).

We subsequently attempted to examine the functionality of DAn differentiated from THD patient‐specific iPSC by determining intracellular and released dopamine (DA) and its 3,4‐dihydroxyphenylacetic acid (DOPAC) metabolite in cultured THD and control neurons. In all patient‐derived cultures, we found low intracellular DA levels compared to those measured in the control and isoTHD neuronal cultures (*P* < 0.05 for THDA and *P* < 0.01 for THDB; Fig 2E). Moreover, levels of extracellular DA and its DOPAC metabolite were significantly lower in media collected from THDA and THDB neuronal cultures, as compared with the concentrations measured from control and isoTHD cultures (*P* < 0.05; Fig 2F and G), thus confirming that TH mutations in those neurons significantly reduced enzyme activity affecting both production and release of DA. Interestingly, the use of the isoTHDA1#17 neuronal cultures restored the previous phenotype, confirming the latter's dependence on the mutation (Fig 2A–G).

Finally, we investigated whether lower TH expression levels in THD dopaminergic cultures could impact the expression of other DA‐related genes. We found reduced mRNA expression of *AADC* in both THD cultures (*P* < 0.01; Fig 2H) and lower *D2DR* expression levels in THDB cultures (*P* < 0.05; Fig 2I), whereas we detected higher *DAT* expression levels in THDA neuronal cultures (*P* < 0.05; Fig 2I) and no differences in *VMAT2* between groups (Fig 2I). We further evaluated the expression levels of AADC and D2DR at protein level and found a significant reduction in THD cultures (AADC: *P* < 0.01; Fig EV2B and D; D2DR: *P* < 0.05 for THDA and *P* < 0.01 for THDB; Fig EV2C and E), confirming the impairment on DAn production in both THD neuronal cultures. Interestingly, although we found reduced TH expression at protein levels (Fig 2C and D), we did not detect differences at mRNA levels between control and THD neuronal cultures (Fig 2H) as it has been previously described (Korner *et al*, 2015).

Taken together, these data reveal that our new iPSC‐based THD model recapitulates most of the phenotypes observed in patients, allowing for the study of THD pathogenesis in diseased brain cells.

### Morphological alterations of THD‐iPSC‐derived DA neurons

**Figure 3:**
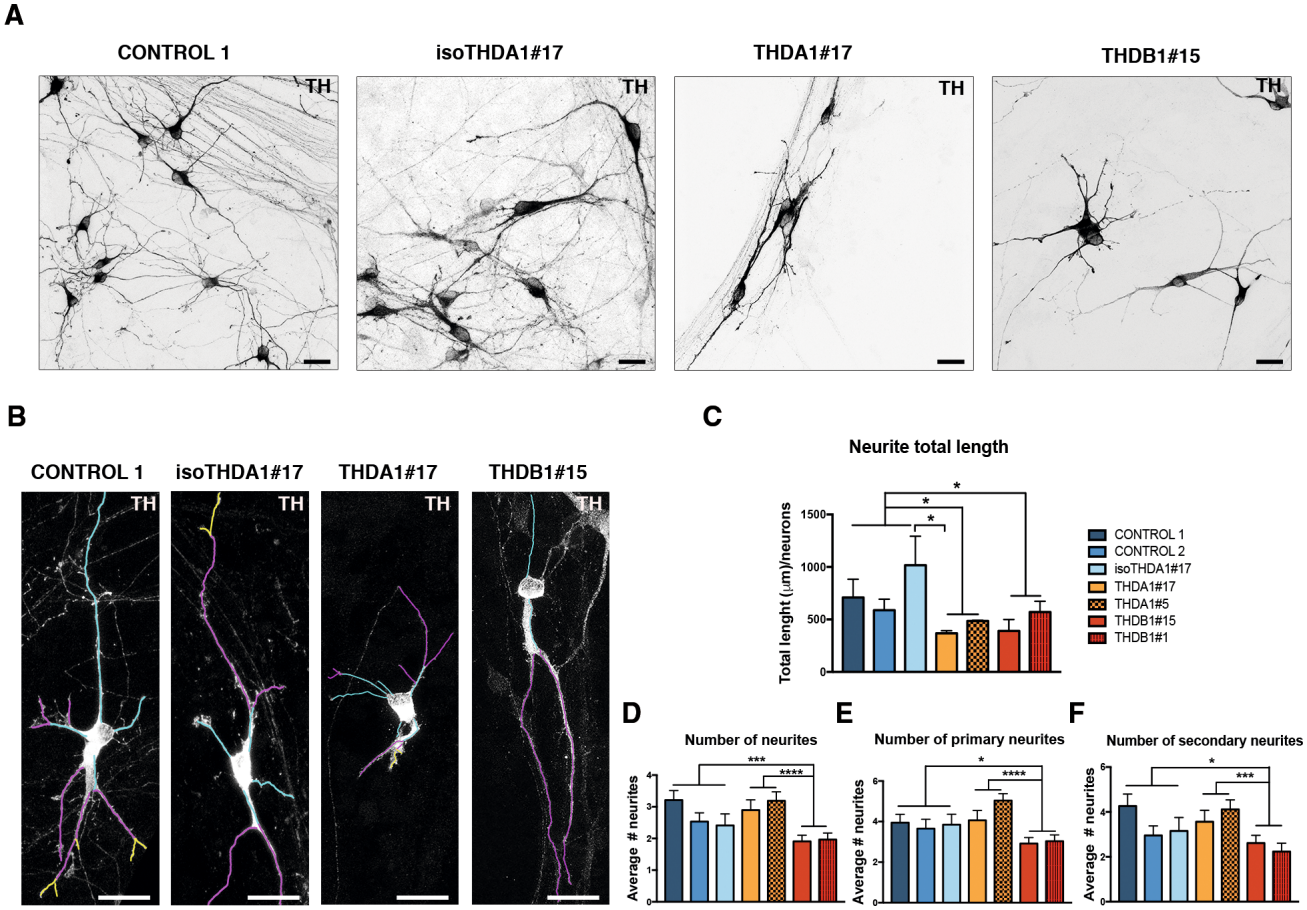
THD iPSC‐derived TH+ neurons are morphologically abnormal Immunofluorescence (IF) images of neuronal cultures (TH in black) of CONTROL 1, isoTHDA1#17, THDA1#17, and THDB1#15 cell lines.Representative images of the tracing analysis (TH in white, primary neurites in blue, secondary in magenta, and tertiary in yellow) in the same cell lines.Quantification of the neurite total length in all cell lines (CONTROL 1, CONTROL2, isoTHDA1#17, THDA1#17, THDA1#5, THDB1#15, and THDB1#1).Number of neurites.Number of primary neurites.Number of secondary neurites. Data information: Scale bars, 20 μm (C–F, *n* = 3, experiments per iPSC line except for isoTHDA1#17 that includes *n* = 2; at least 10 neurons counted per experiment). Data are expressed as mean ± SEM. ANOVA or Kruskal–Wallis tests were performed for multiple comparisons. Unpaired two‐tailed Student's *t*‐test or Mann–Whitney U‐test was used for pairwise comparisons. ****P < 0.0001; ***P < 0.001; *P < 0.05. Source data are available online for this figure.

**Figure EV3:**
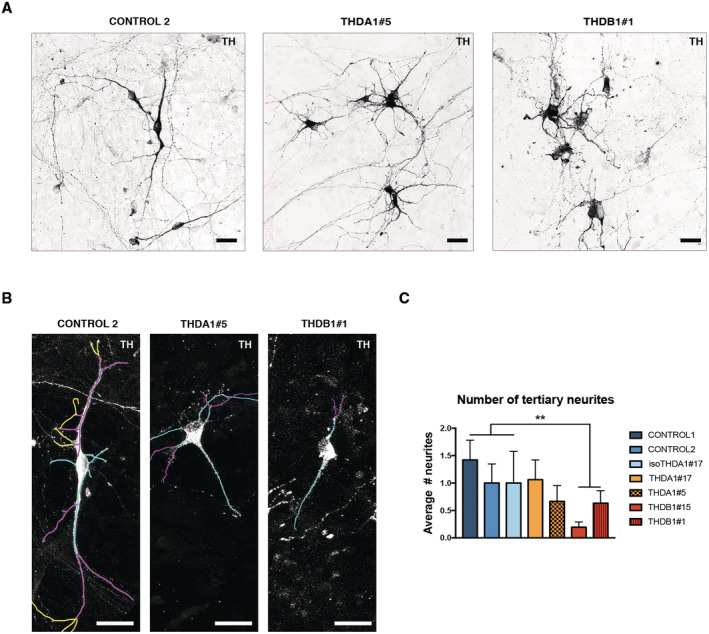
iPSC‐derived neurons from THDA1#5 and THDB1#1 clones show abnormal morphology Immunofluorescence (IF) images of neuronal cultures (TH in black) of the same lines.Images of the tracing analysis (TH in white, primary neurites in blue, secondary in magenta, and tertiary in yellow) in CONTROL 2, THDA1#5, and THDB1#1 neuronal cultures.Number of TH tertiary neurites in all cell lines (CONTROL 1, CONTROL 2, isoTHDA1#17, THDA1#17, THDA1#5, THDB1#15, and THDB1#1). Data information: Scale bars, 20 μm (*n* = 3 experiments, at least 10 neurons counted per experiment). Data are expressed as mean ± SEM. ANOVA test was used for multiple comparisons. **P < 0.01. Source data are available online for this figure.

After establishing DAn‐enriched cultures from both control and THD patient‐specific iPSC, we next evaluated whether there were any differences in neuronal phenotype between DAn derived from controls and those from patients' iPSC lines. We noted that THD iPSC‐derived DAn developed a range of abnormal morphologies over 30 days in culture that were not observed in control iPSC‐derived DAn (Figs 3A and EV3A). Specifically, while DAn differentiated from control iPSC showed long neurites with complex dendritic arborization, DAn from THD‐iPSC had fewer and simpler processes (Figs 3B and EV3B). To rule out any subjectivity in attributing the observed differences between these cultures, we directly measured the number and length of neurites. To this end, using high‐powered confocal images, we randomly picked single TH‐stained neurons from the THD patient‐specific and control iPSC lines (Figs 3B and EV3B; see also Chu *et al*, 2009). These analyses confirmed that in THDA and THDB, the iPSC‐derived DAn neurite length was significantly shorter as compared to control ones (*P* < 0.05; Figs 3B and C, and EV3C). Interestingly, the causative role of patients' mutation was further confirmed in DAn differentiated from gene‐corrected iPSC, as the isoTHDA1#17 iPSC had a longer total neurite length than THDA1#17 (*P* < 0.05; Fig 3B and C).

Moreover, THDB DAn showed fewer neurites when compared to controls and THDA DAn (*P* < 0.001 and < 0.0001, respectively; Figs 3B and D, and EV3B). Specifically, we observed fewer primary, secondary, and tertiary neurites in DAn derived from THDB‐iPSC compared to neurons derived from controls (*P* < 0.05 for primary and secondary neurites and *P* < 0.01 for tertiary neurites) or compared with THDA‐iPSC (*P* < 0.0001 for primary neurites and *P* < 0.001 for secondary neurites; Figs 3B, E and F, and EV3B and C).

Overall, our data suggest that although THD‐specific iPSCs differentiated normally into DAn, they unveil morphological alterations upon 30 days in culture.

### Treatment with L‐Dopa and carbidopa rescues THD‐specific phenotypes in THDA iPSCs‐derived DA neurons

**Figure 4:**
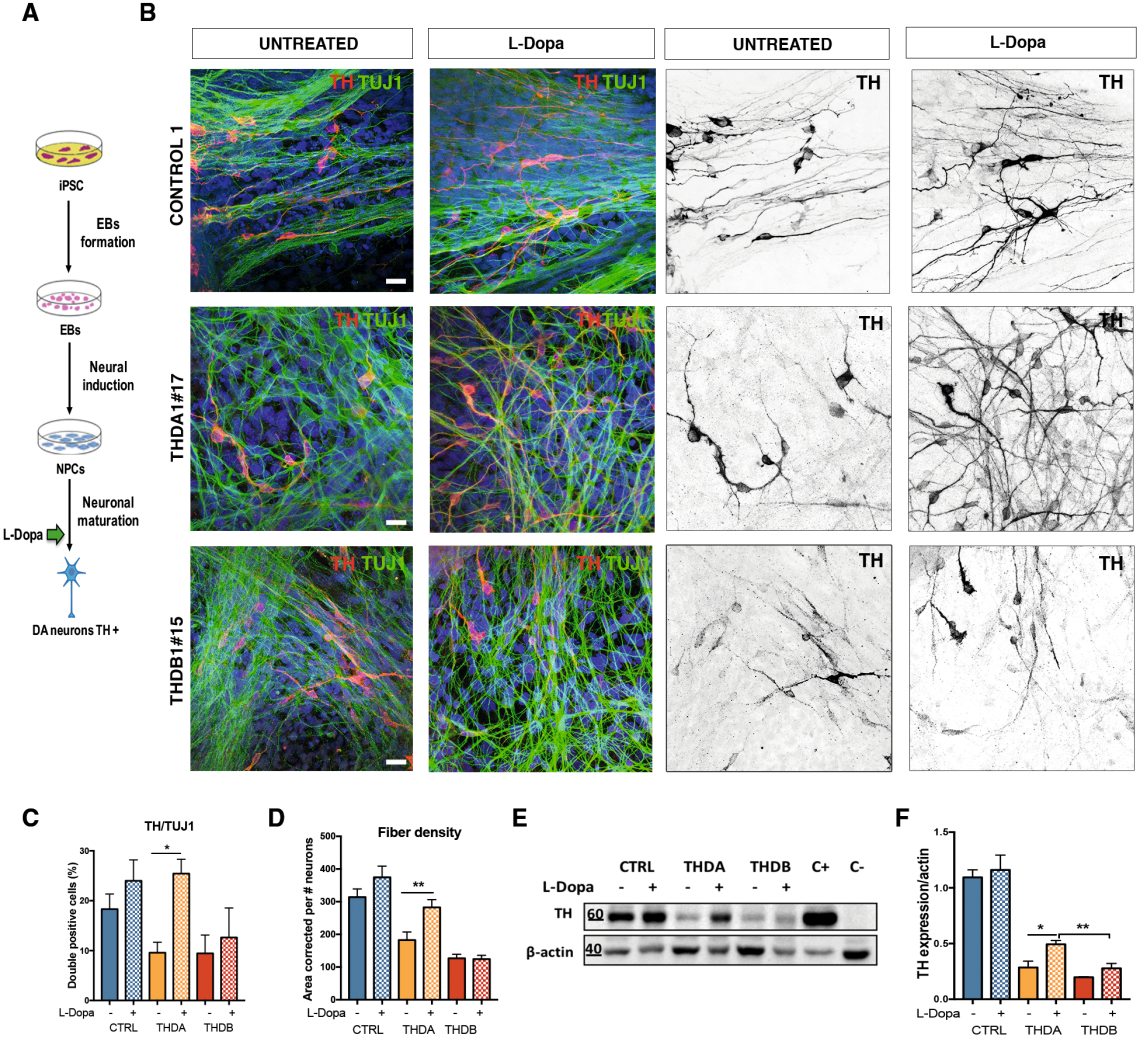
L‐Dopa and Carbidopa treatment rescues the described phenotype only in THDA Schematic representation of treatment administration.Immunofluorescence (IF) images of CONTROL 1, THDA1#17, and THDB1#15 neuronal cultures (TH in red, TUJ1 in green and DAPI in blue) and in black and white to show TH staining.Quantification of the TH/TUJ1 ratio.Quantification of the fiber density.Western blot of TH expression after L‐Dopa treatment; positive control (C+): mesencephalon, negative control (C−): fibroblasts in CONTROL 1, THDA1#17 and THDB1#15.Quantification of Western blot results (normalized by beta‐actin expression). Data information: Scale bars, 20 μm (C, D, F, *n* = 3 experiments; in (D) at least 20 images analyzed per iPSC line). Data are expressed as mean ± SEM. Unpaired two‐tailed Student's *t*‐test or Mann–Whitney U‐test was used for pairwise comparisons. **P < 0.01; *P < 0.05. Source data are available online for this figure.

We next sought to obtain proof of principle that L‐Dopa treatment of THD patient‐specific iPSC‐derived DAn could rescue the morphological changes described above. For this purpose, we used L‐Dopa together with Carbidopa (hereinafter referred to as L‐Dopa) because in this way, it is administered to patients in order to inhibit the AADC enzyme and avoid the peripheral metabolism of L‐Dopa (Willemsen *et al*, 2010; Furukawa & Kish, 2017). At the end of the 30‐day differentiation protocol, which coincides with the endpoint treatment (Burbulla *et al*, 2017), the number of DAn was assessed by immunofluorescence for TH expression and unbiased counting (Fig 4A). Treated THDA neuronal cultures showed a higher number of TH+ neurons as judged by the TH/TUJ1 ratio (*P* < 0.05; Fig 4B and C). Fiber density was also increased in treated DAn derived from THDA iPSC (*P* < 0.01; Fig 4B and D). Moreover, we observed increased TH protein expression levels in treated THDA neuronal cultures (*P* < 0.05; Fig 4E and F). However, none of the previously described THD phenotypes was rescued in the THDB neuronal cultures (Fig 4B–F).

**Figure EV4:**
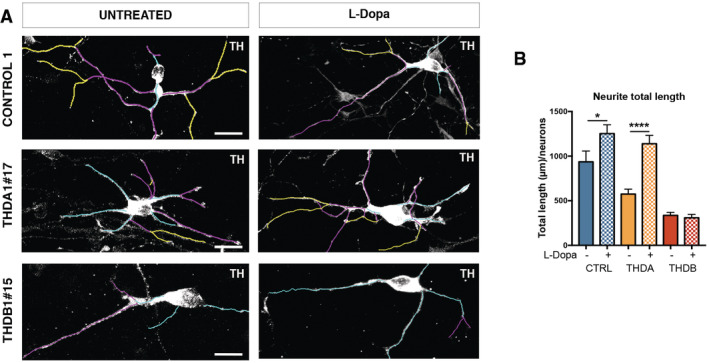
L‐Dopa and Carbidopa treatment rescue neurite total length in THDA Representative images of the tracing analysis (TH in white, primary neurites in blue, secondary in magenta and tertiary in yellow) of CONTROL 1, THDA1#17, and THDB1#15 lines.Quantification of the total neurite length in CONTROL 1, THDA1#17, and THDB1#15 untreated and treated cultures. Data information: Scale bars, 20 μm (*n* = 3, experiments per iPSC line; at least 10 neurons counted per experiment). Data are expressed as mean ± SEM. Unpaired two‐tailed Student's *t*‐test or Mann–Whitney U‐test was used for pairwise comparisons. ****P < 0.0001; *P < 0.05. Source data are available online for this figure.

Neurite tracing analysis of the treated control and THDA DAn revealed longer neurites (*P* < 0.05 and < 0.0001, respectively). Conversely, treatment with L‐Dopa did not rescue the abnormal arborization observed in THDB‐iPSC derived DAn (Fig EV4A and B).

**Figure 5:**
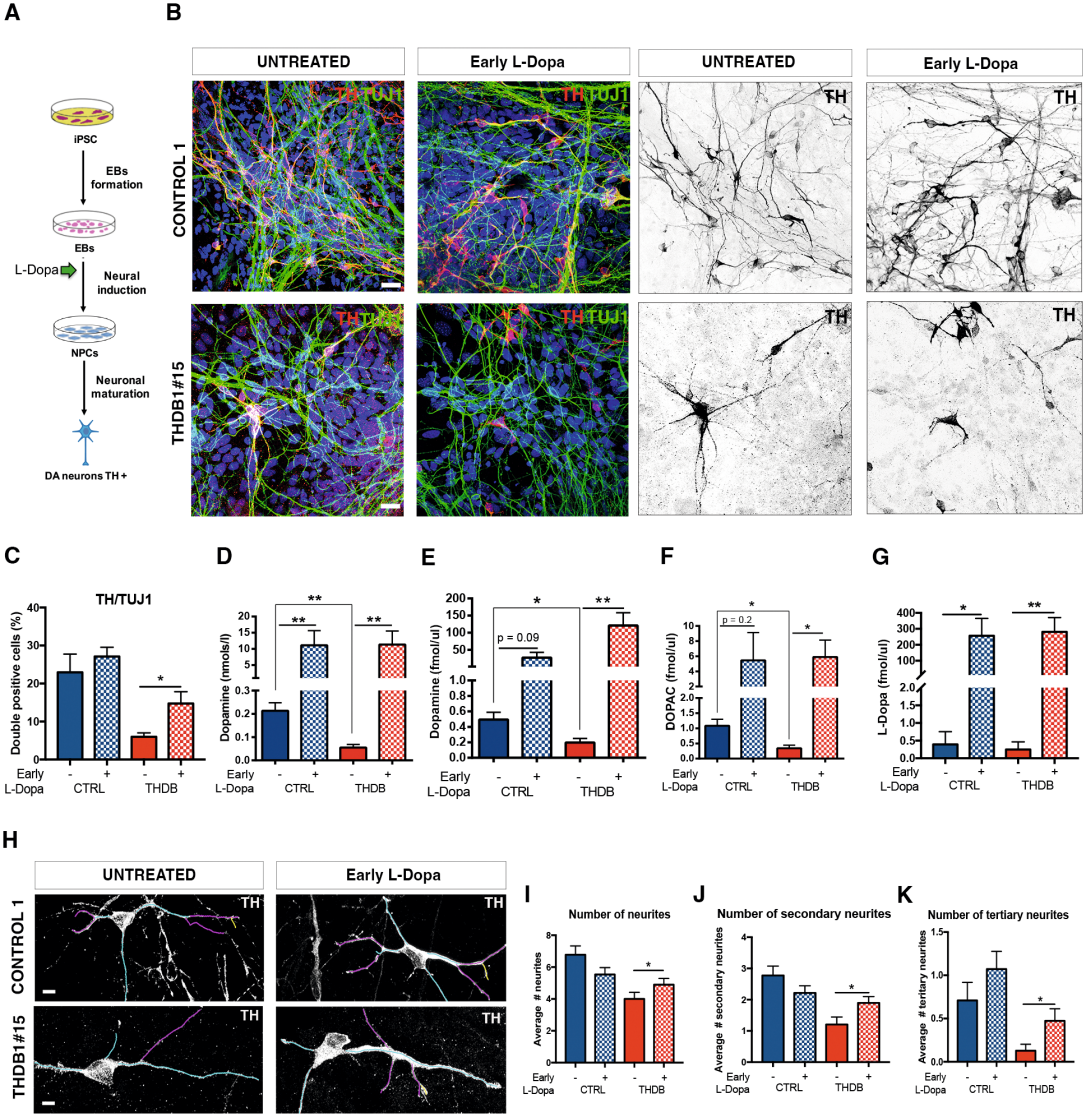
Early L‐Dopa treatment prevents deficits in THDB Schematic representation of treatment administration.Immunofluorescence (IF) images of CONTROL 1 and THDB1#15 neuronal cultures (TH in red, TUJ1 in green and DAPI in blue) and in black and white to show TH staining.Quantification of TH/TUJ1 ratio in the previous lines.ELISA quantification of intracellular dopamine levels (nmols/l) in the CONTROL 1 and THDB1#15 lines before and after treatment.HPLC quantification of dopamine levels (fmols/μl).HPLC quantification of DOPAC levels (fmols/μl).HPLC quantification of L‐Dopa levels (fmols/μl) in CONTROL 1 and THDB1#15 lines.Representative images of the tracing analysis (TH in white, primary neurites in blue, secondary in magenta and tertiary in yellow) of CONTROL 1 and THDB1#15 lines.Number of neurites.Number of secondary neurites.Number of tertiary neurites. Data information: Scale bars, 20 μm (C–G, I–K, *n* = 3, experiments per iPSC line except for panel (E), (F) in which CONTROL 1 treated with L‐Dopa includes *n* = 2; in (I–K) at least 10 neurons counted per experiment). Data are expressed as mean ± SEM. Unpaired two‐tailed Student's *t*‐test or Mann–Whitney U‐test was used for pairwise comparisons. **P < 0.01; *P < 0.05. Source data are available online for this figure.

**Figure EV5:**
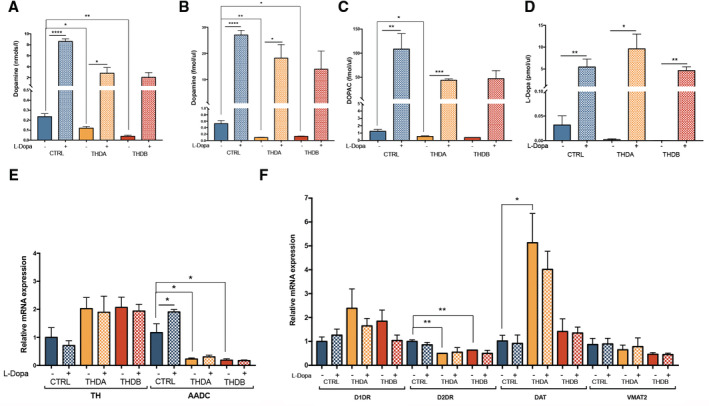
L‐Dopa and Carbidopa treatment partially rescues THDA phenotype ELISA quantification of intracellular dopamine levels (nmols/l) in CONTROL 1, THDA1#17 and THDB1#15 lines before and after treatment.HPLC quantification of dopamine levels (fmols/μl).HPLC quantification of DOPAC levels (fmols/μl).HPLC quantification of L‐Dopa levels (fmols/μl).Relative mRNA expression of DA enzymes (*TH* and *AADC*).Dopamine receptors (*D1DR* and *D2DR*), *DAT*, and *VMAT2* mRNA expression levels relative to Neural Specific Enolase (*NSE*) in CONTROL 1, THDA1#17, and THDB1#15. Data information: *n* = 3, experiments per iPSC line except for L‐Dopa treated THDA1#17 and THDB1#15 in panel (A); untreated THDB1#15 in panel (B) and (C); untreated CONTROL 1 in panel (C) that includes *n* = 2 experiments. Data are expressed as mean ± SEM. Unpaired two‐tailed Student's *t*‐test or Mann–Whitney U‐test was used for pairwise comparisons. ****P < 0.0001; ***P < 0.001; **P < 0.01; *P < 0.05. Source data are available online for this figure.

Next, we tested whether L‐Dopa treatment could also impact in the levels of DA metabolites in the neuronal cultures and found an increase in intracellular DA levels in control and THDA neuronal cultures, but not in those derived from THDB‐specific iPSC (*P* < 0.0001 and < 0.05, respectively; Fig EV5A). Furthermore, we found a higher level of extracellular DA (*P* < 0.0001 and < 0.05, respectively) and DOPAC (*P* < 0.01 and < 0.001, respectively) in both control and THDA neuronal cultures (Fig EV5B and C). In contrast, none of the previously mentioned DA metabolites was detected at higher level in the treated THDB DAn. Finally, as expected, we observed more L‐Dopa in the supernatant media collected from control (*P* < 0.01), THDA (*P* < 0.05), and THDB (*P* < 0.01) neuronal cultures (Fig EV5D). At the mRNA level, we found increased *AADC* expression levels in control neuronal cultures (*P* < 0.05; Fig EV5E) treated with L‐Dopa, whereas no statistical differences were observed in other DA‐related gene expression, such as *D1DR*, *D2DR*, *DAT*, and *VMAT2* (Fig EV5F).

### Early treatment with L‐Dopa prevents THDB iPSC‐derived DAn abnormalities

Since DA is one of the first neurotransmitters expressed during brain development, alterations in its signaling could affect the differentiation of specific subpopulations of neurons (Money & Stanwood, 2013). To directly evaluate the hypothesis that crucial events early in brain development could be responsible for the lack of response to treatment in type B (Willemsen *et al*, 2010), we established neuronal cultures from THDB‐iPSC and assessed the effect of an L‐Dopa early treatment. Specifically, L‐Dopa and Carbidopa were added to the cultures starting from day 6, at the neuronal precursor stage, and maintained until day 30 (Fig 5A), at the time when THD phenotypes in THDB Dan were observed. Of note, early treatment with L‐Dopa prevented the appearance of alterations in THDB iPSC Dan, as demonstrated by the increase in the number of TH‐positive cells (*P* < 0.05; Fig 5B and C) and by restoring intracellular DA levels (*P* < 0.05; Fig 5D) as well as extracellular levels of DA, DOPAC, and L‐Dopa (*P* < 0.05; Fig 5E–G). Moreover, early treatment with L‐Dopa reduced the number of TH‐positive cells with an abnormal morphology (Fig 5H–K). Importantly, these findings suggest the existence of a critical developmental window when new therapies could be successfully employed in THD.

## Discussion

In this study, we describe the first iPSC‐based model of THD with non‐integrative episomal vectors of two THD patients, one with a mild phenotype (THDA) and another with a severe phenotype (THDB). In addition, by CRISPR/Cas9 gene editing technique, we repaired the THDA iPSC line. Similar to previous studies modeling other dopamine neurotransmitter defects, we were able to obtain DAn from both control and THD patients independently of the disease status (Ishikawa *et al*, 2016; Rossignoli *et al*, 2021).

Here, we show that our iPSC‐based THD model recapitulates the disease phenotype associated with the disease. As expected, THD patient‐derived DAn showed a reduction in DA metabolites when compared to controls. The same has been observed in mouse models carrying mutations in the *TH* gene (Kobayashi *et al*, 1995; Zhou *et al*, 1995; Althini *et al*, 2003; Tokuoka *et al*, 2011; Korner *et al*, 2015; Rose *et al*, 2015). We also observed a reduction in TH protein expression levels in both THDA‐ and THDB‐derived DAn compared to controls. This effect was previously described in a *post‐mortem* brain study of a patient with type B THD (Tristán‐Noguero *et al*, 2016), in a PC12 cell line in which *TH* mutations were exogenously expressed (Hole *et al*, 2015) and in THD mouse models (Kobayashi *et al*, 1995; Korner *et al*, 2015). In this study, we also analyzed the expression of several DA genes. We observed lower *AADC* mRNA levels in both types of THD cultures and a reduction in *D2DR* mRNA expression only in THDB‐derived Dan but not in THDA. These findings are in line with those of previous literature: a *post‐mortem* brain study of a patient with type B THD, in which reduced levels of AADC and D2DR proteins were observed (Tristán‐Noguero *et al*, 2016). Moreover, a mouse model for type A THD did not show lower expression of DA receptors (Rose *et al*, 2015). It is generally accepted that in the presence of lower levels of DA, such as upon DAn denervation, an upregulation of dopamine receptors occurs (Turjanski *et al*, 1997). In our study, we found lower levels of *D2DR* in THDB, but not in THDA. We hypothesize that low levels of DA could regulate presynaptic dopaminergic receptor expression at an early stage of development. Interestingly, the differences found among both receptors could cause an imbalance between direct and indirect pathways (Zeiss, 2005) and produce Parkinsonian symptoms, which are usually worse in Type B patients (Willemsen *et al*, 2010; Iravani *et al*, 2012). THDA‐iPSC‐derived neurons expressed higher mRNA levels of *DAT*, compared to controls. This effect could be an adaptive behavior to utilize more efficiently the DA produced in *TH*‐defective neurons by increasing the uptake of DA. We did not observe lower *TH* mRNA expression in THD DAn when compared to controls. This was somehow expected since these mutations are missense mutations and therefore are known to alter TH expression only at the level of protein. Similar results were described in a THD mouse model (Korner *et al*, 2015) where new‐born, juvenile, and adult THD mice presented higher *TH* mRNA levels when compared to wild type littermates. Importantly, this new human iPSC‐based model generated from THD patients' fibroblasts recapitulates some of the phenotypic aspects of human disease *in vitro*.

The most significant contribution of our work is the description of a new neuronal phenotype as we observed fewer and simpler processes in both THD‐derived DAn. This was also observed in THD mouse models, (Tokuoka *et al*, 2011; Korner *et al*, 2015; Rose *et al*, 2015), where lower TH signal in fibers, dendrites, and projections was reported. We observed shorter neurites in both THD neurons. which could be due to the lack of DA leading to a defect in DAn development (Li *et al*, 2015) and abnormal arborization in THDB neurons. DA is an important neurotrophic factor, which could potentiate a more complex neuronal morphology (Lieberman *et al*, 2018) to integrate synaptic information and promote neuronal plasticity (Arikkath, 2012). Most type A patients show preserved cognitive and learning skills, which may be related to undamaged dendritic trees. In contrast, an important proportion of Type B patients have moderate‐to‐severe intellectual disability (Willemsen *et al*, 2010; Ortez *et al*, 2015). In this study, we found an abnormal neuronal dendritic tree in THDB DAn that could be explained by lower *D2DR* mRNA expression levels as this receptor has been described as a modulator of neuronal morphology in the frontal cortex and striatum (Money & Stanwood, 2013; Li *et al*, 2015; Lieberman *et al*, 2018). These morphological observations are important in clarifying the different clinical phenotypes of type A and B. Importantly, CRISPR/Cas9 repair of the p.Arg233His TH point mutation abolished THDA deficit, restoring both the number of TH+ neurons and the TH protein levels to those observed in controls. An improvement in neuronal morphology was also observed. Thus, the genome modification data confirm the robust consistency of mutation‐related changes in THDA neuronal cultures.

It has been described that while type A phenotype patients respond well to L‐Dopa treatment (Willemsen *et al*, 2010), patients with type B phenotype respond poorly, have more severe symptoms (Iravani *et al*, 2012), and a higher prevalence of L‐Dopa‐induced dyskinesias (Pons *et al*, 2013).

We tested L‐Dopa treatment in neuronal cultures derived from a control and both patients. As expected, L‐Dopa treatment fully rescued the neuronal phenotypes in THDA‐derived neuronal cultures, but not in THDB. The higher number of TH‐immunoreactive cells observed in treated THDA cell culture has also been described in diverse animal models of Parkinson's disease (PD), where they show that either 6‐OHDA or MPTP‐lesioned animals treated with L‐Dopa present more TH+ neurons, thus refusing the toxic effect of L‐Dopa in the nigrostriatal population (Datla *et al*, 2001; Darmopil *et al*, 2008; DiCaudo *et al*, 2012). Higher TH protein expression levels could be due to a more stable TH enzyme due to its binding to catabolized L‐Dopa or DA. In relation to this, previous *in vitro* experiments indicated that the specific *TH* mutation p.Arg233His could be affecting TH thermal stability (Fossbakk *et al*, 2014). The improved fiber density and neurite length only observed in THDA cultures can be explained by the effect of DA signaling through D2DR, while the lack of response of THDB neurons to L‐Dopa could be due to lower basal levels of D2DR in THDB DAn. THDA neuronal cultures showed higher levels of DA metabolites after the L‐Dopa treatment; surely, the added L‐Dopa is catabolized, increasing the concentration of DA and its metabolites. Therefore, importantly, the iPSC‐based THD model described here mimics not only the phenotype observed in THD patients but also the response to the treatment.

L‐Dopa treatment was not able to rescue the deficits observed in THDB neurons, suggesting that crucial pathological events in THDB cells could occur early in neural development. To test this hypothesis, we administered L‐Dopa at an early stage and tested the response of THDB DAn. We observed that both the number and complex arborization of DAn increased. The first effect is already described in PD animal models treated with L‐Dopa (Datla *et al*, 2001; Darmopil *et al*, 2008; DiCaudo *et al*, 2012), and the second one is observed in neurons of the prefrontal cortex treated with DA (Li *et al*, 2015), and we hypothesize that the same could happen in the dopaminergic neuron population. The disease phenotype was also partially rescued with early L‐Dopa treatment as levels of DA metabolite were higher in THDB, indicating that the added L‐Dopa is now metabolized, when supplied during neuronal induction.

Overall, these results suggest that early treatment could prevent the alteration of DAn. However, translating it into clinics would imply that this treatment should be given prenatally, which is not common since most diseases are not diagnosed until birth. This is the case with most inborn errors of metabolism, such as THD. However, early treatment intervention has already shown promising results in guanosine triphosphate cyclohydrolase I (GTPCH, another enzyme involved in DA synthesis) deficiency (Brüggemann *et al*, 2012). Therefore, both the early diagnosis and early treatment of THD may greatly improve patient outcome.

It should be noted that our results were obtained on the basis of samples derived from only two patients; thus, more patients are needed to further validate our findings. Moreover, when working with stem cells, it should be kept in mind that variability between established lines may be found, but all recommended strategies to reduce the risk of cell variability have been implemented in the present study (Escribá *et al*, 2021).

To sum up, the iPSC‐based THD model described here is the first to recapitulate specific disease phenotypes and treatment response in both type A and B phenotypes of THD. This model will help us better understand the new molecular mechanisms of this disorder, and it is a valuable platform for testing different *in vitro* therapeutic approaches for the management of all patients with THD.

## Materials and Methods

### Description of THD patients

Studies were approved by the authors' Institutional Review Board, and in addition to WMA Declaration of Helsinki, the experiments also conformed the Department of Health and Human Services Belmont Report. Patients were encoded to protect their confidentiality and written informed consent was obtained. The generation of human iPS cells was done following a protocol approved by the Spanish competent authorities (Commission on Guarantees concerning the Donation and Use of Human Tissues and Cells of the Carlos III Health Institute). Control and THD‐specific iPSCs were generated by the P‐CMR[C] Stem Cell Bank. The subjects who participated in the study were patients attending the Neurometabolic Unit of Sant Joan de Déu Hospital (Barcelona, Spain) and the Neuropediatrics Department at the University Hospital Virgen de la Arrixaca (Murcia, Spain). One of the two THD patients has a type A phenotype (homozygous for the TH mutation p.Arg233His), while the other one has a type B phenotype (compound heterozygous for the TH mutations p.Arg328Trp and p.Thr399Met). Detailed clinical information for both patients is provided in Table 1.

### Generation and characterization of iPSC lines

Skin fibroblasts were reprogrammed to iPSCs using non‐integrative episomal vectors expressing Oct4, Sox2, Klf4, c‐myc, p53shRNA, and Lin28, as previously described (Kuebler *et al*, 2017) to generate 2–4 independent iPSCs clones per individual. THD patient‐specific and control iPSCs were maintained in mTeSR™ medium (STEMCELL technologies) and passaged once a week with EDTA onto Matrigel‐coated (Cultek) plates (Life technologies). The absence of episomal expression and endogenous pluripotency markers was evaluated as previously reported (Kuebler *et al*, 2017). *In vitro* differentiation toward the endoderm, mesoderm, and neuroectoderm was performed essentially as described (Martí *et al*, 2013). The iPSCs generated and characterized in this study were registered and deposited with the Spanish Bank of Stem Cells. Information on iPSC lines is provided in Table 1.

### Generation of CRISPR/Cas9 plasmids and gene edition in iPSC

For correcting the *R233H* mutation, THDA1#17 mutant iPSCs were edited using CRISPR/Cas9. Three CRISPR/Cas9 gRNAs targeting the p.Arg233His mutation site in the *TH* gene were designed together with an ssODN (SIGMA) carrying the wild‐type allele and two additional synonymous mutations to eliminate the PAM sequence and to introduce a *de novo* HindIII to help with the molecular screening (GGGTCCCGAGCGCAGGGGCCCCTCACTGCCTGTACTGGAAGGCGATCTCAGCAATAAGCTTTCTGCGCTGGCGGTACACCTGGTCCGAGAAGCCCTGAGGG). The T7 endonuclease I assay (New England Biolabs) was performed to assess which gRNA had the highest cleavage efficiency. The best performing gRNA (TH_ex6_gRNA1: ACCAGGTGTACCGCCAGCAC) was kindly provided by Synthego.

The selected iPSC line (THDA1#17) was seeded at a density of 300,000 cells per well of a six‐well plate. The next day, iPSCs were nucleofected with a preassembled gRNA and Cas9 protein RNP complex (ThermoFisher Scientific) using the 4D AMAXA nucleofector (Lonza). Individual colonies were screened by PCR followed by HindIII digestion (ThermoFisher Scientific). Those clones with positive HindIII digestion were sequenced by Sanger sequencing. Clones showing bi‐allelic recombination and the desired genotype were expanded, cryopreserved, and karyotyped. The maintenance of the expression of the pluripotency marker and the ability to obtain the three germ layers was verified by immunofluorescence.

### iPSC differentiation to DA neurons

Seven different iPSCs, two different clones of two healthy controls (CONTROL 1 and CONTROL 2), two clones of THDA (THDA1#5 and THDA1#17), two clones of THDB (THDB1#1 and THDB1#15), and one isogenic (isoTHDA1#17), were differentiated into dopaminergic neurons using a 30‐day protocol based on DAn patterning factors and co‐culture with mouse PA6 feeding cells to provide trophic factor support, with minor modifications (Sánchez‐Danés *et al*, 2012). Specifically, iPSCs were cultured in mTeSR commercial medium until they reached 80% confluence and then mechanically aggregated to form embryoid bodies (EBs), without using lentiviral vectors to express LMX1A transcriptional factor. EBs were cultured for 10 days in suspension in N2B27 medium, consisting of DMEM/F12 medium (GIBCO), neurobasal medium (GIBCO), 0.5× B27 supplement (GIBCO), 0.5× N2 supplement (GIBCO), 2 mM ultraglutamine (Lonza) and penicillin–streptomycin (Lonza). In this step, N2B27 was supplemented with SHH (100 ng/ml, Peprotech), FGF‐8 (100 ng/ml, Peprotech), and bFGF (10 ng/ml; Peprotech). Neural progenitor cells (NPCs) were then seeded on top of PA6 for 21 days in N2B27 medium, as described (Sánchez‐Danés *et al*, 2012). Studied cultures were fixed with PFA 4% and characterized for dopaminergic specificity and for cell morphology.

### L‐Dopa and Carbidopa treatment

For the L‐Dopa and Carbidopa studies, NPCs were seeded on top of mouse PA6 cells and maintained in N2B27 medium, as described above. DAn were treated after 20 days in culture with L‐Dopa (50 μM; 3,4‐dihydroxy‐L‐phenylalanine, Sigma) and Carbidopa (12.5 μM; Sigma) in a ratio of 4:1 (Burbulla *et al*, 2017) for 10 days. Each compound was added three times a week at each change of the medium. For the early L‐Dopa studies, treatment was started on day 6 during the neural induction of EBs and maintained until the end of differentiation on day 30, for a total of 24 days.

### iPSC differentiation to neural cultures not‐enriched‐in‐DAn

An existing protocol, based on the synergistic action of two inhibitors of SMAD signaling, Noggin and SB431542, was adapted to generate neural progenitor cells (NPCs), which were then further induced to differentiate into neurons (not enriched in DAn) (Chambers *et al*, 2009). Briefly, the iPSCs were maintained in mTeSR™ medium until confluence. EBs were generated and maintained in suspension for 48 h. Subsequently, the medium was changed to a proneural medium (PN) and maintained for 5 days. PN medium consists of DMEM/F12 medium (GIBCO), neurobasal medium (GIBCO), 0.5× B27 minus vitamin A supplement (GIBCO), 0.5× N2 supplement (GIBCO), 2 mM Ultraglutamine (Lonza), β‐mercaptoethanol (Life Technologies), and penicillin–streptomycin (Lonza). To obtain neural rosettes, EBs were seeded on poly‐l‐ornithine/laminin (POLAM)‐coated plates with PN medium supplemented with Noggin (200 ng/ml; Peprotech) and SB431542 (10 μM; Tocris). After 8–12 days, the neural rosettes were picked and expanded in new POLAM‐coated dishes. Neural rosettes were enzymatically dissociated and seeded in new POLAM‐coated plates with PN medium supplemented with FGF2 (10 ng/ml; Peprotech) and EGF (10 ng/ml; Peprotech) to promote the generation and proliferation of neural progenitor cells (NPCs). After forming a homogenous cell population, the NPCs were further differentiated into GABA and glutamatergic neurons on POLAM‐coated plates with PN medium during 3–5 weeks.

### Protein extraction and Western blotting

For pellet collection, the cells were washed with cold PBS and mechanically detached from culture dishes and centrifuged for 5 min at 376 *g*. The cell pellets were then resuspended in RIPA protein extraction buffer (Sigma) supplemented with protease inhibitor (Roche) on ice. The resuspended pellets were then sonicated in a 10 s pulse of 10% amplitude on a Branson Digital Sonifier^®^ ultrasonic cell disruptor (Branson Ultrasonics Corporation) and left in a rotator for 1 h at 4°C to homogenize the proteins. The resulting supernatant was normalized for protein using the Bradford (Biorad) method. Total protein extracts were denatured in loading buffer for 5 min at 95°C. Each 15 μg protein sample was separated by 8% SDS polyacrylamide gel electrophoresis (SDS–PAGE) and transferred to a nitrocellulose membrane (Sigma). For D2DR and AADC detection, TGX Stain‐Free™ gels (Bio‐Rad) were prepared following the manufacturer's instructions. Proteins were transferred into methanol pre‐activated polyvinylidene fluoride (PVDF) membranes (Merck‐Millipore). Membranes were blocked with 5% not‐fat milk in 0.1 M Tris‐buffered saline (pH 7.4)/Tween (Sigma) 0.1% (TBSTween) for 1 h at room temperature and incubated overnight in TBS‐Tween containing rabbit anti‐TH (1:1,000; Santa Cruz), rabbit anti‐D2DR (1:500; Merck‐Millipore), or rabbit anti‐AADC (1:1,000; abcam) at 4°C. After incubation with peroxidase‐tagged secondary antibodies (1:2,000; Sigma) for 1 h at room temperature, membranes were revealed with the ECL‐plus chemiluminescence Western blot kit (Amersham‐Pharmacia Biotech). The intensity of the protein band was quantified using Fiji^®^ software. The optical density value of each band was corrected by the value of beta‐actin (1:2,000; Proteintech) for nitrocellulose membranes. Membranes transferred from TGX Stain‐Free™ gels were quantified and normalized for total protein using the Image Lab software (Bio‐Rad).

### Intracellular dopamine ELISA

Cells were harvested with cold PBS and centrifuged for 5 min at 135 *g*. The pellet obtained was diluted in 135 μl of sodium metabisulfite and EDTA both from Sigma to prevent the degradation of catecholamine. Next, the pellet was sonicated with a 10% amplitude for 60 s (10 s pulse alternating with 10 s rest in ice) repeated three times. About 115 μl of the 135 μl was stored for ELISA studies, and the remaining volume was used to determine protein concentration by the Bradford method. Intracellular DA was measured using an ELISA kit for DA (LDN) in duplicates. This determination is a two‐step process: first DA is extracted, acetylated, and activated and then enzymatically converted and detected with a competitive ELISA assay. The corresponding DA levels were normalized to the previously determined protein concentration.

### High‐performance liquid chromatography (HPLC)

The supernatant was collected from neurons differentiated on top of PA6 and kept directly at −80°C until the time of analysis. Before their analysis, the medium samples were previously deproteinized with 50 μl of homogenization medium (100 ml miliQ H2O, 100 mg of sodium metabisulphite (Sigma), 10 mg of EDTA‐Na (Sigma), 100 mg of cysteine (Sigma) and 3.5 ml of HClO_4_ concentrated (Scharlau, 70%)); centrifuged at 3,723 *g* for 30 min at 4°C, and the supernatant was filtered (0.45 μm, Millipore) for a posterior HPLC injection. The concentration of 3,4‐Dihydroxy‐L‐phenylalanine (L‐Dopa), dopamine (DA), and 3,4‐dihydroxyphenylacetic acid (DOPAC) in supernatant samples was determined using an HPLC system with a Waters 717 plus autosampler (Waters Cromatografia), a Waters 515 pump, a 5 μm particle size C18 column (100 × 46 mm, Kinetex EVO, Phenomenex), and a Waters 2465 amperometric detector set at an oxidation potential of 0.75 V. The mobile phase consisted of 0.15 M NaH_2_PO_4_.H_2_O, 0.57 mM 1‐octane sulfonic acid, 0.5 mM EDTA (pH 2.8, adjusted with phosphoric acid), and 7.4% methanol and was pumped at 0.9 ml/min. The total sample analysis time was of 50 min and the L‐Dopa, DA, and DOPAC retention times were 2.06, 3.94, and 4.25 min respectively. The detection limit was of 2–3 fmol (injection volume 60 μl). The corresponding content of the DA metabolite was normalized to the protein concentration previously determined by Bradford method detection.

### RT‐qPCR analyses

Cells were harvested with cold PBS and centrifuged for 5 min at 2,000 rpm. The pellet was kept directly at −80°C until the time of analysis. Total mRNA was isolated using the RNAeasy Mini kit (Qiagen). About 500 ng of total mRNA was used to synthesize cDNA with the SuperScript III First‐Strand Synthesis System (Thermofisher). Quantitative PCR (qPCR) analyses were performed in duplicate on 8 ng with PowerUp Syber Green Master Mix (Thermofisher) in an ABI Prism 7900HT Fast Real‐Time PCR System (Applied Biosystems), using the following program: 50°C for 2 min, 95°C for 2 min, 95°C for 15 s, and 60°C for 1 min (last two steps repeated 40 cycles). All results were normalized to beta‐actin and NSE. Primers used in the study are listed in Appendix Table S1.

### Immunocytochemistry

iPSCs or iPSC‐derived neurons were fixed using 4% paraformaldehyde for 20 min at room temperature, washed three times for 15 min with DPBS, and permeabilized with 0.1% Triton X‐100 in Tris‐buffered saline (TBS). Cells were then blocked for 2 h with 0.1% Triton X‐100 with 3% donkey serum. Primary antibodies prepared in blocking solution were incubated for 48 h at 4°C, while secondary antibodies (1:200; Alexa Fluor Series, Jackson Laboratories) were incubated for 2 h at room temperature after washing. Primary antibodies used include mouse anti‐ASMA (1:400; Sigma), goat anti‐FOXA2 (1:50; R&D Systems), goat anti‐Nanog (1:25; R&D Systems), mouse anti‐OCT3/4 (1:25; Santa Cruz), rabbit anti‐Sox2 (1:100; Thermofisher), rat anti‐SSEA3 (1:10; Iowa), mouse anti‐SSEA4 (1:10; Iowa), rabbit anti‐TH (1:250; Santa Cruz), mouse anti‐Tra‐1‐60 (1:100; Millipore), mouse anti‐Tra‐1‐81 (1:100; Millipore), and mouse anti‐TUJ1 (1:500; Covance). To visualize nuclei, samples were stained with DAPI (4,6‐diamidino iamidino‐2‐phenylindole) (Invitrogen, 1:5,000) for 10 min, mounted with PVA:DABCO, and stored at 4°C until imaged. Samples were imaged using a Carl Zeiss LSM880 confocal microscope and analyzed with FIJI® is Just ImageJ™ to quantify the percentage of TH/DAPI and TH/TUJ1 at day 30. An average of five images was quantified for each ratio, and each differentiation was performed at least three times.

### Neurite analysis and fiber density quantification

At the end of differentiation (on day 30), neurite analysis was performed on iPSC‐derived neurons differentiated on top of PA6, either treated or not with L‐Dopa and Carbidopa for 10 or 24 days (early L‐Dopa), fixed and stained for TH. We randomly selected a minimum of 10 DAn per iPSC line (in the only condition that were isolated from surrounding DAn, so that neurites could be unambiguously attributed to a single DAn), using a Carl Zeiss LSM880 confocal microscope and analyzed with the FIJI® is Just ImageJ™ plugin NeuronJ to determine the number and length of neurites per cell.

For fiber density quantification, we generated a mask using ImageJ to delimit the area of the image occupied by TH+ fibers which did not include nuclei (TH− stained area). The area was corrected by the number of TH+ neurons present in each image (Ishikawa *et al*, 2016; Prots *et al*, 2018).

### Data analysis and statistics

Number of independent experiments (*n*) is indicated in each figure legend. Normalized means across multiple experiments were used to generate graphs of each individual's mean. Statistical analyses of the obtained data were performed using ANOVA, Kruskal–Wallis, or Student's *t*/MannWhitney U‐tests according to the normality of comparisons and were plotted using Prism version 7.00 for Mac (GraphPad Software, La Jolla, CA, USA) with SEM error bars. ****P < 0.0001, ***P < 0.001, **P < 0.01 and *P < 0.05.

## Author contributions

**Alba Tristán‐Noguero:** Conceptualization; data curation; formal analysis; validation; investigation; visualization; methodology; writing – original draft; writing – review and editing. **Irene Fernández‐Carasa:** Data curation; formal analysis; validation; investigation; visualization; methodology; writing – review and editing. **Carles Calatayud:** Data curation; formal analysis; investigation; methodology. **Cristina Bermejo‐Casadesús:** Formal analysis; investigation; methodology. **Meritxell Pons‐Espinal:** Data curation; formal analysis; investigation; visualization; methodology. **Arianna Colini Baldeschi:** Data curation; formal analysis; investigation; methodology. **Leticia Campa:** Validation; investigation; methodology; writing – review and editing. **Francesc Artigas:** Resources. **Analía Bortolozzi:** Resources. **Rosario Domingo‐Jiménez:** Resources. **Salvador Ibáñez:** Resources. **Mercè Pineda:** Resources. **Rafael Artuch:** Resources. **Ángel Raya:** Resources; supervision; writing – original draft; writing – review and editing. **Àngels García‐Cazorla:** Conceptualization; resources; supervision; funding acquisition; validation; visualization; writing – original draft; writing – review and editing. **Antonella Consiglio:** Conceptualization; resources; supervision; validation; visualization; writing – original draft; writing – review and editing.

## Disclosure and competing interests statement

AGC and RA have received honoraria for lectures from PTC Therapeutics Inc. The other authors have no conflicts of interest to declare that are relevant to the content of this article.

**For more information**

- OMIM ENTRY: https://www.omim.org/entry/191290
- iNTD (international working group of neurotransmitter disorders): http://intd‐online.org/
- DeNeu (Spanish patients association of neurotransmitters disorders): https://www.deneu.org/
- Nord (organization of rare diseases patients). Rare disease database: https://rarediseases.org/rare‐diseases/tyrosine‐hydroxylase

## Supporting information



Appendix

Expanded View Figures PDF

Source Data for Expanded View and Appendix

PDF+

Source Data for Figure 2

Source Data for Figure 3

Source Data for Figure 4

Source Data for Figure 5

## Data Availability

This study does not include data deposited in external repositories.
