# Supplementary material for: iPSC‐based modeling of THD recapitulates disease phenotypes and reveals neuronal malformation
Source: EMBO Mol Med. 2023 Feb 6;15(3):e15847. doi: 10.15252/emmm.202215847 (PMC9994475; doi:10.15252/emmm.202215847)
Supplement: Supplementary file 1 — Appendix [file EMMM-15-e15847-s007.pdf]

iPSC-based modeling of THD recapitulates disease phenotypes and reveals neuronal malformation

APPENDIX

Alba Tristán-Noguero[1], Irene Fernández-Carasa[2,3], Carles Calatayud[2,3,4], Cristina Bermejo-Casadesús[1], Meritxell Pons-Espinal[2,3], Arianna Colini Baldeschi[2,3], Leticia Campa[5], Francesc Artigas[5], Analia Bortolozzi[5], Rosario Domingo-Jiménez[6,7], Salvador Ibáñez[6], Mercè Pineda[8], Rafael Artuch[7,9], Ángel Raya[4,10,11], Àngels García-Cazorla[1,7*] and Antonella Consiglio [2,3,12*]

1 Neurometabolic Unit and Synaptic Metabolism Lab, Neurology Department, Institut Pediàtric de Recerca, Hospital Sant Joan de Déu, Barcelona, Spain
2 Department of Pathology and Experimental Therapeutics, Bellvitge University Hospital-IDIBELL, Hospitalet de Llobregat, Barcelona, Spain
3 Institute of Biomedicine of the University of Barcelona (IBUB), Barcelona, Spain
4 Regenerative Medicine Program, Bellvitge Biomedical Research Institute (IDIBELL), and Program for Translation of Regenerative Medicine in Catalonia (P-[CMRC]), Hospital Duran i Reynals, Hospitalet de Llobregat, Barcelona, Spain
5 Institut d'Investigacions Biomèdiques de Barcelona (IIBB), Spanish National Research Council (CSIC), Barcelona, Spain; Institut d'Investigacions August Pi i Sunyer (IDIBAPS), Barcelona, Spain; Centro de Investigación Biomédica en Red de Salud Mental (CIBERSAM), ISCIII, Madrid, Spain
6 Department of Pediatric Neurology, Hospital Virgen de la Arrixaca; Instituto Murciano de Investigación Biosanitaria Virgen de la Arrixaca (IMIB), Murcia, Spain
7 Centro de Investigación Biomédica En Red Enfermedades Raras (CIBERER), Madrid, Spain
8 Fundació Sant Joan de Déu (FSJD), Hospital Sant Joan de Déu (HSJD), Barcelona, Spain
9 Metabolic Unit, Departments of Neurology, Nutrition Biochemistry and Genetics, Institut Pediàtric de Recerca, Hospital San Joan de Déu, Barcelona, Spain
10 Centre for Networked Biomedical Research on Bioengineering, Biomaterials and Nanomedicine (CIBER-BBN), Madrid, Spain
11 Institució Catalana de Recerca i Estudis Avançats (ICREA), Barcelona, Spain
12 Department of Molecular and Translational Medicine, University of Brescia, Italy

*Corresponding authors: Antonella Consiglio and Àngels García Cazorla
Tel: +34932607214; E-mail: consiglio@ub.edu
Tel: +34936009751; E-mail: agarcia@sjdhospitalbarcelona.org

Running title: THD human stem cell model

Table of contain:

1. **Appendix Table:**

Appendix table S1. Primers used for gene expression analysis (page number 3)

2. **Appendix Figures:**

Appendix Fig S1. Expression of dopaminergic markers in iPSC-derived DAn and iPSC-derived neural cultures not-enriched-in-DAn (page number 4).

Appendix Fig S2. Relative mRNA expression of other DA markers (page number 5).

Appendix table S1: Primers used for gene expression analysis.

Gene	Forward primer (5'-3')	Reverse primer (5'-3')
Tyrosine hydroxylase (TH)	TGTAAGCAGAACGGGGAGGT	AGCTTGTCCTTGGCGTCACT
Vesicular monoamine transporter 2 (VMAT2)	ATGAGTTTGTGGGGAAGACG	TGTTTGCAAAGCAGATGGAG
Aromatic L-amino acid decarboxylase (AADC)	GAACAGACTTAACGGGAGCCTTT	AATGCCGGTAGTCAGTGATAAGC
Dopaminergic receptor 1 (D1DR)	CTTAGGATGCTACAGACTTTGCCCTG	CATGTGGGATCAGGTAAACCAGATTG
Dopaminergic receptor 2 (D2DR)	TCTTCGGACTCAATAACGCAGACC	GATGTAGACCAGCAGGGTGACAAT
Dopamine transporter (DAT)	ATCCTGCAATGGGAGAGACACGAA	ATTACAGCAACACAAGACACGGCG
G Protein-Activated Inward Rectifier Potassium Channel 2 (GIRK2)	CATGGATCAGGACGTCGAAA	GCCTGCTTAGGCAACTTTGG
Paired Like Homeodomain 3 (PITX3)	CAGAGGACGGTTCGCTGAAAA	AGCTGCCTTTGCATAGCTCG
LIM Homeobox Transcription Factor 1 Alpha (LMX1A)	ACGTCCGAGAACCATCTTGAC	CACCACCGTTTGTCTGAGC
Forkhead Box A2 (FOXA2)	CCACCACCAACCCCACAAAATG	TGCAACACCGTCTCCCCAAAGT
Nuclear Receptor Subfamily 4 Group A Member 2 (NR4A2/NURR1)	ACCACTCTTCGGGAGAATACA	GGCATTTGGTACAAGCAAGGT
Engrailed Homeobox 1 (EN1)	GCACACGTTATTCGGATCG	GCTTGTCCTCCTTCTCGTTCT
Beta-actin	AGGCCAACCGCGAGAAG	ACAGCCTGGATAGCAACGTACA

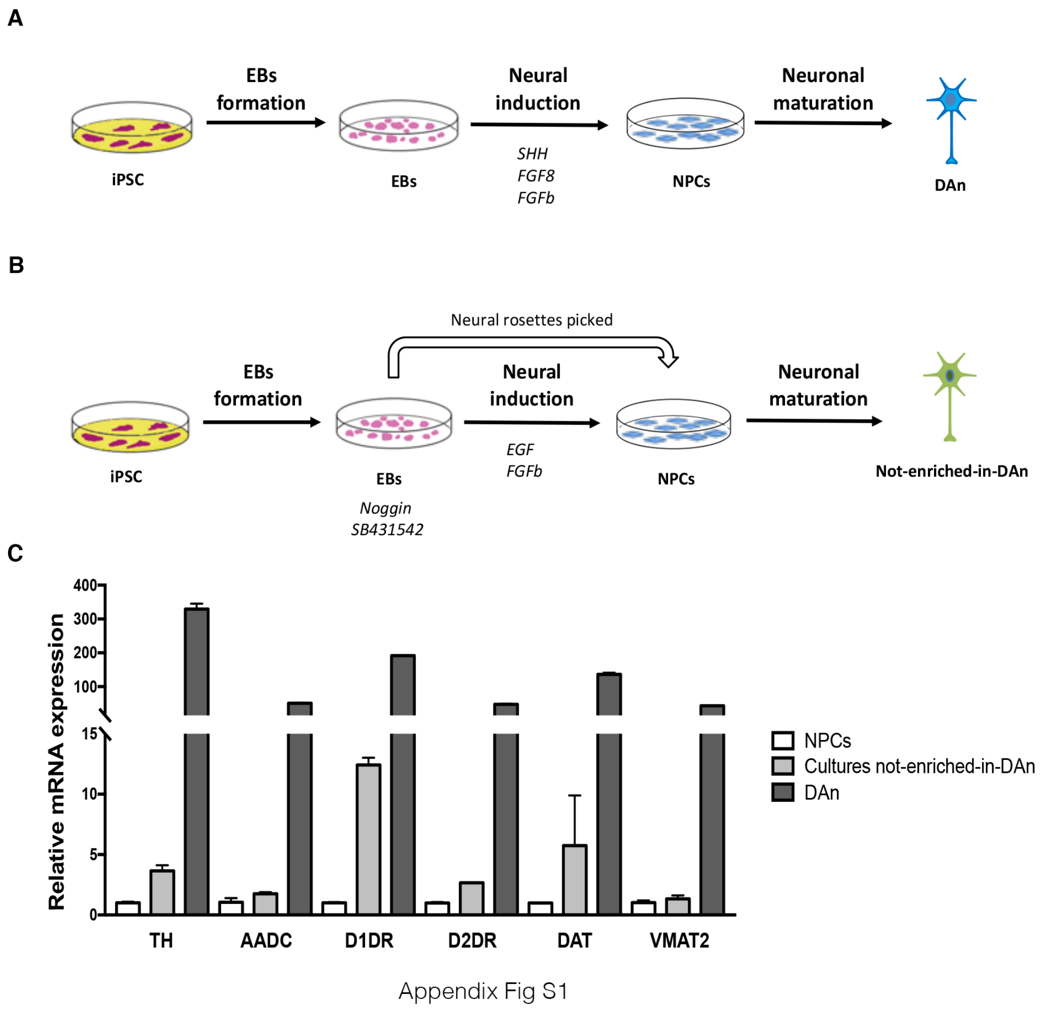

Appendix Fig S1. Expression of dopaminergic markers in iPSC-derived DAn and iPSC-derived neural cultures not-enriched-in-DAn. A Scheme of iPSC dopaminergic neuron differentiation. B Schematic diagram showing the iPSC differentiation into neural lineages (not-enriched-in-DAn). C Relative mRNA expression (assessed by qRT-PCR) of different dopaminergic genes such as *TH, AADC, D1DR, D2DR, DAT,* and *VMAT2*, relative to actin expression (*n*=2 independent experiments for all genes analyzed except for TH that includes *n*=3 experiments).

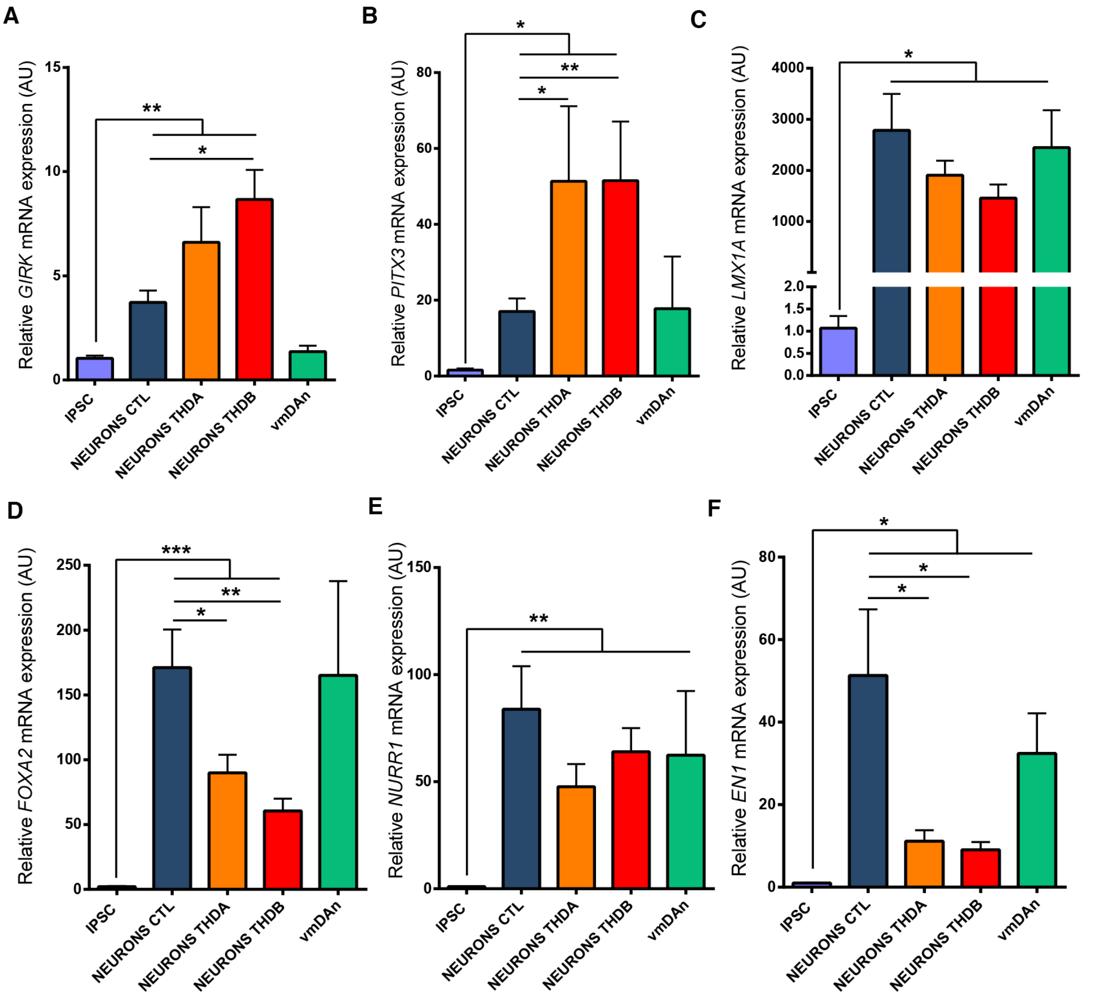

Appendix Fig S2

Appendix Fig S2. Relative mRNA expression of other DA markers. A Relative *GIRK* mRNA expression. **B** Relative *PITX3* mRNA expression. **C** Relative *LMX1A* mRNA expression. **D** Relative *FOXA2* mRNA expression. **E** Relative *NURR1* mRNA expression. **F** Relative *EN1* mRNA expression. Ventral midbrain dopaminergic neurons (vmDAn) were used as positive control and iPSCs were used as negative control. *N*=6 independent experiments except for iPSC samples (*n*=3). Data are expressed as mean ± SEM. ANOVA or Kruskal-Wallis tests were performed for multiple comparisons. Unpaired two-tailed Student's t test or Mann-Whitney U test was used for pairwise comparisons. ***p<0.001 **p<0.01 *p<0.05.
