# Supplementary material for: iPSC‐based modeling of THD recapitulates disease phenotypes and reveals neuronal malformation
Source: EMBO Mol Med. 2023 Feb 6;15(3):e15847. doi: 10.15252/emmm.202215847 (PMC9994475; doi:10.15252/emmm.202215847)
Supplement: Supplementary file 2 — Expanded View Figures PDF [file EMMM-15-e15847-s004.pdf]

Expanded View Figures

Figure EV1. Isogenic control from p.Arg233His mutation carrier was generated and shows *bona fide* pluripotent stem cell features.

A A region of the exon 6 with the ssODN, the gRNA, the PAM sequence, the mutation repaired, and the new restriction site for HindIII.
B PCR of exon 6 (left) and digestion of this PCR product with HindIII (right) (green boxes show digested PCR products).
C Sequence of a positive clone (3F) where the mutation was repaired, the sequence PAM is inactivated, and a new restriction site is introduced; all in homozygosis.
D Representative images of isoTHDA1#17 iPSC stained positive for the pluripotency-associated markers OCT4, NANOG, Sox2, SSEA3, TRA-1-81, SSEA4, and Tra1-60 and for the three primary germ cell layers, including endoderm (stained for FOXA2, red), mesoderm (stained for αSMA, green), and ectoderm (stained for TUJ1, green). Nuclei are counterstained with DAPI, shown in blue. Scale bars, 100 μm.
E Normal karyotype of isoTHDA1#17 iPSC at passage 25.

© 2023 The Authors

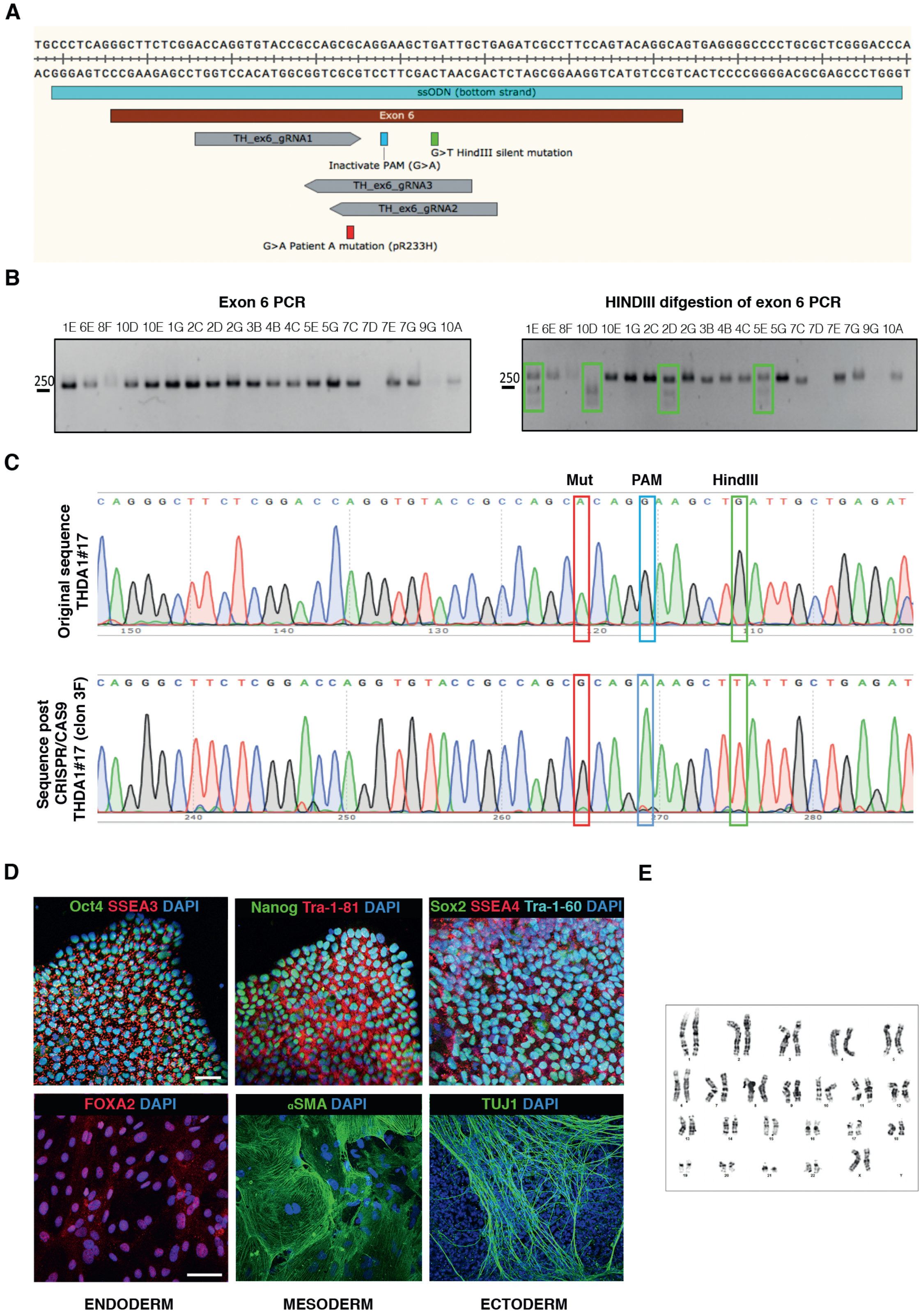
A
TGCCCTCAGGGCTTCTCGGACCAGGTGTACCGCCAGCGCAGGAAGCTGATTGCTGAGATCGCCTTCCAGTACAGGCAGTGAGGGGCCCCTGCGCTCGGGACCCA
ACGGGAGTCCCGAAGAGCCTGGTCCACATGGCGGTCGCGTCCTTCGACTAACGACTCTAGCGGAAGGTCATGTCCGTCACTCCCCGGGGACGCGAGCCCTGGGT
ssODN (bottom strand)
Exon 6
TH_ex6_gRNA1
G>T HindIII silent mutation
Inactivate PAM (G>A)
TH_ex6_gRNA3
TH_ex6_gRNA2
G>A Patient A mutation (pR233H)
B
Exon 6 PCR
1E 6E 8F 10D 10E 1G 2C 2D 2G 3B 4B 4C 5E 5G 7C 7D 7E 7G 9G 10A
250
HINDIII difgestion of exon 6 PCR
1E 6E 8F 10D 10E 1G 2C 2D 2G 3B 4B 4C 5E 5G 7C 7D 7E 7G 9G 10A
250
C
Mut
PAM
HindIII
Original sequence THDA1#17
Sequence post CRISPR/CAS9 THDA1#17 (clon 3F)
D
Oct4 SSEA3 DAPI
Nanog Tra-1-81 DAPI
Sox2 SSEA4 Tra-1-60 DAPI
FOXA2 DAPI
αSMA DAPI
TUJ1 DAPI
ENDODERM
MESODERM
ECTODERM
E

Figure EV1.

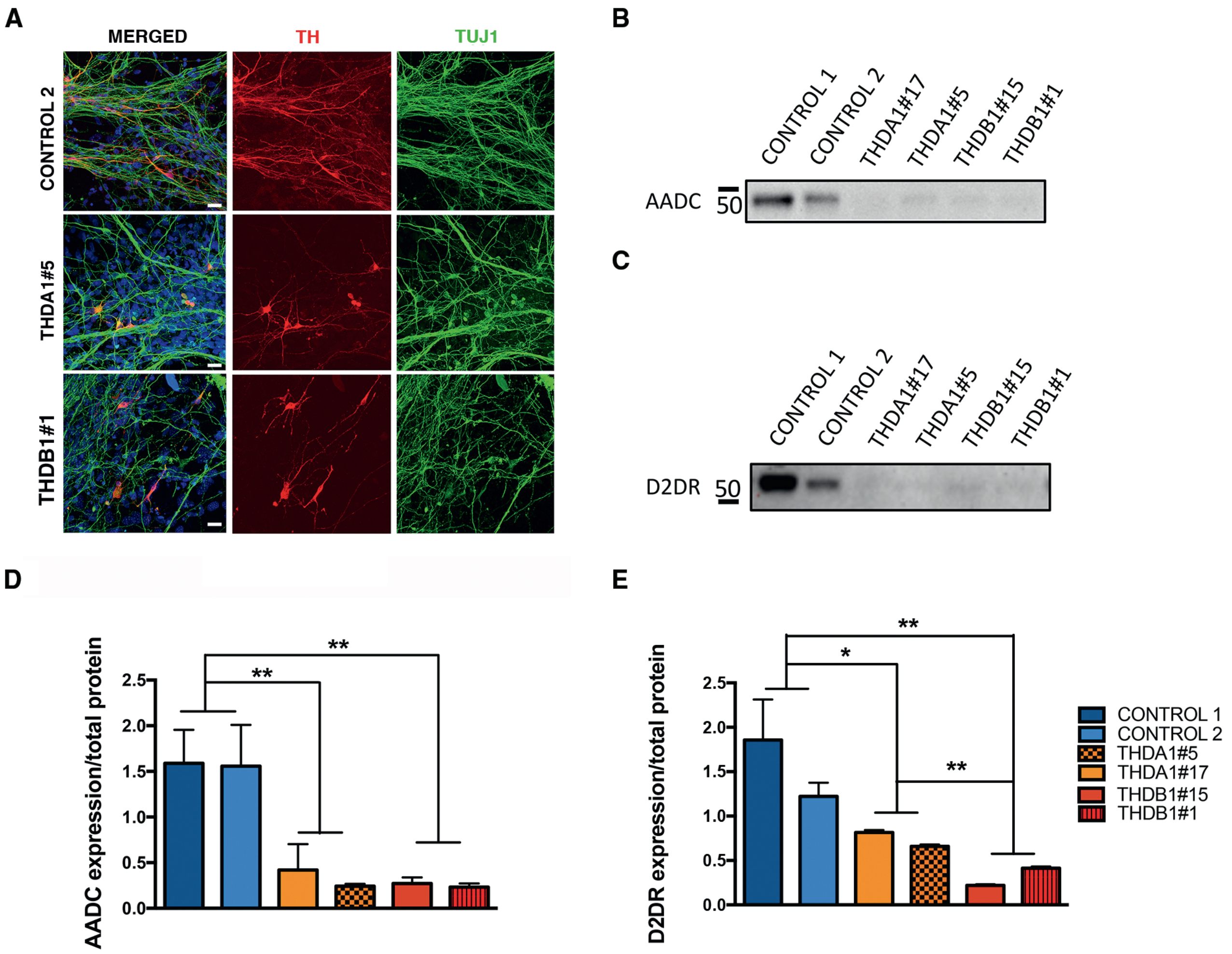

Figure EV2. iPSC-derived neurons from THDA1#5 and THDB1#1 clones recapitulate the reduced number of TH+ neurons.

A Representative immunofluorescence (IF) images of CONTROL 2, THDA1#5, and THDB1#1 iPSC neuronal cultures (TH in red, TUJ1 in green, and DAPI in blue) at day 30 of differentiation. Scale bars, 20 μm.

B, C Western blot of AADC (B) and D2DR (C) expression in CONTROL 1, CONTROL 2, THDA1#5, THDA1#17, THDB1#15, and THDB1#1 iPSCs neuronal cultures.

D, E Quantification of Western blot results for AADC (D) and D2DR (E) normalized for total protein (D, $n = 3$ experiments; E, $n = 2$ experiments). Data are expressed as mean ± SEM. Unpaired two-tailed Student's *t*-test or Mann–Whitney U-test was used for pairwise comparisons. $^{**}P < 0.01$; $^{*}P < 0.05$.

Source data are available online for this figure.

 © 2023 The Authors

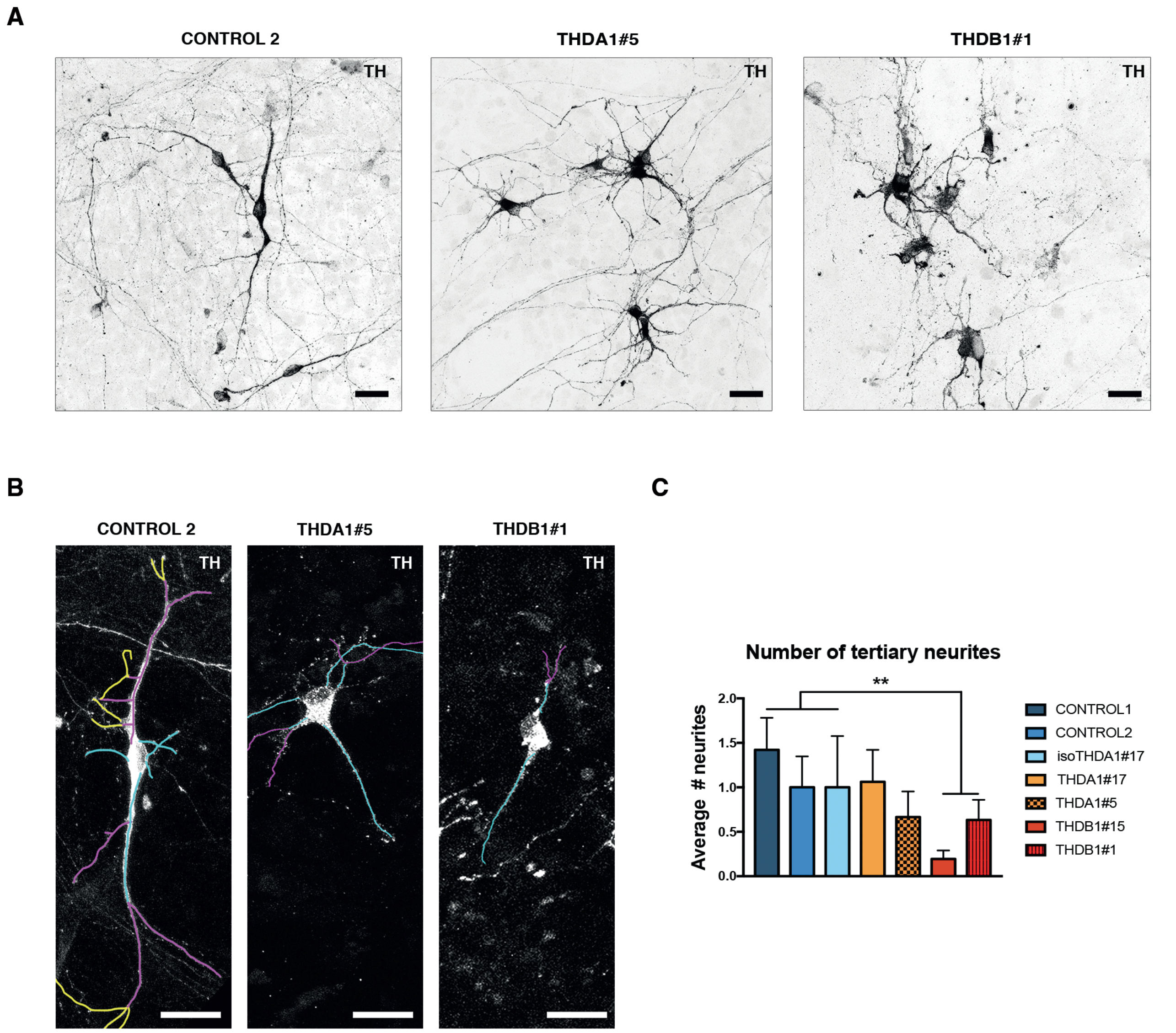

Figure EV3. iPSC-derived neurons from THDA1#5 and THDB1#1 clones show abnormal morphology.

A Immunofluorescence (IF) images of neuronal cultures (TH in black) of the same lines.

B Images of the tracing analysis (TH in white, primary neurites in blue, secondary in magenta, and tertiary in yellow) in CONTROL 2, THDA1#5, and THDB1#1 neuronal cultures.

C Number of TH tertiary neurites in all cell lines (CONTROL 1, CONTROL 2, isoTHDA1#17, THDA1#17, THDA1#5, THDB1#15, and THDB1#1).

Data information: Scale bars, 20 μm ($n = 3$ experiments, at least 10 neurons counted per experiment). Data are expressed as mean ± SEM. ANOVA test was used for multiple comparisons. $^{**}P < 0.01$.
Source data are available online for this figure.

© 2023 The Authors

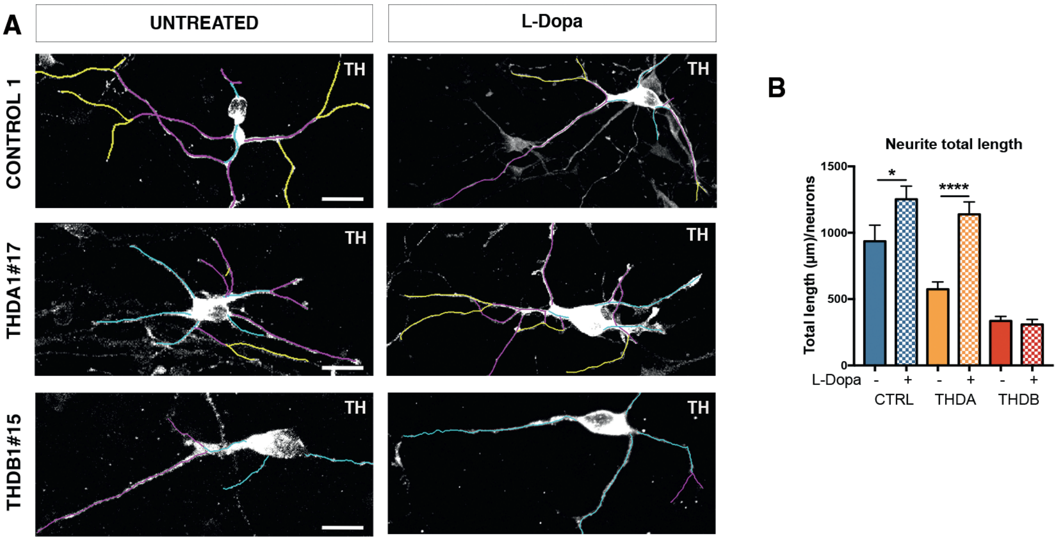

Figure EV4. L-Dopa and Carbidopa treatment rescue neurite total length in THDA.

A Representative images of the tracing analysis (TH in white, primary neurites in blue, secondary in magenta and tertiary in yellow) of CONTROL 1, THDA1#17, and THDB1#15 lines.

B Quantification of the total neurite length in CONTROL 1, THDA1#17, and THDB1#15 untreated and treated cultures.

Data information: Scale bars, 20 µm ($n = 3$, experiments per iPSC line; at least 10 neurons counted per experiment). Data are expressed as mean ± SEM. Unpaired two-tailed Student's t-test or Mann–Whitney U-test was used for pairwise comparisons. ****$P < 0.0001$; *$P < 0.05$.
Source data are available online for this figure.

 © 2023 The Authors

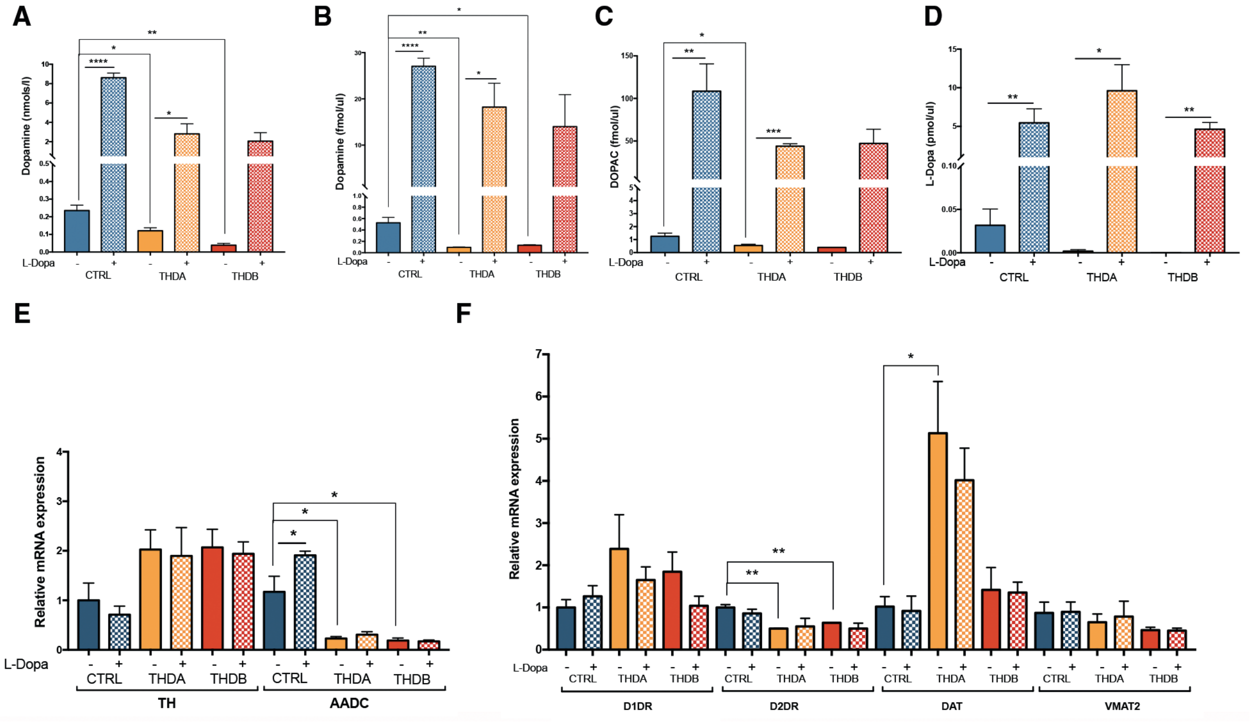

Figure EV5. L-Dopa and Carbidopa treatment partially rescues THDA phenotype.

A ELISA quantification of intracellular dopamine levels (nmols/l) in CONTROL 1, THDA1#17 and THDB1#15 lines before and after treatment.
B HPLC quantification of dopamine levels (fmols/μl).
C HPLC quantification of DOPAC levels (fmols/μl).
D HPLC quantification of L-Dopa levels (fmols/μl).
E Relative mRNA expression of DA enzymes (*TH* and *AADC*).
F Dopamine receptors (*D1DR* and *D2DR*), *DAT*, and *VMAT2* mRNA expression levels relative to Neural Specific Enolase (*NSE*) in CONTROL 1, THDA1#17, and THDB1#15.

Data information: $n = 3$, experiments per iPSC line except for L-Dopa treated THDA1#17 and THDB1#15 in panel (A); untreated THDB1#15 in panel (B) and (C); untreated CONTROL 1 in panel (C) that includes $n = 2$ experiments. Data are expressed as mean ± SEM. Unpaired two-tailed Student's *t*-test or Mann–Whitney U-test was used for pairwise comparisons. ****$P < 0.0001$; ***$P < 0.001$; **$P < 0.01$; *$P < 0.05$.
Source data are available online for this figure.

© 2023 The Authors
