# Supplementary figures and images for: iPSC‐based modeling of THD recapitulates disease phenotypes and reveals neuronal malformation
Source: EMBO Mol Med. 2023 Feb 6;15(3):e15847. doi: 10.15252/emmm.202215847 (PMC9994475; doi:10.15252/emmm.202215847)

## Uncropped WB Figure EV2B: AADC

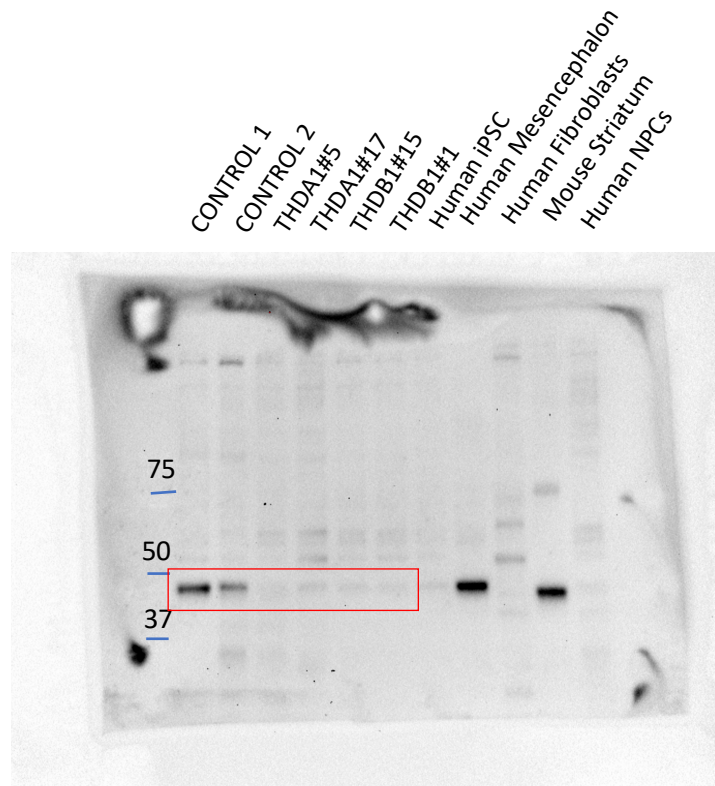

Supplement: Supplementary file 3 — Source Data for Expanded View and Appendix [file EMMM-15-e15847-s001.zip › Source Data for Expanded View and Appendix/Expanded View/EV2/EV2B.pdf]

## Uncropped WB Figure EV2C: D2DR

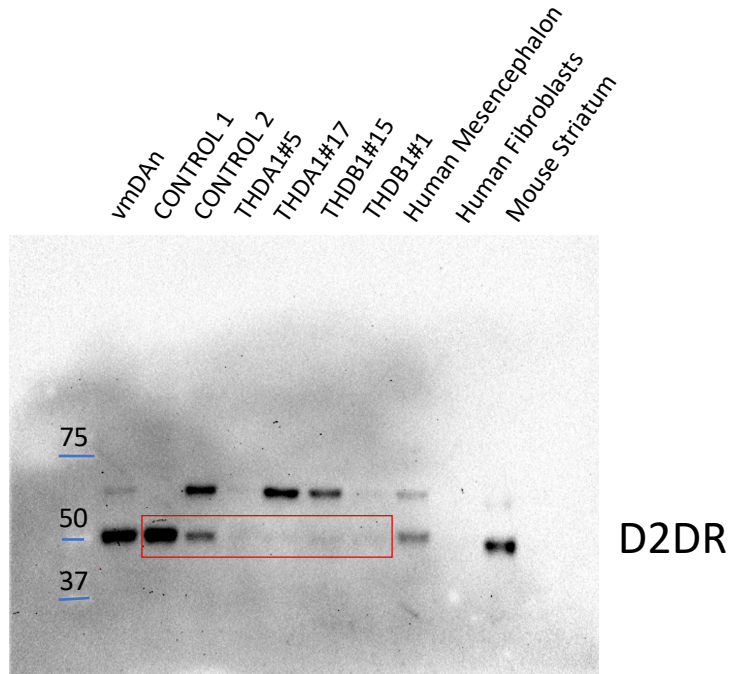

Supplement: Supplementary file 3 — Source Data for Expanded View and Appendix [file EMMM-15-e15847-s001.zip › Source Data for Expanded View and Appendix/Expanded View/EV2/EV2C.pdf]

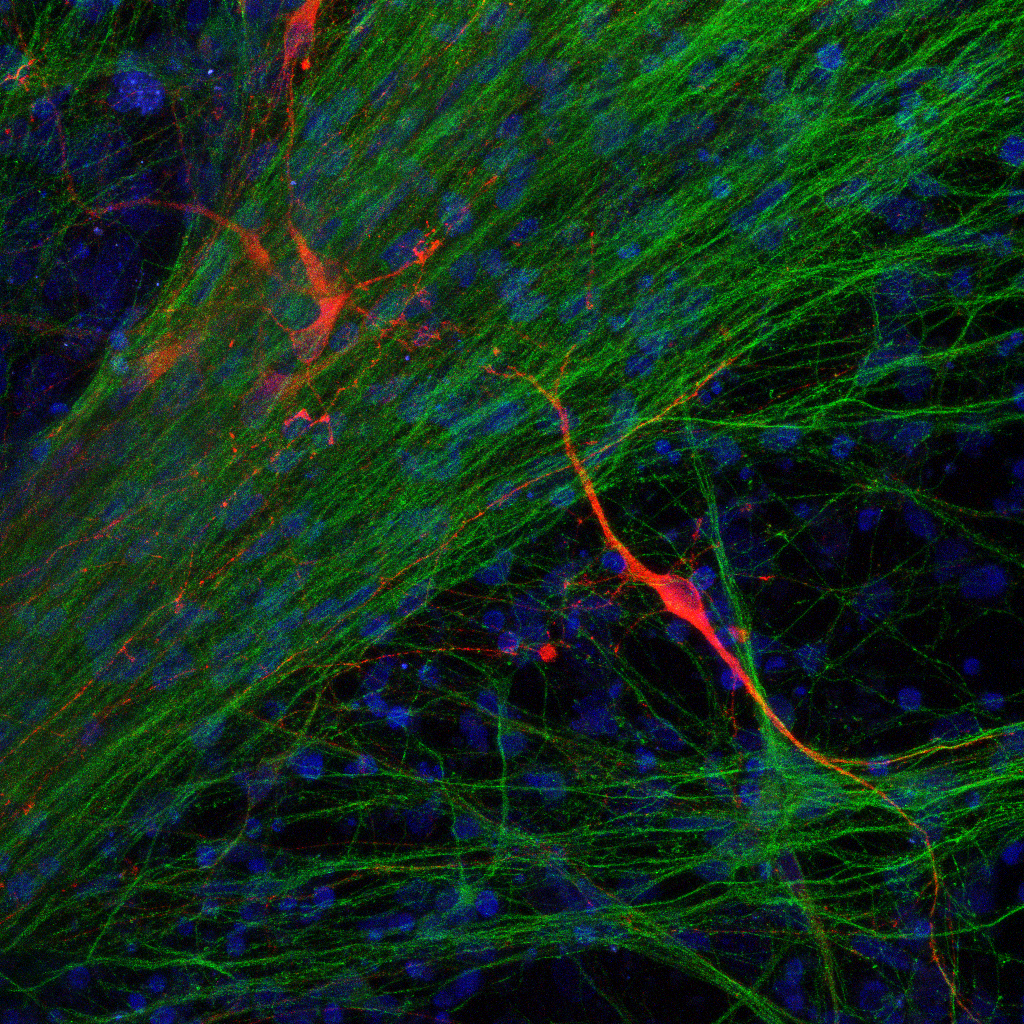

Supplement: Supplementary file 3 — Source Data for Expanded View and Appendix [file EMMM-15-e15847-s001.zip › Source Data for Expanded View and Appendix/Expanded View/EV3/EV3B/Fig EV3B CONTROL 2.tif]

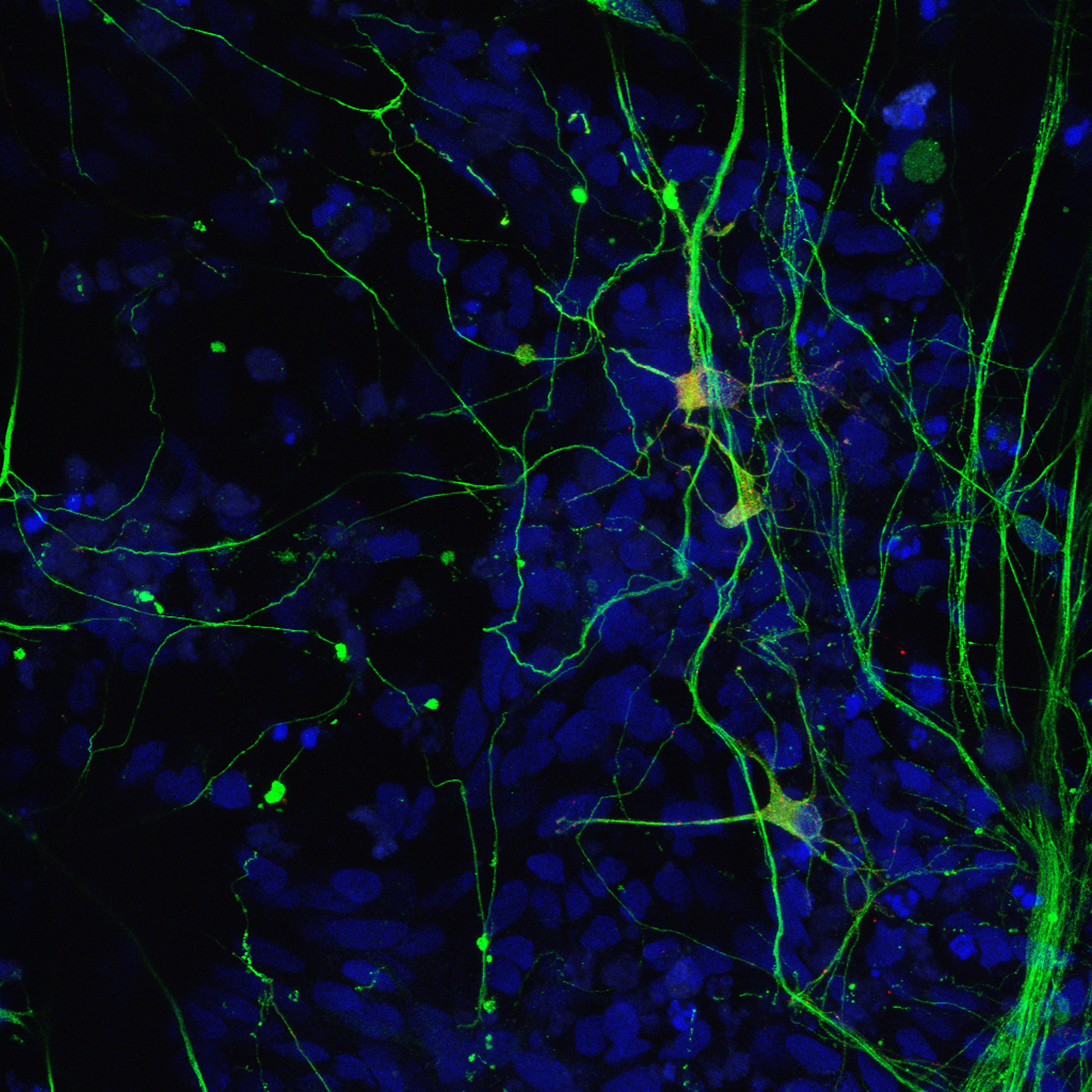

Supplement: Supplementary file 3 — Source Data for Expanded View and Appendix [file EMMM-15-e15847-s001.zip › Source Data for Expanded View and Appendix/Expanded View/EV3/EV3B/Fig EV3B THDA1#5.tif]

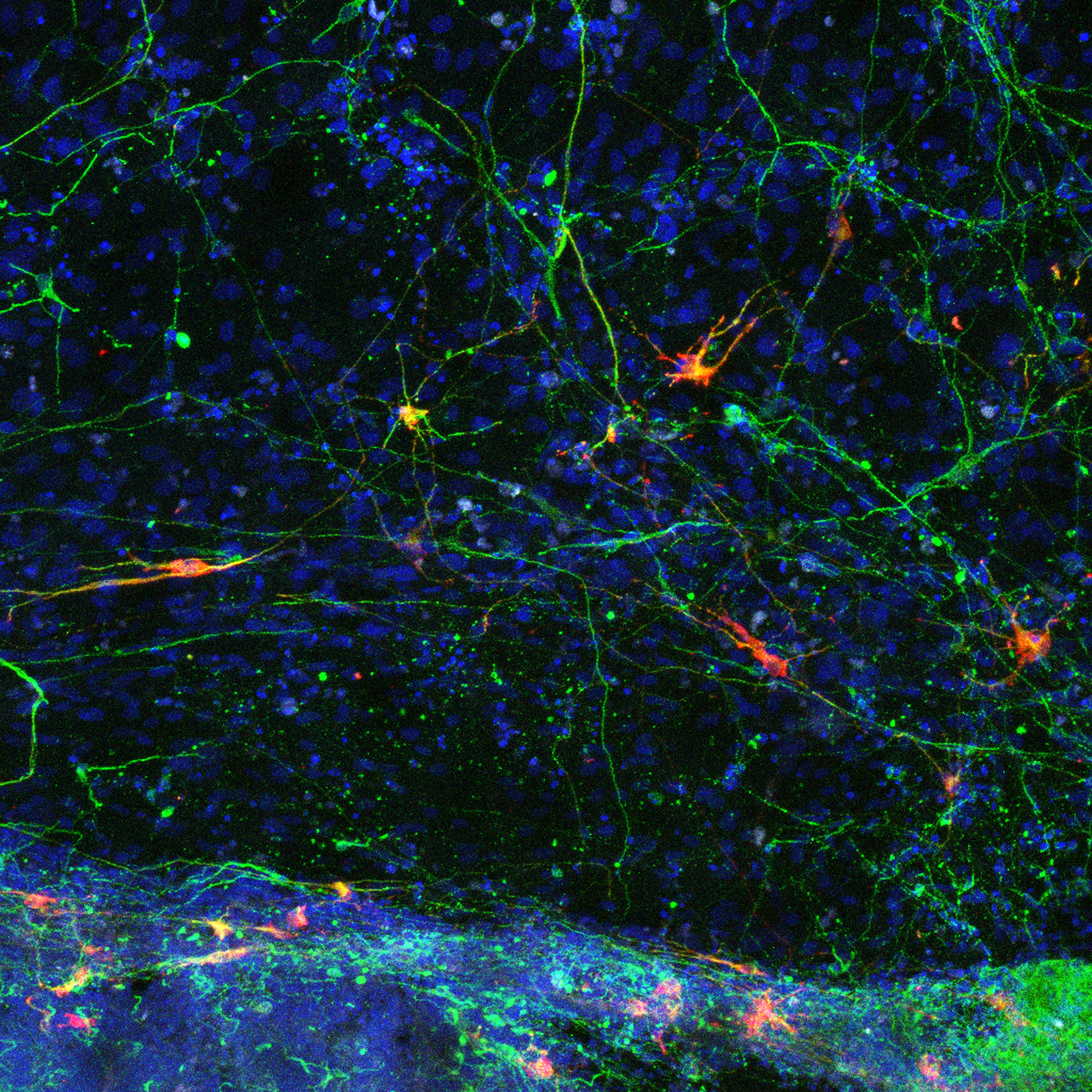

Supplement: Supplementary file 3 — Source Data for Expanded View and Appendix [file EMMM-15-e15847-s001.zip › Source Data for Expanded View and Appendix/Expanded View/EV3/EV3B/Fig EV3B THDB1#1.tif]

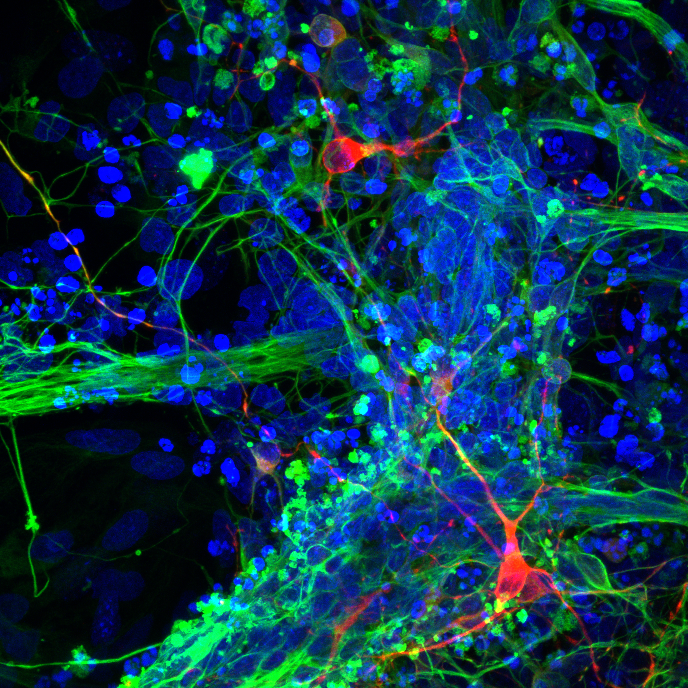

Supplement: Supplementary file 3 — Source Data for Expanded View and Appendix [file EMMM-15-e15847-s001.zip › Source Data for Expanded View and Appendix/Expanded View/EV4/EV4A/Fig EV4A CONTROL 1 L-Dopa.tif]

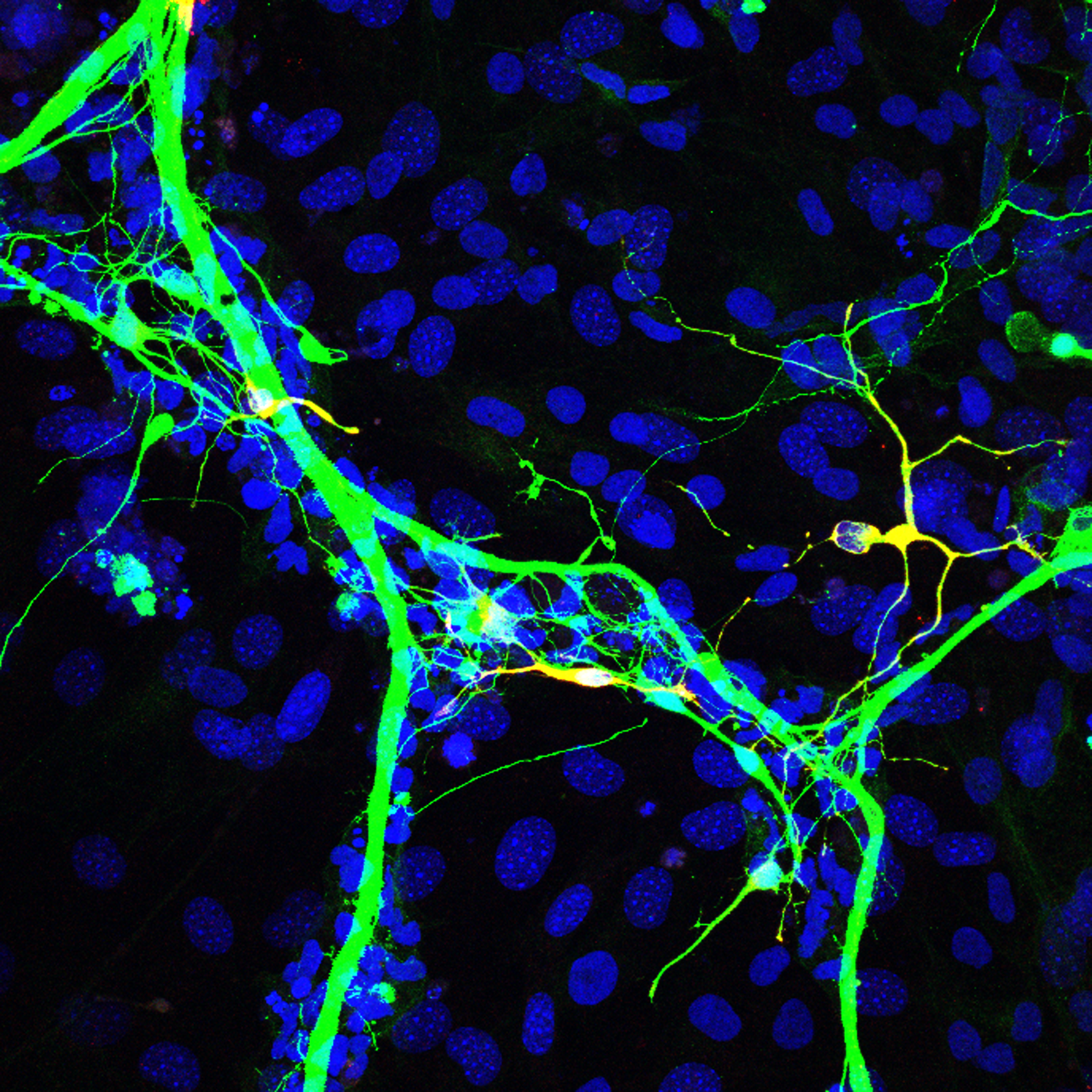

Supplement: Supplementary file 3 — Source Data for Expanded View and Appendix [file EMMM-15-e15847-s001.zip › Source Data for Expanded View and Appendix/Expanded View/EV4/EV4A/Fig EV4A CONTROL 1 UNTREATED.tif]

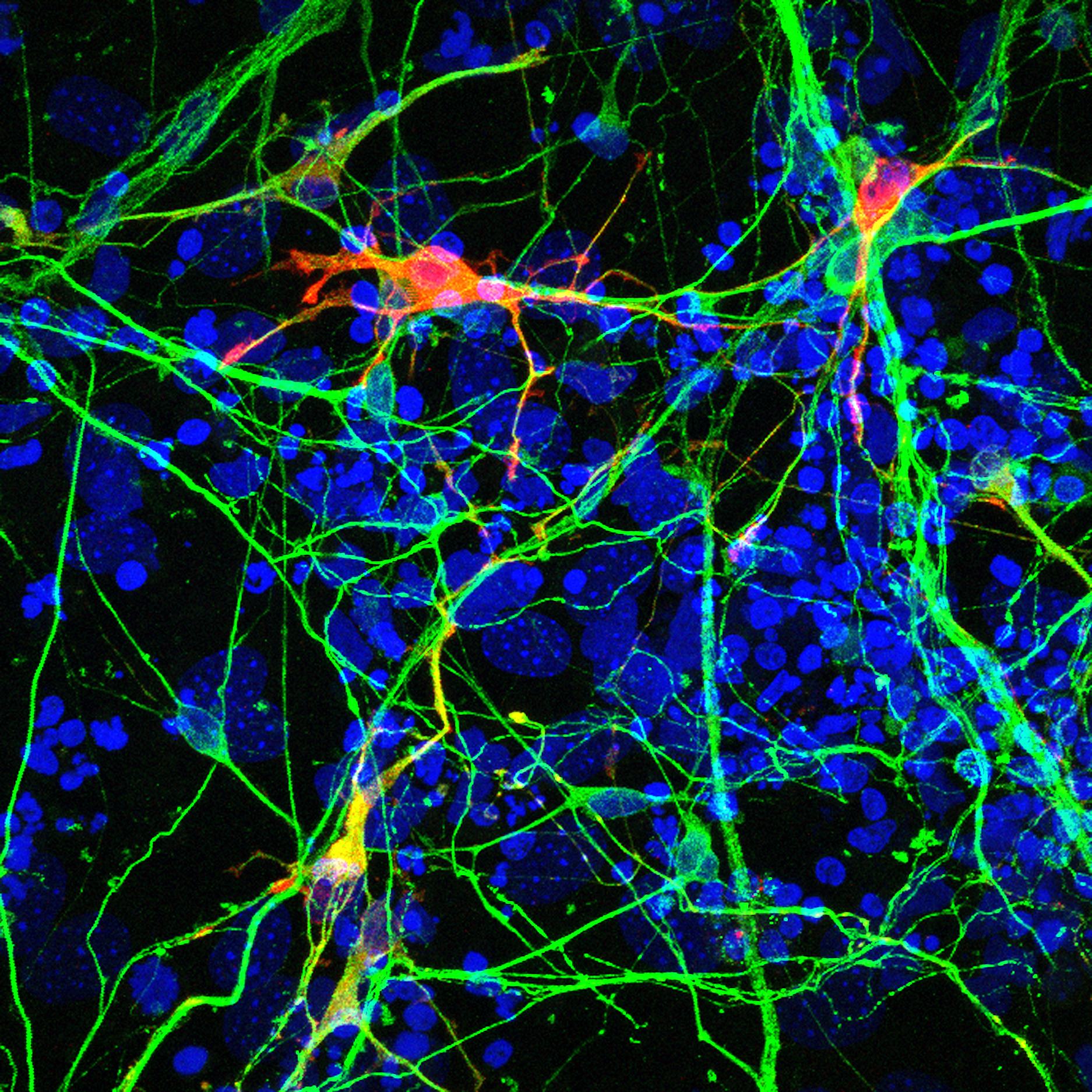

Supplement: Supplementary file 3 — Source Data for Expanded View and Appendix [file EMMM-15-e15847-s001.zip › Source Data for Expanded View and Appendix/Expanded View/EV4/EV4A/Fig EV4A THDA1#17 L-Dopa.tif]

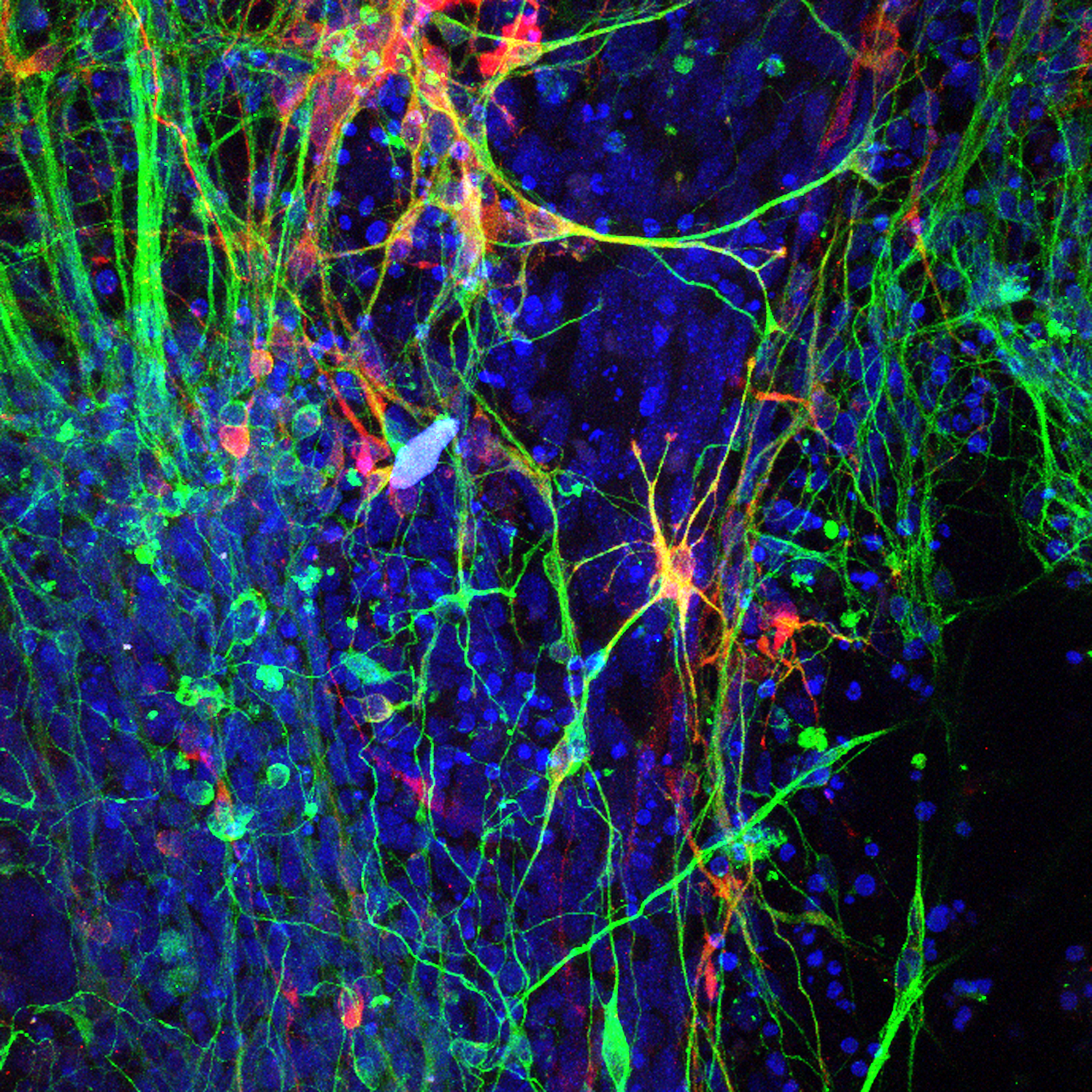

Supplement: Supplementary file 3 — Source Data for Expanded View and Appendix [file EMMM-15-e15847-s001.zip › Source Data for Expanded View and Appendix/Expanded View/EV4/EV4A/Fig EV4A THDA1#17 UNTREATED.tif]

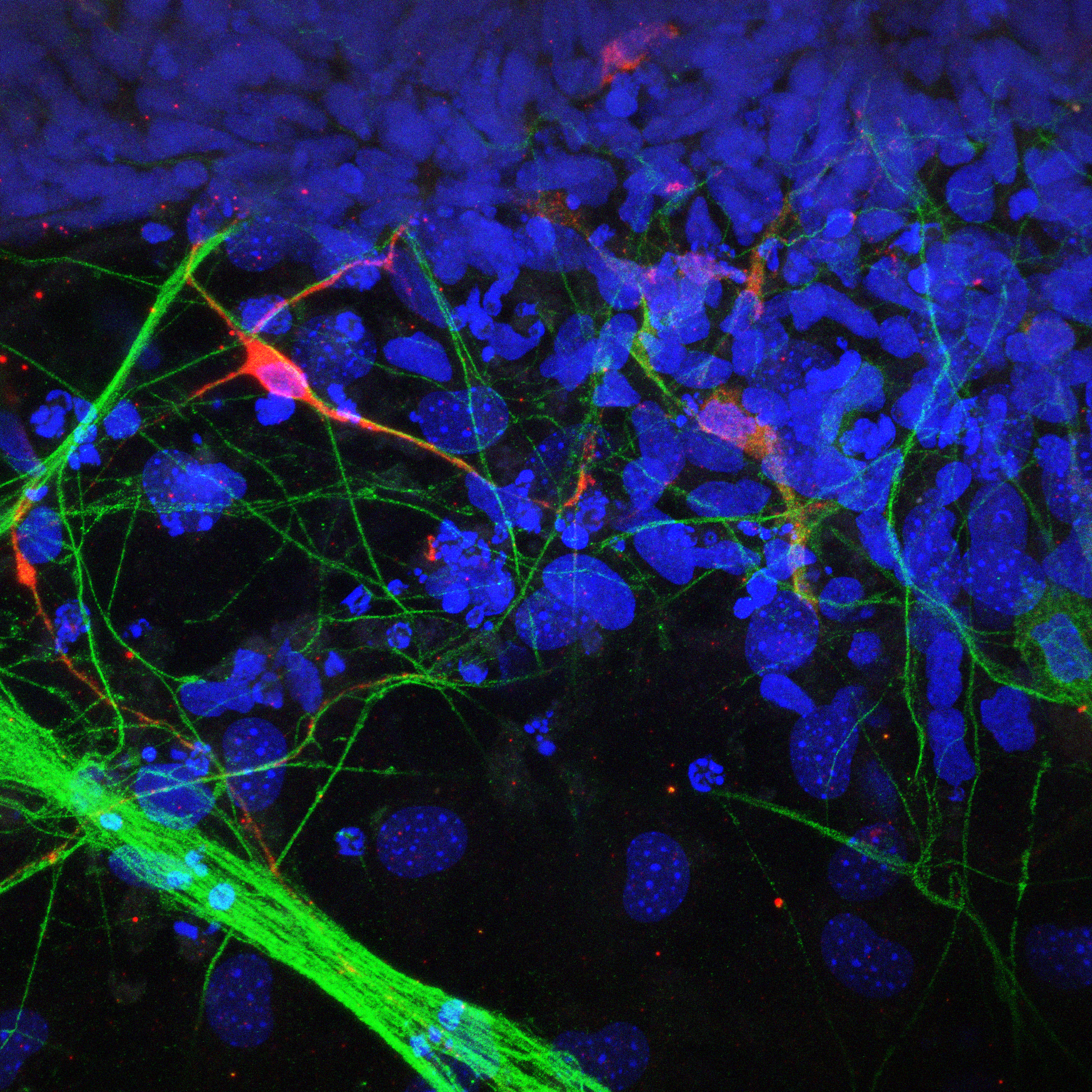

Supplement: Supplementary file 3 — Source Data for Expanded View and Appendix [file EMMM-15-e15847-s001.zip › Source Data for Expanded View and Appendix/Expanded View/EV4/EV4A/Fig EV4A THDB1#15 L-Dopa.tif]

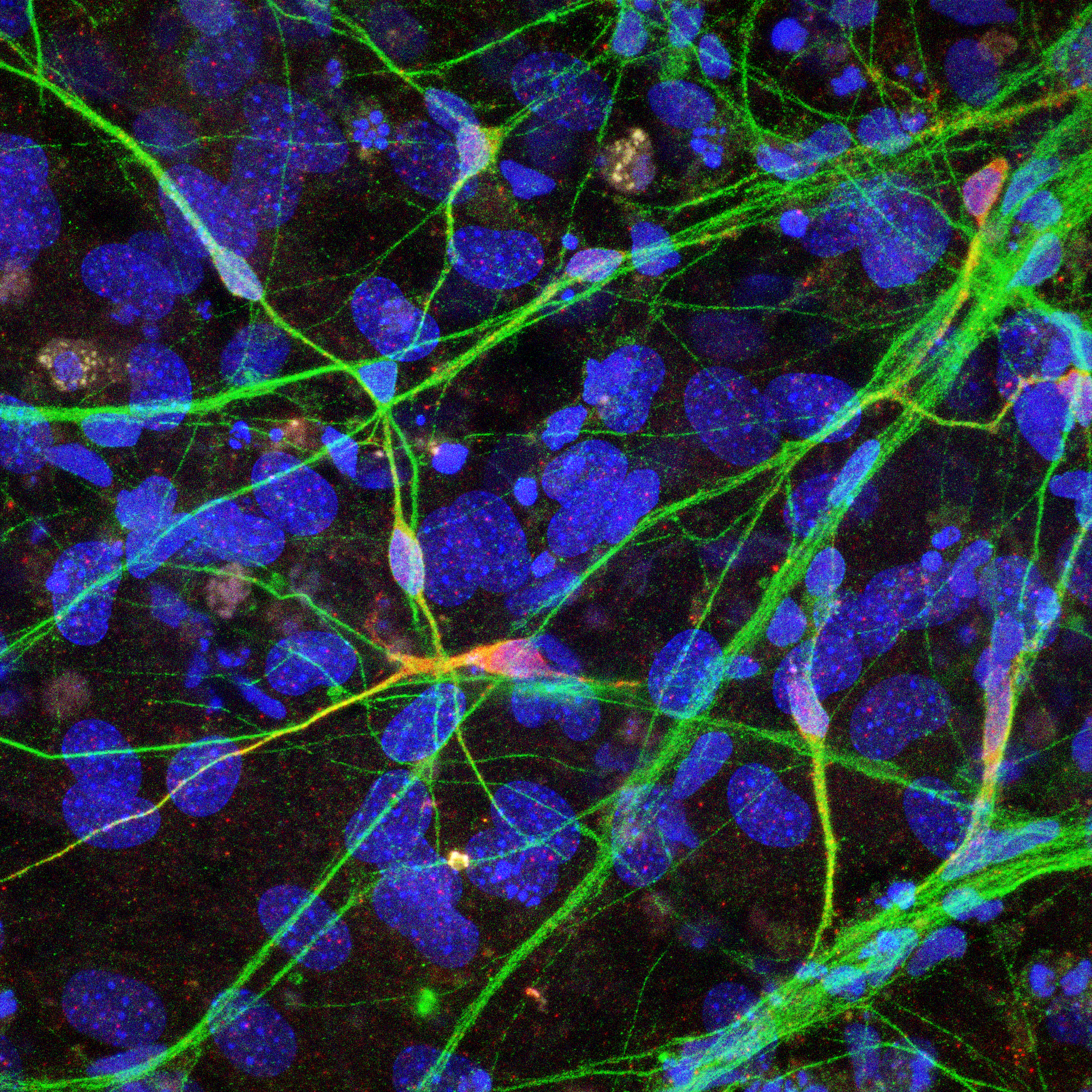

Supplement: Supplementary file 3 — Source Data for Expanded View and Appendix [file EMMM-15-e15847-s001.zip › Source Data for Expanded View and Appendix/Expanded View/EV4/EV4A/Fig EV4A THDB1#15 UNTREATED.tif]

## Uncropped WB Figure 2C: TH and actin

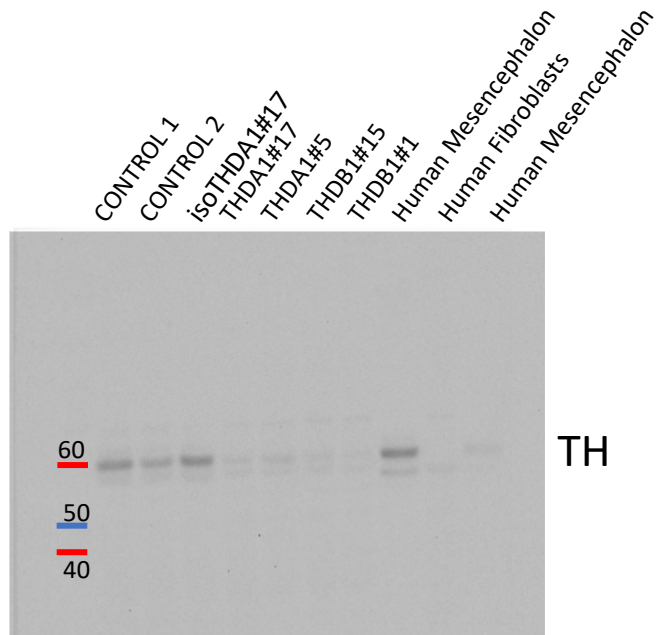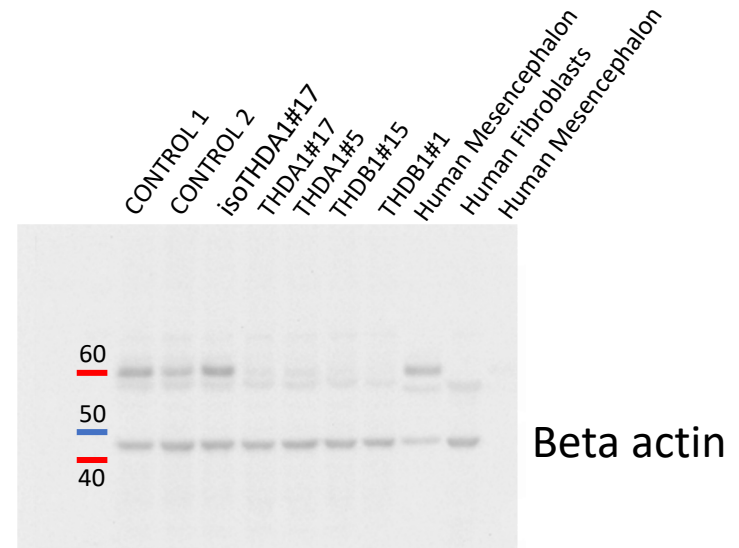

Supplement: Supplementary file 5 — Source Data for Figure 2 [file EMMM-15-e15847-s003.zip › Figure 2/2C.pdf]

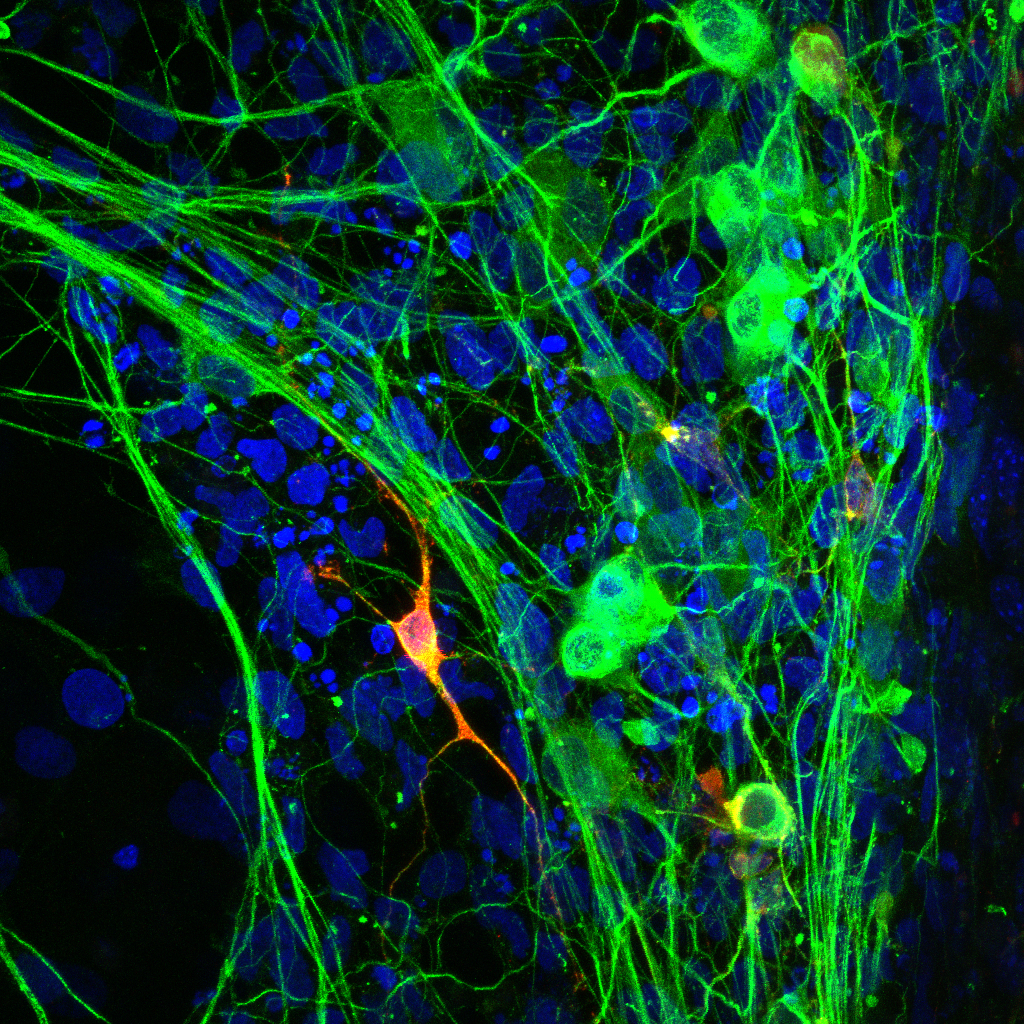

Supplement: Supplementary file 6 — Source Data for Figure 3 [file EMMM-15-e15847-s009.zip › Figure 3/3B/Fig 3B isoTHDA1#17.tif]

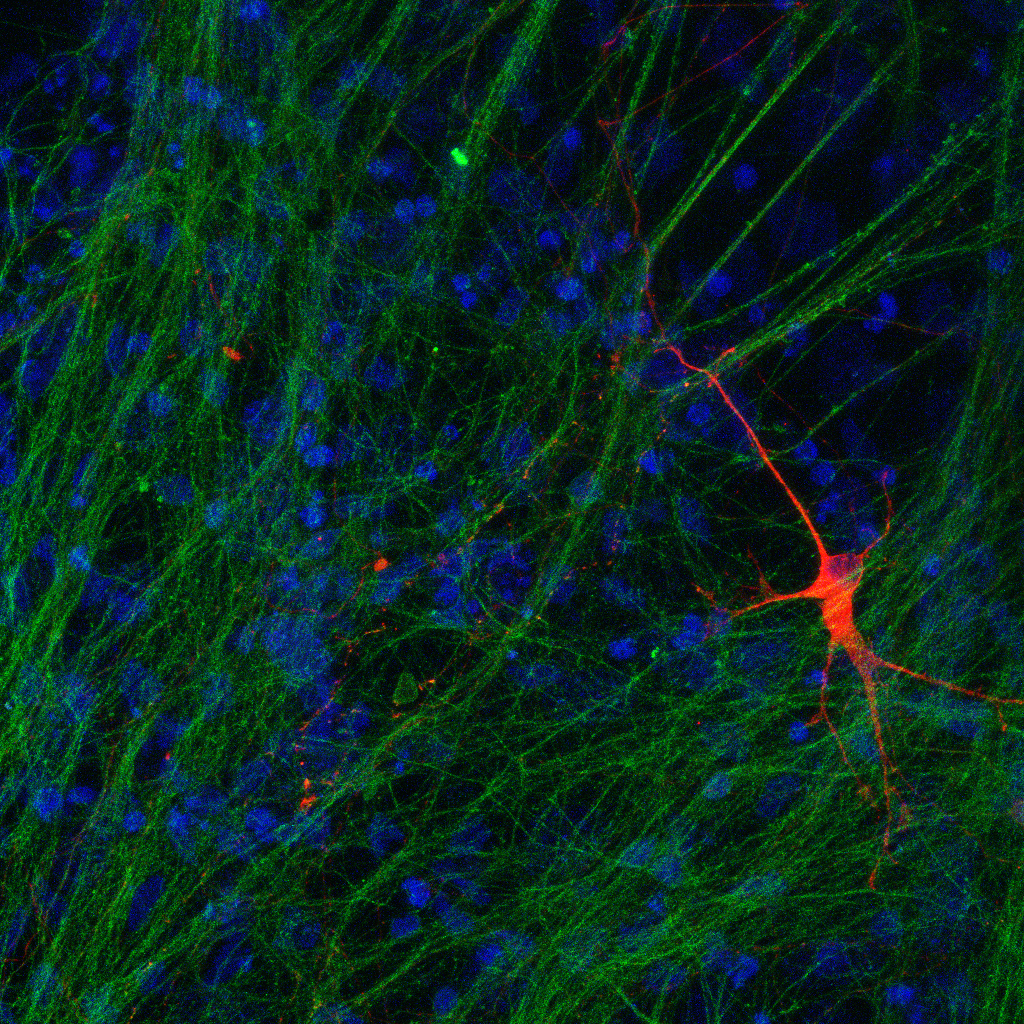

Supplement: Supplementary file 6 — Source Data for Figure 3 [file EMMM-15-e15847-s009.zip › Figure 3/3B/Fig 3B CONTROL 1.tif]

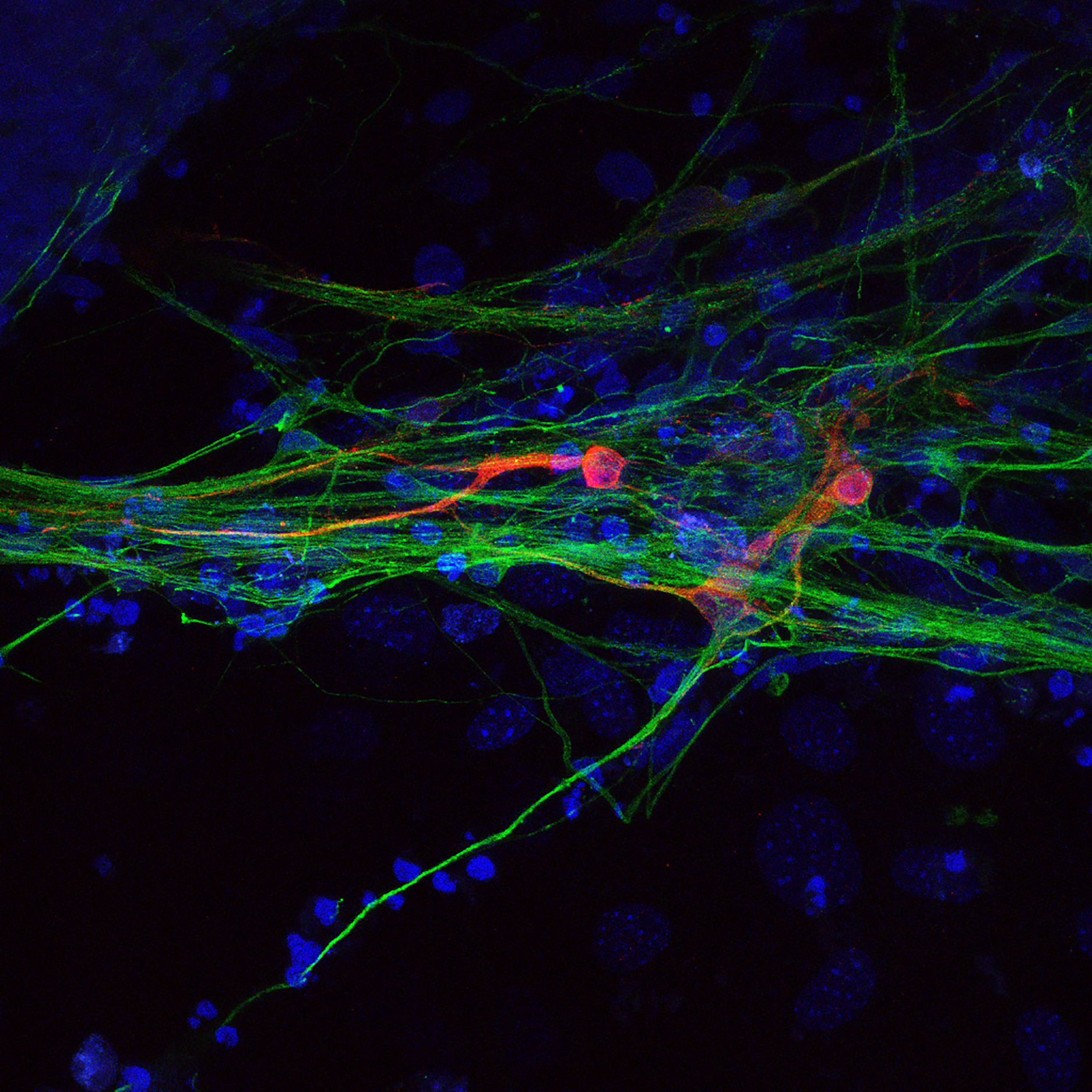

Supplement: Supplementary file 6 — Source Data for Figure 3 [file EMMM-15-e15847-s009.zip › Figure 3/3B/Fig 3B THDB1#15.tif]

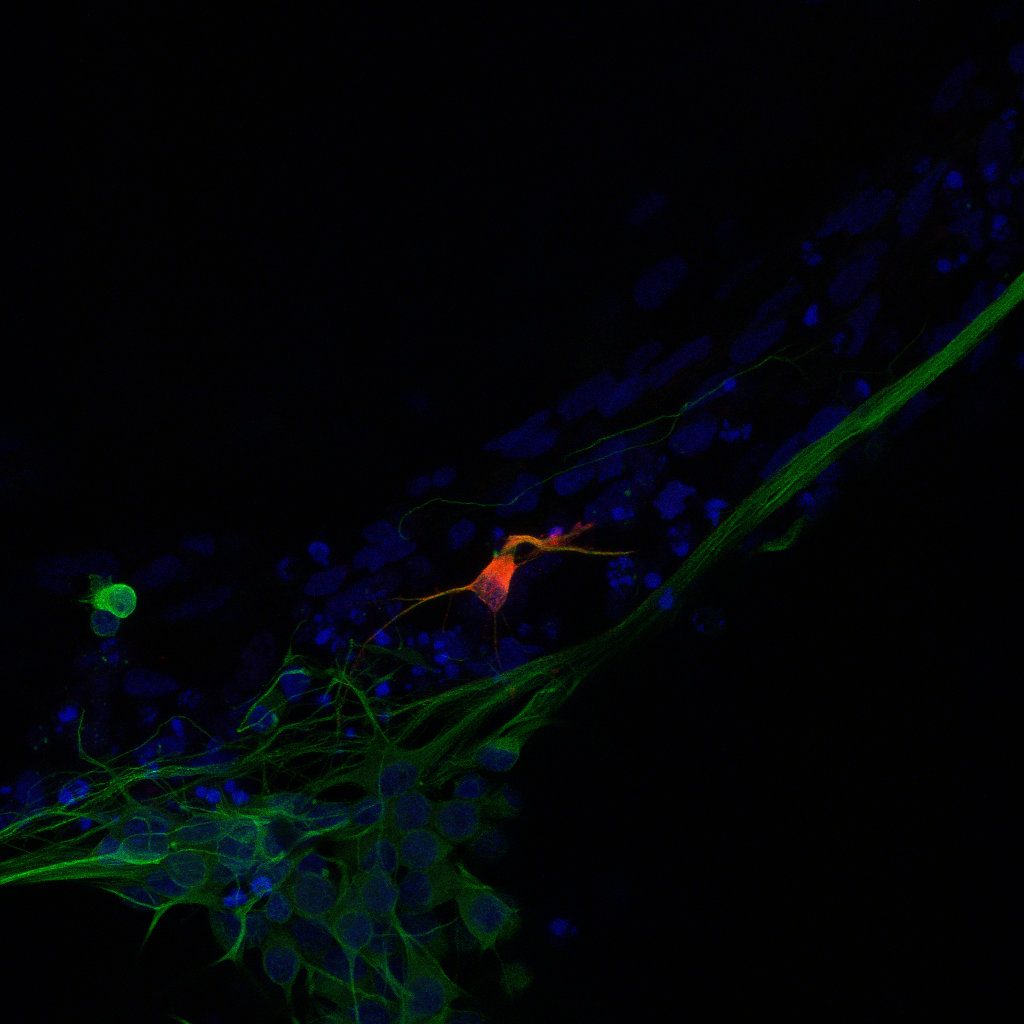

Supplement: Supplementary file 6 — Source Data for Figure 3 [file EMMM-15-e15847-s009.zip › Figure 3/3B/Fig 3B THDA1#17.tif]

## Uncropped WB Figure 4E: TH and actin

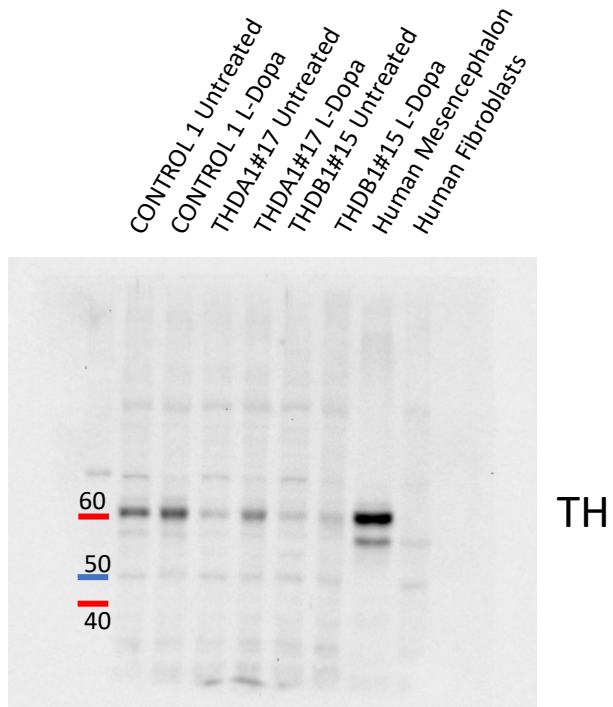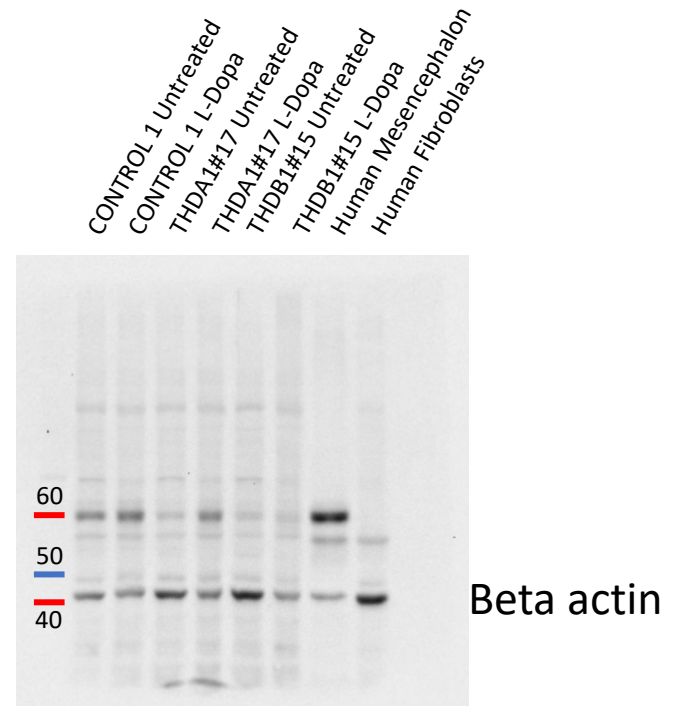

Supplement: Supplementary file 7 — Source Data for Figure 4 [file EMMM-15-e15847-s006.zip › Figure 4/4E.pdf]

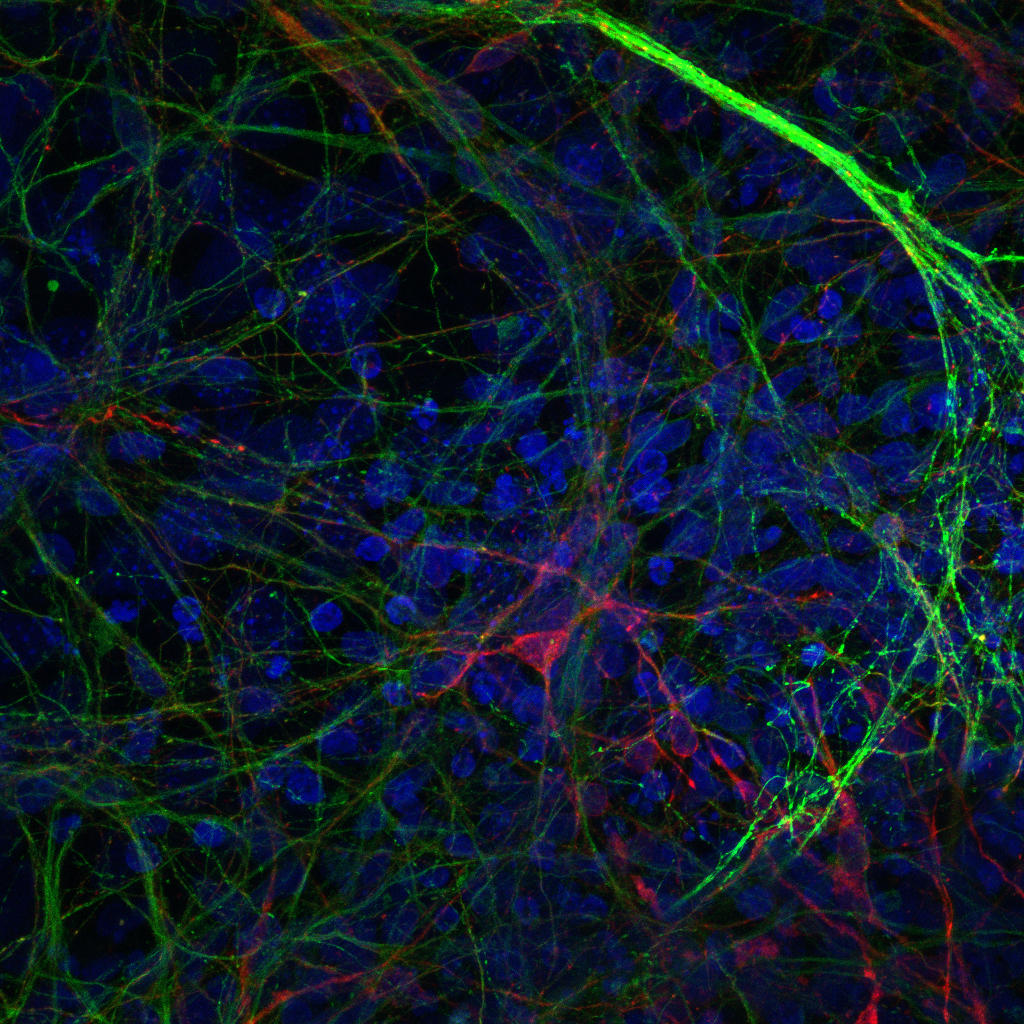

Supplement: Supplementary file 8 — Source Data for Figure 5 [file EMMM-15-e15847-s005.zip › Figure 5/5H/Fig 5H CONTROL 1 UNTREATED.tif]

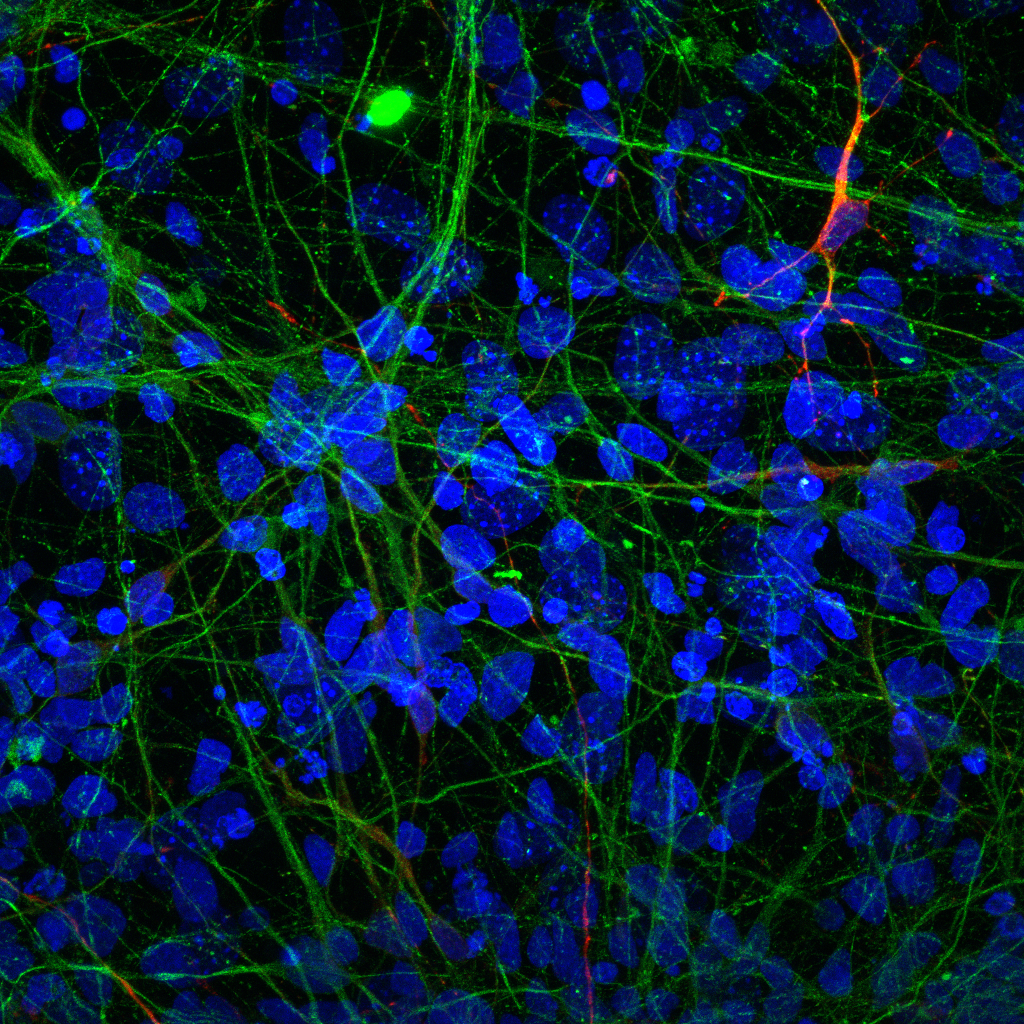

Supplement: Supplementary file 8 — Source Data for Figure 5 [file EMMM-15-e15847-s005.zip › Figure 5/5H/Fig 5H CONTROL 1 Early L-Dopa.tif]

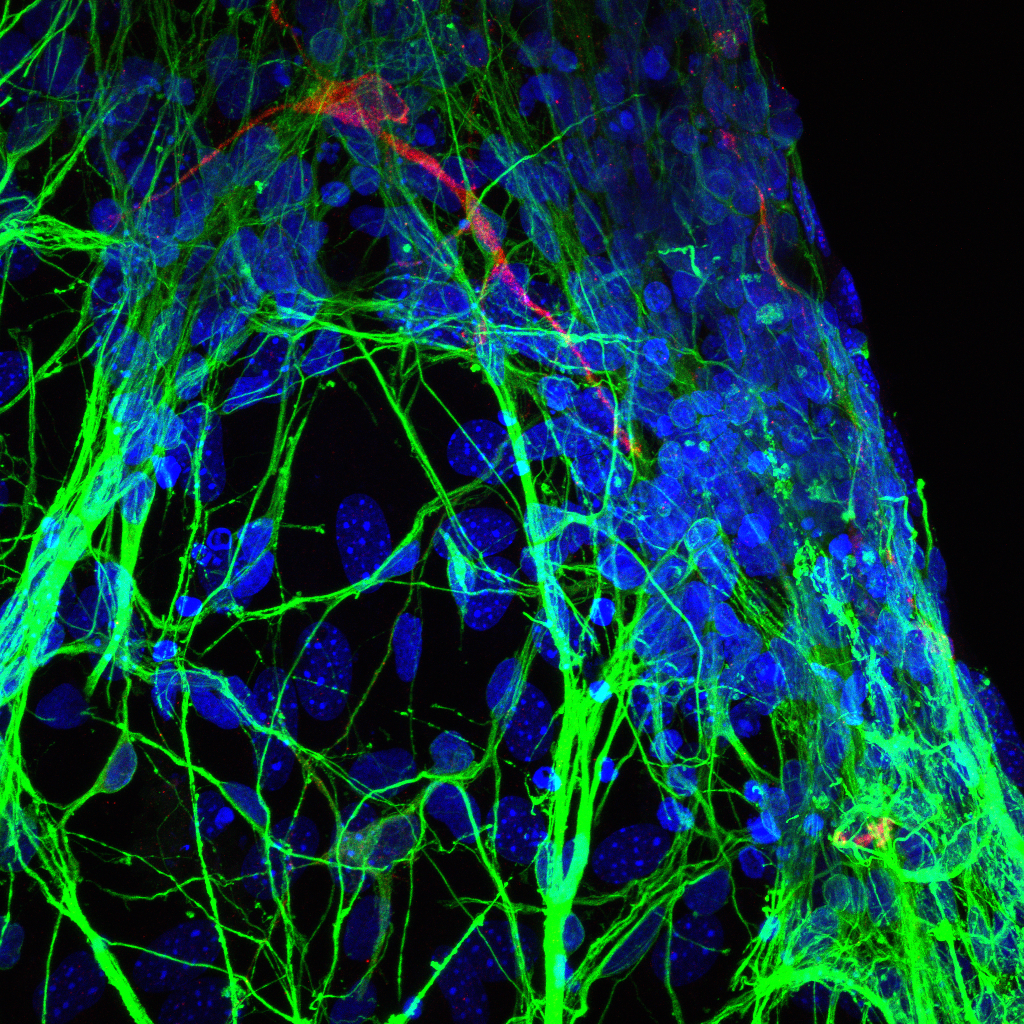

Supplement: Supplementary file 8 — Source Data for Figure 5 [file EMMM-15-e15847-s005.zip › Figure 5/5H/Fig 5H THDB1#15 Early L-Dopa.tif]

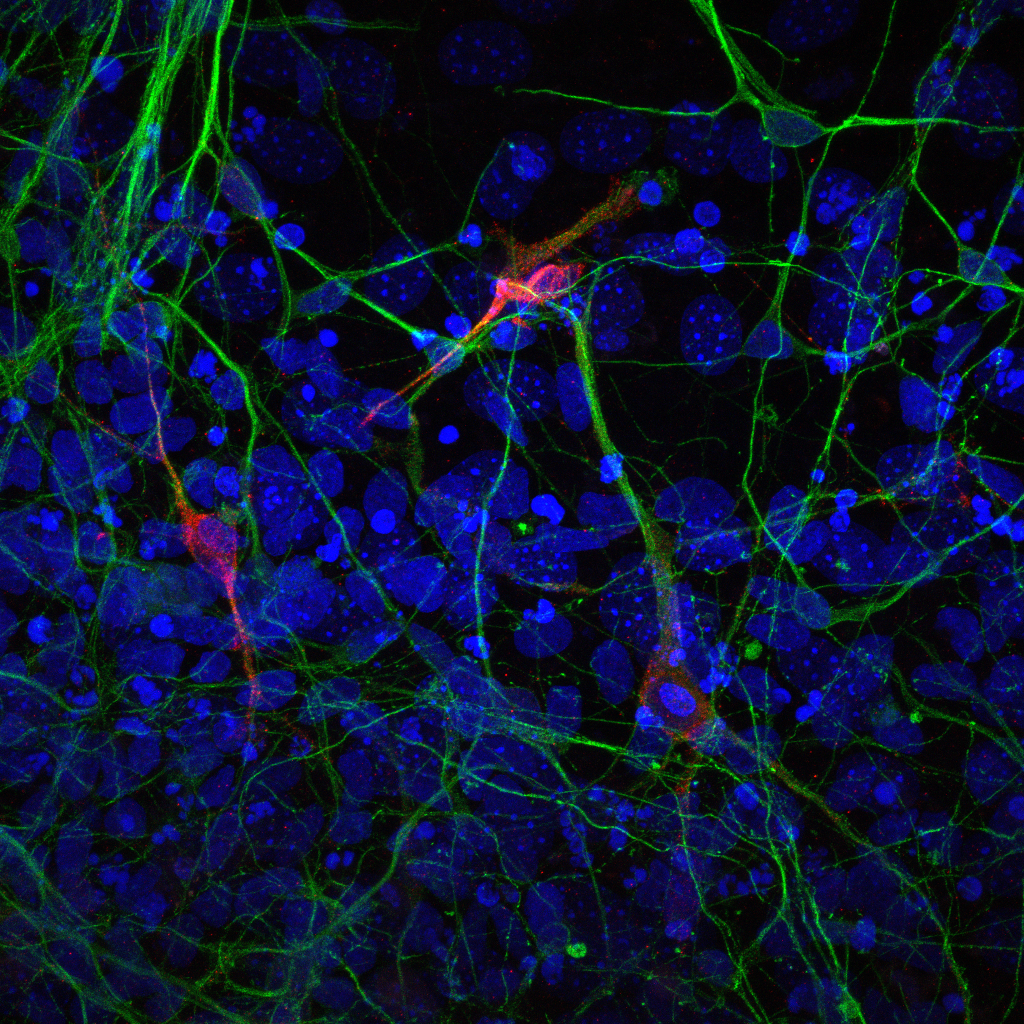

Supplement: Supplementary file 8 — Source Data for Figure 5 [file EMMM-15-e15847-s005.zip › Figure 5/5H/Fig 5H THDB1#15 UNTREATED.tif]
