# Supplementary material for: iPSC‐based modeling of THD recapitulates disease phenotypes and reveals neuronal malformation
Source: EMBO Mol Med. 2023 Feb 6;15(3):e15847. doi: 10.15252/emmm.202215847 (PMC9994475; doi:10.15252/emmm.202215847)
Supplement: Supplementary file 9 [file EMMM-15-e15847-s002.pdf]

EMBO Molecular Medicine

iPSC-based modeling of THD recapitulates disease phenotypes and reveals neuronal malformation

Alba Tristán Noguero, Irene Fernández-Carasa, Carles Calatayud, Cristina Bermejo-Casadesús, Meritxell Pons Espinal, Arianna Colini, Leticia Campa, Francesc Artigas, Analía Bortolozzi, Rosario Domingo-Jiménez, Salvador Ibáñez, Mercè Pineda, Rafael Artuch, Ángel Raya, Àngels García Cazorla, and Antonella Consiglio
DOI: 10.15252/emmm.202215847

Corresponding authors: Antonella Consiglio (consiglio@ub.edu) , Àngels García Cazorla (angeles.garcia@sjd.es)

Review Timeline:

Submission Date:	23rd Feb 22
Editorial Decision:	24th Mar 22
Revision Received:	25th Nov 22
Editorial Decision:	7th Dec 22
Revision Received:	28th Dec 22
Accepted:	10th Jan 23

Editor: Zeljko Durdevic

Transaction Report:

(Note: With the exception of the correction of typographical or spelling errors that could be a source of ambiguity, letters and reports are not edited. Depending on transfer agreements, referee reports obtained elsewhere may or may not be included in this compilation. Referee reports are anonymous unless the Referee chooses to sign their reports.)

24th Mar 2022

Dear Dr. Consiglio,

Thank you for the submission of your manuscript to EMBO Molecular Medicine. We have now received feedback from the two reviewers who agreed to evaluate your manuscript. As you will see from the reports below, the referees acknowledge the interest of the study but also raise important concerns that should be addressed in a major revision. Please make sure to pay particular attention to the grammar and syntax and I would recommend running the article by a native English speaker.

Further consideration of a revision that addresses reviewers' concerns in full will entail a second round of review. EMBO Molecular Medicine encourages a single round of revision only and therefore, acceptance or rejection of the manuscript will depend on the completeness of your responses included in the next, final version of the manuscript. For this reason, and to save you from any frustrations in the end, I would strongly advise against returning an incomplete revision.

We would welcome the submission of a revised version within three months for further consideration. Please let us know if you require longer to complete the revision.

I look forward to receiving your revised manuscript.

Yours sincerely,

Zeljko Durdevic

Zeljko Durdevic
Editor
EMBO Molecular Medicine

***** Reviewer's comments *****

Referee #1 (Comments on Novelty/Model System for Author):

It would be interesting to know the role of the second mutation since "the most frequent mutation (Arg233His) found in THD patients can cause a Type A or Type B phenotype." Right now genotype does not predict phenotype for reasons that remain unclear but would be valuable to explore and test experimentally.

Referee #1 (Remarks for Author):

This is both an interesting and well done study, in which iPSC lines are established from both mild (THDA) and severe (THDB) patients carrying the extremely rare disease TDH (tyrosine hydroxylase deficiency) involving mutations in the tyrosine hydroxylase gene. Upon differentiation of a DA phenotype in the progeny of these THD iPS lines, the Authors found a decrease TH+ DA neurons, TH protein, DA and its metabolites and many other DA markers (AADC, D1R, D1R, DAT, etc.) when compared to iPS lines from controls (healthy subject) or a mutation-repaired isogenic iPS line. In addition, the appearance of the TH+ DA neurons generated in TDH cultures is quite different showing less arborization than controls. Moreover, THDA DA neurons, but not THDB DA neurons, can be rescued by L-DOPA treatment in culture. Importantly, all of these changes are consistent both with animal models of the disease and what we know from THD patients. Thus, iPSC-based modeling of THD recapitulates disease phenotypes in culture and may be useful for studying the disease in the dish. Of potential clinical significance is the finding that L-DOPA treatment at an earlier stage of development (before neuronal differentiation when neural progenitors are forming in the EB) can rescue the phenotype even in THDB cells, suggesting that prenatal L-DOPA treatment could be ameliorative even in the severe form of the disease. However, since this is an extremely rare disease (only 80 known cases in the world), it is not clear if these findings will find their way to a clinic or remain an academic exercise.

Despite the commendable rigor used in the study, there remain some points requiring clarification:

1. If there is a loss of TH neurons with a concomitant decline in TH protein, DA, metabolites, why does TH mRNA not decrease (trend is even increasing). No explanation for this puzzling finding is offered on p.14 or in the Discussion. Also, if this is correct, why is TH mRNA not translated into TH protein?
2. Although th e focus of this article is on midbrain DA neurons, in THD, is the TH deficiency seen in all catecholamine nuclei in the brain (including all dopaminergic, noradrenergic and adrenergic nuclei)?
3. For the relative mRNA expression data shown in Fig2H and I and in Fig EV5 E and F, the controls are not set to 1. Why? What are the relative comparisons to? Usually, the control (even when normalized to a household protein) is set to 1 and all other variables compared to 1.
4. In Figure EV5 F, the DAT expression in THDA cultures without L-Dopa treatment is at control levels. This does not match what is shown in Fig 2I where the authors report "higher DAT expression levels in THDA neuronal cultures ($p<0.05$)" and explained that "This effect could be an adaptive behavior to use more efficiently the DA produced in TH-defective neurons by increasing DA uptake".
5. In Fig. 4, the validity of using TH immunocytochemistry to determine "proximodistal gradients" is questionable. I found this the weakest technical part of the paper. As fibers dive over or under other neurites or into the POLAM matrix or PA6 cells on the bottom of the dish, staining variations will always be observed, resulting from different access to antibodies. Most cultures would contain fields that look both like their controls and their experimentals in Fig. 4.
6. There is not parity in iPS lines. Control lines are both derived from males and THD lines derived from females. Also, the THDB line is from an adolescent- not age- matched as it says in the text. Please correct.

Stylistic Concerns:
1. Singular and plural is often misused. There are some typos, mis-spellings and atypical phrasing (ie. Using a postmortem brain tissue....Currently used THD cellular model include...). The manuscript would benefit from an English editing service.

2. On p. 13, please rephrase "....suggesting that the enzymatic defect may specifically affect the production of DAn" to "....suggesting that the TH mutation may specifically affect the production of DAn".
Enzymatic effect sounds like the problem is related to enzyme activity.

Referee #2 (Comments on Novelty/Model System for Author):

It is adequate to use patient-derived iPS cells and healthy and isogenic controls for dopaminergic neuron induction. However, it is inappropriate to evaluate improvement by drug treatment in the early developmental stages of this dopamine induction method, which involves gene induction into iPS cells.

Referee #2 (Remarks for Author):

Tristán-Noguero et al. generated iPS cells from two patients with THD, a very rare disease, and compared induced DA neurons with healthy controls and isogenic control cells to verify abnormalities in patient dopaminergic nerves. The data presented is very clear and well organized. It is very interesting that the use of iPS cell-derived DA neurons revealed phenotypes that would have been difficult to detect in previous pathological studies of autopsy brains. Unfortunately, however, the report only describes the phenotype, and does not explore the pathological mechanism of the disease. Also, some additional experiments would strengthen the work.

Major points
1. According to the cited paper, the induction method of DAn-enriched culture is to induce dopaminergic neuronal differentiation by exogenous expression of LMX1A directly in iPS cells using lentivirus. However, it seems unfair to only cite previous reports and not describe gene induction in the Materials and methods or figures, and not mention gene transfer in the discussion. The paper that should be cited as the induction method is Sanchez-Danes et al, 2011, https://doi.org/10.1089/hum.2011.054.

2. Figure 2. In the neurons from the patients, the percentage of TH-positive cells is decreased in IF and the amount of TH is decreased in WB, but TH mRNA is increased. Is this due to inefficient induction in the disease strain, or are TH-positive cells induced but TH degradation is enhanced, or is there a translation problem? I am not aware whether the number of DA-neurons are reduced in patient autopsy brains. Examination of other midbrain dopaminergic markers such as GIRK and PITX3, as well as single cell analysis, would help to address this concern.

3. Page14, Line17-20, and Discussions. The previous pathology report (Tristán-Noguero et al. 2016) evaluated only protein levels by Western blotting. Western blotting as well as qPCR is necessary for comparison with pathological findings.

4. Figure 4. Perhaps because the disease is so rare, these phenotypes are not common as an abnormality of DA neurons. Ensure that same results can also be obtained with another antibody that recognizes other epitopes of TH.

5. Page18, 19 and 20. As they point out in the Introduction, since the genotype-phenotype relationship is not clear in this disease, it is difficult to explain the differences in clinical symptoms and neuronal projections between THDA and THDB with only these two cases. To explain these differences by differences of D2DR expression, at least a validation of the protein levels is needed. Still, the qPCR results are in comparison to controls with different percentages of TH-positive cells, and if there are no significant differences between THDA and THDB, the assessment should be made more conservatively.

6. Figure5 and Figure6. As mentioned above, the DA neurons in this study were induced by a method different from normal development, in which the LMX1A gene is introduced into iPS cells. Especially for the results of Figure 6, it is necessary to show that some phenotypes are improved even in the DA neurons induced by a method that does not use gene transfer, since it claims an improvement by intervention in early development. In addition, there is a discrepancy between the results of the untreated samples in Fig6EF and Fig2FG.

Minor points
7. Page13, Line11, and Fig S1. This NPC was inducted using which method?
8. Page14, Line10. No data from isoTHDA#17 in Fig2E-G.

RE: EMM-2022-15847

Point-by-point response to the reviewers' comments

REFEREE #1

(Comments on Novelty/Model System for Author):
It would be interesting to know the role of the second mutation since "the most frequent mutation (Arg233His) found in THD patients can cause a Type A or Type B phenotype." Right now genotype does not predict phenotype for reasons that remain unclear but would be valuable to explore and test experimentally.
We fully agree with the reviewer's point regarding the interest on exploring and testing experimentally the causative effect of the second mutation on DAn derived from THD patients. We are now working on this aim for our follow up studies, that we hope will also reveal neurodevelopmental defects occurring in this disease.

(Remarks for Author):
This is both an interesting and well done study, in which iPSC lines are established from both mild (THDA) and severe (THDB) patients carrying the extremely rare disease TDH (tyrosine hydroxylase deficiency) involving mutations in the tyrosine hydroxylase gene. Upon differentiation of a DA phenotype in the progeny of these THD iPS lines, the Authors found a decrease TH+ DA neurons, TH protein, DA and its metabolites and many other DA markers (AADC, D1R, D1R, DAT, etc.) when compared to iPS lines from controls (healthy subject) or a mutation-repaired isogenic iPS line. In addition, the appearance of the TH+ DA neurons generated in TDH cultures is quite different showing less arborization than controls. Moreover, THDA DA neurons, but not THDB DA neurons, can be rescued by L-DOPA treatment in culture. Importantly, all of these changes are consistent both with animal models of the disease and what we know from THD patients. Thus, iPSC-based modeling of THD recapitulates disease phenotypes in culture and may be useful for studying the disease in the dish. Of potential clinical significance is the finding that L-DOPA treatment at an earlier stage of development (before neuronal differentiation when neural progenitors are forming in the EB) can rescue the phenotype even in THDB cells, suggesting that prenatal L-DOPA treatment could be ameliorative even in the severe form of the disease. However, since this is an extremely rare disease (only 80 known cases in the world), it is not clear if these findings will find their way to a clinic or remain an academic exercise.
We are very glad to read that the reviewer appreciated the interest of our studies. We should like to thank the reviewer for his/her comments and suggestions, and hope that the revised version of our manuscript, which incorporates new experimental evidence and text edits as per reviewer's recommendations, will satisfactorily address his/her concerns. Being involved at the forefront in the care of these patients and in the recruitment of new patients, we hope that our model will be used not only for understanding basic disease mechanisms but also for the study of new drugs that we hope will be used in the therapy of these patients.

Despite the commendable rigor used in the study, there remain some points requiring clarification:
1. If there is a loss of TH neurons with a concomitant decline in TH protein, DA, metabolites, why does TH mRNA not decrease (trend is even increasing). No explanation for this puzzling finding is offered on p.14 or in the Discussion. Also, if this is correct, why is TH mRNA not translated into TH protein?
The patients modeled in this study carry missense mutations in the *TH* gene that result in decreased TH enzyme only at the protein level, and not at the mRNA level. The same was observed by other groups in the THD mouse model in which neonate, juvenile, and adult THD mice had higher TH mRNA levels than their wildtype littermates (Korner et al 2015). We have now included additional text discussing this point in page 18 in the revised version of our manuscript.

2. Although the focus of this article is on midbrain DA neurons, in THD, is the TH deficiency seen in all catecholamine nuclei in the brain (including all dopaminergic, noradrenergic and adrenergic nuclei)?

The reviewer raises a very interesting point. Very few published studies report on the systematic characterization of catecholamine nuclei in the brain. However, some articles describe the presence of TH positive staining also in the brainstem nuclei corresponding to dopamine-, norepinephrine- and epinephrine-containing cells. Most TH-immune positive cells fill the lateral part of the reticular formation (Bucci et al, "Systematic Morphometry of Catecholamine Nuclei in the Brainstem" Front Neuroanat. 2017; 11: 98). Also, in the study of a fetal brain affected by a severe form of TH deficiency, TH detection was absent by Western blot studies in the mesencephalon and the pons of the patient (Tristán-Noguero et al, Metabolic Brain Disease, 31, 705-709 /2016). At any rate, to experimentally address the reviewer's point, we evaluated in a new set of experiments the presence of adrenergic and noradrenergic markers in the neuronal cultures of control, THDA, along with the isoTHDA counterpart, and THDB iPSC lines by RNA expression analysis. We examined the expression of DBH and NET for noradrenergic neurons and PNMT for adrenergic neurons. We included human locus coeruleus and mouse hypothalamus as positive controls for noradrenergic and adrenergic neurons, respectively. Our results indicate the presence of noradrenergic markers in our neuronal cultures although the levels were several orders of magnitude lower than the positive control (locus coeruleus). No adrenergic markers were detected in our neuronal cultures (see Fig. 1 for Reviewer below). Therefore, although it could be interesting to know whether TH deficiency affects other catecholamine nuclei, specific noradrenergic and adrenergic differentiation protocols would be needed to evaluate these specific neuronal populations.

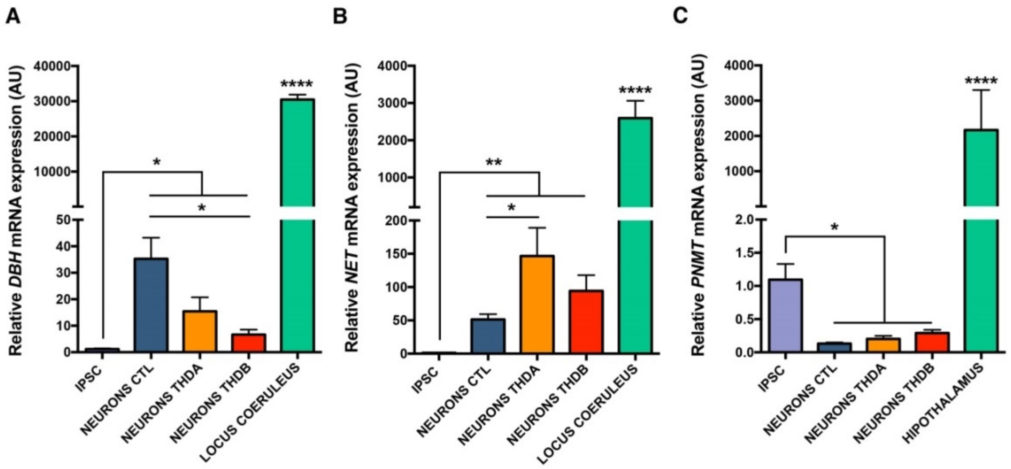

Fig 1 for reviewer. Relative mRNA expression of noradrenergic and adrenergic markers. A Relative *DBH* mRNA expression. **B** Relative *NET* mRNA expression. **C** Relative *PNMT* mRNA expression. Human locus coereleus was used as positive control for DBH and NET noradrenergic markers whereas mouse hypothalamus was used as positive control for PNMT adrenergic marker. iPSCs were used as negative control for both noradrenergic and adrenergic markers. N=3 experiments. Data are expressed as mean ± SEM. ANOVA or Kruskal-Wallis tests were performed for multiple comparisons. Unpaired two-tailed Student's t test or Mann-Whitney U test was used for pairwise comparisons. ****p<0.0001 **p<0.01 *p<0.05.

3. For the relative mRNA expression data shown in Fig2H and I and in Fig EV5 E and F, the controls are not set to 1. Why? What are the relative comparisons to? Usually, the control (even when normalized to a household protein) is set to 1 and all other variables compared to 1.

We apologize for the unclear presentation of these data in the original version of our manuscript. Following the reviewer's comment, we have now modified Fig. 2H-I and Fig. EV5 E-F in the revised submission to show the data as fold change compared to control.

4. In Figure EV5 F, the DAT expression in THDA cultures without L-Dopa treatment is at control levels. This does not match what is shown in Fig 2I where the authors report "higher DAT expression levels in THDA neuronal cultures (p<0.05)" and explained that "This effect could be an adaptive behavior to use more efficiently the DA produced in TH-defective neurons by increasing DA uptake".
We apologize for the unclear presentation of these data in the original version of our manuscript. To address this important point, in the revised version of our manuscript, we have now generated DAn from CTRL, THDA, THDB iPSCs and by including more experiments we validated the higher levels of DAT in untreated THDA1#17 line reaching significance (p-value<0,05) in addition to the already observed lower levels of AADC in both untreated THD cultures and the reduction in D2DR only in THDB1#15.

5. In Fig. 4, the validity of using TH immunocytochemistry to determine "proximodistal gradients" is questionable. I found this the weakest technical part of the paper. As fibers dive over or under other neurites or into the POLAM matrix or PA6 cells on the bottom of the dish, staining variations will always be observed, resulting from different access to antibodies. Most cultures would contain fields that look both like their controls and their experiments in Fig. 4.
We appreciate the reviewer´s concern and agree with the reviewer's point that this result could be confounded by intrinsic variations in the cell culture, and therefore we have decided to remove this information in the revised version of our manuscript. We believe that excluding these data and figure does not affect the main message of our study and should like to thank the reviewer for pointing this out.

6. There is not parity in iPS lines. Control lines are both derived from males and THD lines derived from females. Also, the THDB line is from an adolescent- not age- matched as it says in the text. Please correct.
We thank the reviewer for noticing this mistake, which has been corrected in the revised version of our manuscript.

Stylistic Concerns:
1. Singular and plural is often misused. There are some typos, mis-spellings and atypical phrasing (ie. Using a postmortem brain tissue....Currently used THD cellular model include...). The manuscript would benefit from an English editing service.
We apologize for the mistakes. The text in the revised version of our manuscript has now been proofread by a native English speaker.

2. On p. 13, please rephrase "....suggesting that the enzymatic defect may specifically affect the production of DAn" to "....suggesting that the TH mutation may specifically affect the production of DAn".
Enzymatic effect sounds like the problem is related to enzyme activity.
We appreciate the reviewer's suggestion, which we have addressed in the revised version of our manuscript by editing the sentence (page 14 of the revised manuscript).

REFEREE #2

(Comments on Novelty/Model System for Author):
It is adequate to use patient-derived iPS cells and healthy and isogenic controls for dopaminergic neuron induction. However, it is inappropriate to evaluate improvement by drug treatment in the early developmental stages of this dopamine induction method, which involves gene induction into iPS cells.
We thank the reviewer for allowing us to clarify this important point as we fully agree that exogenous expression of LMX1A could affect the interpretation of our results. The protocol we used did not include the forced expression of LMX1A. We apologize for having omitted this important piece of information in the original version of our manuscript.

(Remarks for Author):
Tristán-Noguero et al. generated iPS cells from two patients with THD, a very rare disease, and compared induced DA neurons with healthy controls and isogenic control cells to verify abnormalities in patient dopaminergic nerves. The data presented is very clear and well organized. It is very interesting that the use of iPS cell-derived DA neurons revealed phenotypes that would have been difficult to detect in previous pathological studies of autopsy brains. Unfortunately, however, the report only describes the phenotype, and does not explore the pathological mechanism of the disease. Also, some additional experiments would strengthen the work.
We are very glad to read that the reviewer appreciated the interest of our studies. We should like to thank the reviewer for his/her comments and suggestions, and hope that the revised version of our manuscript, which incorporates new experimental evidence and text edits as per reviewer's recommendations, will satisfactorily address his/her concerns.

Major points
Comment 1. According to the cited paper, the induction method of DAn-enriched culture is to induce dopaminergic neuronal differentiation by exogenous expression of LMX1A directly in iPS cells using lentivirus. However, it seems unfair to only cite previous reports and not describe gene induction in the Materials and methods or figures, and not mention gene transfer in the discussion. The paper that should be cited as the induction method is Sanchez-Danes et al, 2011, https://doi.org/10.1089/hum.2011.054.
We completely understand the reviewer´s concern and apologize for the confusion created by omitting the crucial information that LMX1A overexpression was not used in our experiments. The protocol we used followed the 3-step protocol described in the paper referred to by the reviewer (Sanchez-Danes et al, 2011), but did not include LMX1A induction, since obtaining ventral midbrain DAn of A9 subtype was not critical for modeling THD. We have now included this information and cited the appropriate reference in the revised version of our manuscript (page 7 of the revised manuscript).

Comment 2. Figure 2. In the neurons from the patients, the percentage of TH-positive cells is decreased in IF and the amount of TH is decreased in WB, but TH mRNA is increased. Is this due to inefficient induction in the disease strain, or are TH-positive cells induced but TH degradation is enhanced, or is there a translation problem? I am not aware whether the number of DA-neurons are reduced in patient autopsy brains. Examination of other midbrain dopaminergic markers such as GIRK and PITX3, as well as single cell analysis, would help to address this concern.
The TH mutations studied in this work are missense mutations that result in altered TH expression only at the protein level but that, in principle, do not affect mRNA transcription. Similar results have been observed in newborn, juvenile and adult THD mouse model, in which higher TH mRNA levels were detected when compared to wildtype littermates (Korner et al 2015). Moreover, since dopamine behaves as a growth factor, especially during neurodevelopment, low levels of this neurotransmitter due to TH deficiency may impair neuronal processes such as synapse formation

and dendritogenesis (as it was found in Tristán-Noguero et al 2016). The increased TH mRNA expression in neurons from THD patients and mouse models could therefore be explained by a homeostatic reaction to compensate dopaminergic depletion due to the genetic mutation leading to TH deficiency. We have now included this point in the discussion section (page 18 of the revised manuscript).

Regarding the number of DAn in the postmortem brains of THD patients, it has been reported in a 16-week-old fetus brain autopsy of THD that markers of total neuronal volume were preserved whereas TH expression was reduced. Unfortunately, it was not possible to perform IHC studies to specifically assess the number of DA nuclei (Tristán-Noguero et al, Metabolic Brain Disease, 31, 705-709, 2016). This finding is also in agreement with DAT studies in THD patients, who show no signs of dopaminergic neuron loss. As suggested by the reviewer, we have now examined the expression of other midbrain DA markers by qRT-PCR including GIRK, PITX3, FOXA2, LMX1A, EN1 and NURR1, and found that all of them are highly expressed in our cell cultures. We have used ventral midbrain dopaminergic neurons (vmDAn) as positive control and iPSCs as negative control. We have included these data in Appendix Fig S2 of the revised manuscript.

Comment 3. Page14, Line17-20, and Discussions. The previous pathology report (Tristán-Noguero et al. 2016) evaluated only protein levels by Western blotting. Western blotting as well as qPCR is necessary for comparison with pathological findings.
The reviewer's point is well taken. To address it, we have performed additional DAn differentiation experiments and validated the qPCR results by protein expression analysis. Specifically, we have validated the results by Western blot for AADC and D2DR proteins, and found a significant reduction of both proteins in THD neuronal cultures as compared to control ones, thus confirming the impairment on DAn production in both THD neuronal cultures. These results have been included in the revised manuscript (page 14 and Fig EV2).

Comment 4. Figure 4. Perhaps because the disease is so rare, these phenotypes are not common as an abnormality of DA neurons. Ensure that same results can also be obtained with another antibody that recognizes other epitopes of TH.
We thank the reviewer for the experimental suggestion. We performed additional differentiation experiments and used two other antibodies against different TH epitopes, but the results obtained were inconclusive. For this reason, and also following the advice of Reviewer #1 (see response to his/her comment # 5 above), we have decided to remove this information in the revised version of our manuscript. We believe that excluding these data and figure does not affect the main message of our study.

Minor comments:
Comment 5. Page18, 19 and 20. As they point out in the Introduction, since the genotype-phenotype relationship is not clear in this disease, it is difficult to explain the differences in clinical symptoms and neuronal projections between THDA and THDB with only these two cases. To explain these differences by differences of D2DR expression, at least a validation of the protein levels is needed. Still, the qPCR results are in comparison to controls with different percentages of TH-positive cells, and if there are no significant differences between THDA and THDB, the assessment should be made more conservatively.
We fully agree with the reviewer's point. We have included new experiments and validated at protein levels the expression differences in D2DR. Our results indicate that D2DR is significantly reduced in both THD neuronal cultures as compared to control ones, thus confirming the impairment on DAn production in both THD neuronal cultures. These results have been included in the revised manuscript (page 14 and Fig. EV2).

Comment6. Figure5 and Figure6. As mentioned above, the DA neurons in this study were induced by a method different from normal development, in which the LMX1A gene is introduced into iPS cells. Especially, for the results of Figure 6, it is necessary to show that some phenotypes are improved even in the DA neurons induced by a method that does not use gene transfer, since it claims an improvement by intervention in early development. In addition, there is a discrepancy between the results of the untreated samples in Fig6EF and Fig2FG.

As stated above in our response to reviewer's comment #1, we understand the reviewer's concern and apologize for having omitted in the original submission the critical information that LMX1A overexpression was not used in these studies. Again, we have now corrected this mistake in the revised version of our manuscript and clarified the actual protocol used (without forced expression of LMX1A). Regarding the apparent discrepancy in the results shown in Fig 6 when compared to Fig 2 in the previous version of the manuscript we believe it was due to a lower number of biological replicates and therefore a reduced statistic power.

To address this important point, in the revised version of our manuscript, we have now generated and treated with L-Dopa, DAn from CTRL and THDB iPSCs and by including these experiments we confirmed the lower levels of intracellular DA (p-value<0.01), extracellular DA and DOPAC (p-value<0.05) in untreated THDB1#15, compared to the untreated controls. New results are now included in the figure (please note that the previous Fig. 6 is now Fig 5).

Minor points

7. Page13, Line11, and Fig S1. This NPC was inducted using which method?

We thank the reviewer for giving the opportunity to clarify the point. New information regarding the method and reference is added now in page 8 line 4.

8. Page14, Line10. No data from isoTHDA#17 in Fig2E-G.

Data from isoTHDA#17 was shown in Fig. 2E-G in the original version of the manuscript, in column CT combined with the results from CONTROL 1 and CONTROL 2. This information is now explicitly stated now in the legend to Fig. 2 in the revised version of our manuscript. The reason for combining results obtained with these three cell lines is that all represent control conditions for THD (healthy subjects and isogenic control). We are separating the results in Fig. 2 for reviewer shown below, to illustrate that the levels of Dopamine and DOPAC data in isoTHDA#17 are indeed comparable to those of CONTROL 1 and CONTROL 2. For the figures shown in the manuscript, we combined the data from the three control lines to make the statistical analyses more robust.

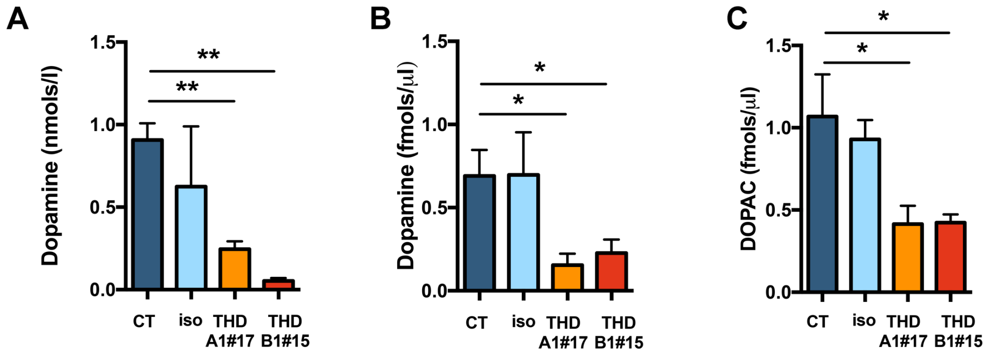

Fig 2 for reviewer. Dopamine and DOPAC measurements. A ELISA quantification of intracellular dopamine levels (nmols/l) in cultures of controls from healthy subjects (CT, including CONTROL 1 and CONTROL 2), isoTHDA1#17, THDA1#17 and THDB1#15. **B** HPLC quantification of dopamine levels (fmols/μl) in cultures of controls from healthy subjects (CT, including CONTROL 1 and CONTROL 2), isoTHDA1#17, THDA1#17 and THDB1#15. **C** HPLC quantification of DOPAC levels (fmols/μl) in cultures of controls from healthy subjects (CT, including CONTROL 1 and CONTROL 2), isoTHDA1#17, THDA1#17 and THDB1#15. Data are expressed as mean ± SEM. ANOVA or Kruskal-Wallis tests were performed for multiple comparisons. Unpaired two-tailed Student's t test or Mann-Whitney U test was used for pairwise comparisons. **p<0.01 *p<0.05.

7th Dec 2022

Dear Dr. Consiglio,

Thank you for the submission of your revised manuscript to EMBO Molecular Medicine. I am pleased to inform you that we will be able to accept your manuscript pending the following final amendments:

1) In the main manuscript file, please do the following:
- Correct/answer the track changes suggested by our data editors by working from the attached document.
- Remove font colour, also in the "Appendix".
- Move M&M section after Discussion.
- In M&M, please include statement that in addition to WMA Declaration of Helsinki the experiments also conformed the Department of Health and Human Services Belmont Report.
- Please rename "Competing Interest" to "Disclosure Statement & Competing Interests". We updated our journal's competing interests policy in January 2022 and request authors to consider both actual and perceived competing interests. Please review the policy https://www.embopress.org/competing-interests and update your competing interests if necessary.
- Author contributions: Please remove it from the manuscript and specify author contributions in our submission system. CRediT has replaced the traditional author contributions section because it offers a systematic machine-readable author contributions format that allows for more effective research assessment. You are encouraged to use the free text boxes beneath each contributing author's name to add specific details on the author's contribution. More information is available in our guide to authors:
https://www.embopress.org/page/journal/17574684/authorguide#authorshipguidelines
2) Funding: Please make sure that information about all sources of funding are complete in both our submission system and in the manuscript.
3) Synopsis: Every published paper now includes a 'Synopsis' to further enhance discoverability. Synopses are displayed on the journal webpage and are freely accessible to all readers. They include separate synopsis image and synopsis text.
- Synopsis image: Please resize the visual abstract to 550 px-wide x (250-400)-px high and submit as a high-resolution .jpeg file.
- Synopsis text: Please provide a short standfirst (maximum of 300 characters, including space) as well as 2-5 one sentence bullet points that summarise the paper as a .doc file. Please write the bullet points to summarise the key NEW findings. They should be designed to be complementary to the abstract - i.e. not repeat the same text. We encourage inclusion of key acronyms and quantitative information (maximum of 30 words / bullet point). Please use the passive voice.
- Please check your synopsis text and image before submission with your revised manuscript. Please be aware that in the proof stage minor corrections only are allowed (e.g., typos).
4) Source data: We encourage you to include the source data for figure panels that show essential data. Numerical data should be provided as individual .xls or .csv files (including a tab describing the data). For blots or microscopy, uncropped images should be submitted (using a zip archive if multiple images need to be supplied for one panel). For the main figures please upload one file per figure, while all EV figures should be zipped and uploaded as one file. Please check "Author Guidelines" for more information.
5) As part of the EMBO Publications transparent editorial process initiative (see our Editorial at http://embomolmed.embopress.org/content/2/9/329), EMBO Molecular Medicine will publish online a Review Process File (RPF) to accompany accepted manuscripts. This file will be published in conjunction with your paper and will include the anonymous referee reports, your point-by-point response and all pertinent correspondence relating to the manuscript. Let us know whether you agree with the publication of the RPF and as here, if you want to remove or not any figures from it prior to publication. Please note that the Authors checklist will be published at the end of the RPF.
6) Please provide a point-by-point letter INCLUDING my comments as well as the reviewer's reports and your detailed responses (as Word file).

I look forward to reading a new revised version of your manuscript as soon as possible.

Yours sincerely,

Zeljko Durdevic

Zeljko Durdevic
Editor
EMBO Molecular Medicine

***** Reviewer's comments *****

Referee #1 (Comments on Novelty/Model System for Author):

Excellent manuscript- interesting study done with rigor.

Referee #1 (Remarks for Author):

The Authors have addressed each and every concern in a very thorough fashion.

The authors addressed the minor editorial issues.

We are pleased to inform you that your manuscript is accepted for publication and is now being sent to our publisher to be included in the next available issue of EMBO Molecular Medicine.

EMBO Press Author Checklist

Corresponding Author Name: Antonella Consiglio	
Journal Submitted to: EMBO Molecular Medicine	
Manuscript Number: EMM-2022-15847	

USEFUL LINKS FOR COMPLETING THIS FORM
The EMBO Journal - Author Guidelines
EMBO Reports - Author Guidelines
Molecular Systems Biology - Author Guidelines
EMBO Molecular Medicine - Author Guidelines

Reporting Checklist for Life Science Articles (updated January 2022)
This checklist is adapted from Materials Design Analysis Reporting (MDAR) Checklist for Authors. MDAR establishes a minimum set of requirements in transparent reporting in the life sciences (see Statement of Task: 10.31222/osf.io/9sm4x). Please follow the journal's guidelines in preparing your manuscript.
Please note that a copy of this checklist will be published alongside your article.

Abridged guidelines for figures
1. Data
The data shown in figures should satisfy the following conditions:
- the data were obtained and processed according to the field's best practice and are presented to reflect the results of the experiments in an accurate and unbiased manner.
- ideally, figure panels should include only measurements that are directly comparable to each other and obtained with the same assay.
- plots include clearly labeled error bars for independent experiments and sample sizes. Unless justified, error bars should not be shown for technical replicates.
- if n<5, the individual data points from each experiment should be plotted. Any statistical test employed should be justified.
- Source Data should be included to report the data underlying figures according to the guidelines set out in the authorship guidelines on Data Presentation.

2. Captions
Each figure caption should contain the following information, for each panel where they are relevant:
- a specification of the experimental system investigated (eg cell line, species name).
- the assay(s) and method(s) used to carry out the reported observations and measurements.
- an explicit mention of the biological and chemical entity(ies) that are being measured.
- an explicit mention of the biological and chemical entity(ies) that are altered/varied/perturbed in a controlled manner.
- the exact sample size (n) for each experimental group/condition, given as a number, not a range;
- a description of the sample collection allowing the reader to understand whether the samples represent technical or biological replicates (including how many animals, litters, cultures, etc.).
- a statement of how many times the experiment shown was independently replicated in the laboratory.
- definitions of statistical methods and measures:
  - common tests, such as t-test (please specify whether paired vs. unpaired), simple χ2 tests, Wilcoxon and Mann-Whitney tests, can be unambiguously identified by name only, but more complex techniques should be described in the methods section;
  - are tests one-sided or two-sided?
  - are there adjustments for multiple comparisons?
  - exact statistical test results, e.g., P values = x but not P values < x;
  - definition of 'center values' as median or average;
  - definition of error bars as s.d. or s.e.m.

Please complete ALL of the questions below.
Select "Not Applicable" only when the requested information is not relevant for your study.

Materials

Newly Created Materials	Information included in the manuscript?	In which section is the information available? (Reagents and Tools Table, Materials and Methods, Figures, Data Availability Section)
New materials and reagents need to be available; do any restrictions apply?	Yes	Materials and Methods

Antibodies	Information included in the manuscript?	In which section is the information available? (Reagents and Tools Table, Materials and Methods, Figures, Data Availability Section)
For **antibodies** provide the following information:<br>- Commercial antibodies: RRID (if possible) or supplier name, catalogue number and or/clone number<br>- Non-commercial: RRID or citation	Yes	Materials and Methods

DNA and RNA sequences	Information included in the manuscript?	In which section is the information available? (Reagents and Tools Table, Materials and Methods, Figures, Data Availability Section)
**Short novel DNA or RNA including primers, probes**: provide the sequences.	Yes	Materials and Methods

Cell materials	Information included in the manuscript?	In which section is the information available? (Reagents and Tools Table, Materials and Methods, Figures, Data Availability Section)
**Cell lines:** Provide species information, strain. Provide accession number in repository **OR** supplier name, catalog number, clone number, and/**OR** RRID.	Yes	Materials and Methods
**Primary cultures:** Provide species, strain, sex of origin, genetic modification status.	Not Applicable	
Report if the cell lines were recently **authenticated** (e.g., by STR profiling) and tested for mycoplasma contamination.	Yes	Materials and Methods

Experimental animals	Information included in the manuscript?	In which section is the information available? (Reagents and Tools Table, Materials and Methods, Figures, Data Availability Section)
**Laboratory animals or Model organisms:** Provide species, strain, sex, age, genetic modification status. Provide accession number in repository **OR** supplier name, catalog number, clone number, **OR** RRID.	Not Applicable	
**Animal observed in or captured from the field:** Provide species, sex, and age where possible.	Not Applicable	
Please detail **housing and husbandry conditions**.	Not Applicable	

Plants and microbes	Information included in the manuscript?	In which section is the information available? (Reagents and Tools Table, Materials and Methods, Figures, Data Availability Section)
**Plants:** provide species and strain, ecotype and cultivar where relevant, unique accession number if available, and source (including location for collected wild specimens).	Not Applicable	
**Microbes:** provide species and strain, unique accession number if available, and source.	Not Applicable	

Human research participants	Information included in the manuscript?	In which section is the information available? (Reagents and Tools Table, Materials and Methods, Figures, Data Availability Section)
If collected and within the bounds of privacy constraints report on age, sex and gender or ethnicity for all study participants.	Not Applicable	

Core facilities	Information included in the manuscript?	In which section is the information available? (Reagents and Tools Table, Materials and Methods, Figures, Data Availability Section)
If your work benefited from core facilities, was their service mentioned in the acknowledgments section?	Yes	Materials and Methods

Design

Study protocol	Information included in the manuscript?	In which section is the information available? (Reagents and Tools Table, Materials and Methods, Figures, Data Availability Section)
If study protocol has been **pre-registered, provide DOI in the manuscript**. For clinical trials, provide the trial registration number **OR** cite DOI.	Not Applicable	
Report the **clinical trial registration number** (at ClinicalTrials.gov or equivalent), where applicable.	Not Applicable	

Laboratory protocol	Information included in the manuscript?	In which section is the information available? (Reagents and Tools Table, Materials and Methods, Figures, Data Availability Section)
Provide DOI OR other citation details if **external detailed step-by-step protocols** are available.	Yes	Materials and Methods

Experimental study design and statistics	Information included in the manuscript?	In which section is the information available? (Reagents and Tools Table, Materials and Methods, Figures, Data Availability Section)
Include a statement about **sample size** estimate even if no statistical methods were used.	Yes	Materials and Methods
Were any steps taken to minimize the effects of subjective bias when allocating animals/samples to treatment (e.g. **randomization procedure**)? If yes, have they been described?	Yes	Materials and Methods
Include a statement about **blinding** even if no blinding was done.	Yes	Materials and Methods
Describe **inclusion/exclusion criteria** if samples or animals were excluded from the analysis. Were the criteria pre-established?<br><br>If sample or data points were omitted from analysis, report if this was due to attrition or intentional exclusion and provide justification.	Yes	Materials and Methods
For every figure, are **statistical tests** justified as appropriate? Do the data meet the assumptions of the tests (e.g., normal distribution)? Describe any methods used to assess it. Is there an estimate of variation within each group of data? Is the variance similar between the groups that are being statistically compared?	Yes	Materials and Methods; Figure legends

Sample definition and in-laboratory replication	Information included in the manuscript?	In which section is the information available? (Reagents and Tools Table, Materials and Methods, Figures, Data Availability Section)
In the figure legends: state number of times the experiment was **replicated** in laboratory.	Yes	Figure legends
In the figure legends: define whether data describe **technical or biological replicates**.	Yes	Figure legends

Ethics

Ethics	Information included in the manuscript?	In which section is the information available? (Reagents and Tools Table, Materials and Methods, Figures, Data Availability Section)
Studies involving **human participants**: State details of **authority granting ethics approval** (IRB or equivalent committee(s), provide reference number for approval.	Yes	Materials and Methods
Studies involving **human participants**: Include a statement confirming that **informed consent** was obtained from all subjects and that the experiments conformed to the principles set out in the WMA Declaration of Helsinki and the Department of Health and Human Services Belmont Report.	Yes	Materials and Methods
Studies involving **human participants:** For publication of **patient photos**, include a statement confirming that consent to publish was obtained.	Not Applicable	
Studies involving experimental **animals**: State details of **authority granting ethics approva**l (IRB or equivalent committee(s), provide reference number for approval. Include a statement of compliance with ethical regulations.	Not Applicable	
Studies involving **specimen and field samples:** State if relevant **permits** obtained, provide details of authority approving study; if none were required, explain why.	Yes	Materials and Methods

Dual Use Research of Concern (DURC)	Information included in the manuscript?	In which section is the information available? (Reagents and Tools Table, Materials and Methods, Figures, Data Availability Section)
Could your study fall under dual use research restrictions? Please check biosecurity documents and list of **select agents and toxins** (CDC): https://www.selectagents.gov/sat/list.htm	Not Applicable	
If you used a select agent, is the security level of the lab appropriate and reported in the manuscript?	Not Applicable	
If a study is subject to dual use research of concern regulations, is the name of the **authority granting approval and reference number** for the regulatory approval provided in the manuscript?	Not Applicable	

Reporting

The MDAR framework recommends adoption of discipline-specific guidelines, established and endorsed through community initiatives. Journals have their own policy about requiring specific guidelines and recommendations to complement MDAR.

Adherence to community standards	Information included in the manuscript?	In which section is the information available? (Reagents and Tools Table, Materials and Methods, Figures, Data Availability Section)
State if relevant guidelines or checklists (e.g., **ICMJE, MIBBI, ARRIVE, PRISMA**) have been followed or provided.	Yes	Author contribution
For **tumor marker prognostic studies,** we recommend that you follow the **REMARK** reporting guidelines (see link list at top right). See author guidelines, under 'Reporting Guidelines'. Please confirm you have followed these guidelines.	Not Applicable	
For **phase II and III randomized controlled trials**, please refer to the **CONSORT** flow diagram (see link list at top right) and submit the CONSORT checklist (see link list at top right) with your submission. See author guidelines, under 'Reporting Guidelines'. Please confirm you have submitted this list.	Not Applicable	

Data Availability

Data availability	Information included in the manuscript?	In which section is the information available? (Reagents and Tools Table, Materials and Methods, Figures, Data Availability Section)
Have **primary datasets** been deposited according to the journal's guidelines (see 'Data Deposition' section) and the respective accession numbers provided in the Data Availability Section?	Not Applicable	
Were **human clinical and genomic datasets** deposited in a public access-controlled repository in accordance to ethical obligations to the patients and to the applicable consent agreement?	Not Applicable	
Are **computational models** that are central and integral to a study available without restrictions in a machine-readable form? Were the relevant accession numbers or links provided?	Not Applicable	
If publicly available data were reused, provide the respective **data citations in the reference list**.	Not Applicable	
